# Novel *N*-Acyl Hydrazone Compounds as Promising Anticancer Agents: Synthesis and Molecular Docking Studies

**DOI:** 10.1021/acsomega.3c02361

**Published:** 2023-05-20

**Authors:** Yağmur Biliz, Belma Hasdemir, Hatice Başpınar Küçük, Merve Zaim, Ahmet Mesut Şentürk, Aynur Müdüroğlu Kırmızıbekmez, İhsan Kara

**Affiliations:** † Institute of Graduate Studies, Istanbul University-Cerrahpaşa, Istanbul 34320, Turkey; ‡ Department of Chemistry, Organic Chemistry Division, Istanbul University-Cerrahpaşa, Avcilar, Istanbul 34320, Turkey; § SANKARA Brain and Biotechnology Research Center, Entertech Technocity, Avcilar, Istanbul 34320, Turkey; ∥ Department of Pharmeceutical Chemistry, Faculty of Pharmacy, Istanbul Biruni University, Topkapı, Istanbul 34010, Turkey; ⊥ Department of Physical Therapy and Rehabilitation, School of Health Sciences, Nisantasi University, Maslak, Istanbul 34398, Turkey

## Abstract

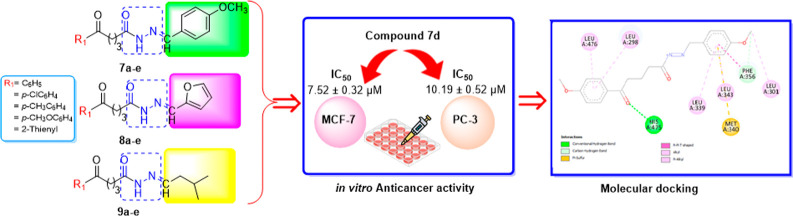

In this study, a new series of *N*-acyl hydrazones **7a-e**, **8a-e**, and **9a-e**, starting from methyl δ-oxo pentanoate with different substituted groups **1a-e**, were synthesized as anticancer agents. The structures of obtained target molecules were identified by spectrometric analysis methods (FT-IR, ^11^H NMR, ^13^C NMR, and LC–MS). The antiproliferative activity of the novel *N*-acyl hydrazones was evaluated on the breast (MCF-7) and prostate (PC-3) cancer cell lines by an MTT assay. Additionally, breast epithelial cells (ME-16C) were used as reference normal cells. All newly synthesized compounds **7a-e**, **8a-e**, and **9a-e** exhibited selective antiproliferative activity with high toxicity to both cancer cells simultaneously without any toxicity to normal cells. Among these novel *N*-acyl hydrazones, **7a-e** showed the most potent anticancer activities with IC_50_ values at 7.52 ± 0.32–25.41 ± 0.82 and 10.19 ± 0.52–57.33 ± 0.92 μM against MCF-7 and PC-3 cells, respectively. Also, molecular docking studies were applied to comprehend potential molecular interactions between compounds and target proteins. It was seen that the docking calculations and the experimental data are in good agreement.

## 1 Introduction

Today, one of the main causes of death worldwide is cancer, which develops when one or more cells from a certain tissue in the body deviate from their usual properties and multiply uncontrollably.^1,2^ According to the findings of recent studies, female breast cancer is the most commonly diagnosed cancer, with 11.7% of cases and 6.9% of all cancer deaths, followed by lung (11.4%), colorectal (10.0%), prostate (7.3%), and stomach (5.6%) cancers.^3,4^

In recent years, intensive studies have been carried out around the world for the development of new drugs that can act against cancer. Today, many factors, such as the rapidly increasing number of patients, serious side effects caused by drugs in use, toxicity, and the development of drug resistance by tumors, increase the importance of these studies. Chemotherapy, one of the anticancer treatments, is widely used because of its effects on tumor cells. However, it is known that many anticancer drugs have serious adverse effects and toxicity. Therefore, it is of great importance to develop new anticancer agents that can stop the growth of cancer cells or kill them while at the same time not harming healthy cells.

Small organic compounds serve as bioactive scaffolds, which are a crucial component of drug design. *N*-Acyl hydrazones, which are an important member of the class of organic compounds and are represented by the general formula R_1_–NHN=CH–R_2_, are small organic molecules in which R_1_ and R_2_ represent different functional groups. In recent years, the interest of researchers has focused on *N*-acyl hydrazones, which have very important pharmacological properties, thus a good option for the development of new biologically active drug molecules.^5−13^

In the research carried out to find more effective and, at the same time, low-toxic anticancer drugs, it was determined that *N*-acyl hydrazone derivatives have anticancer activity, and this result increased the importance of this substance group in cancer treatment. In the literature survey, it is observed that new derivatives of *N*-acyl hydrazones are prepared and their anticancer activities are measured, and these drugs are modified by preparing their analogues with commercially available anticancer drugs.^14−23^ For example, in a study by Popiołek et al., *N*-acyl hydrazones derived from 3-hydroxy-2-naphthoic acid were found to show significant antitumor activities against HepG2 and 769-P cell lines.^24^ In another study, a series of *N*-acyl hydrazone derivatives synthesized from ethyl paraben were reported to have potential activity against liver cancer (HepG2).^25^ The synthesized dipyrromethane *N*-acyl hydrazone derivatives exhibited moderate to extremely strong cytotoxic effects against HL-60 (leukemia) and HCT-116 (colon) cancer cells according to a different study by Gautam et al.^26^ Novel (*R*,*S*)-etodolac derivatives containing the *N*-acyl hydrazone moiety synthesized by Koç et al. have been found to have good cytotoxic effects against PC-3, DU-145, and LNCaP cell lines (prostate cancer).^27^

**Figure 1:**
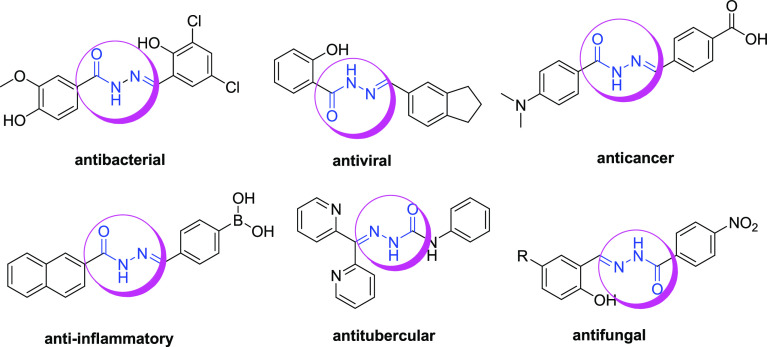
Some *N*-acyl hydrazone compounds for specific targeting applications.

Further, *N*-acyl hydrazones are well-known to exhibit a wide spectrum of biological properties as antioxidant,^28−31^ analgesic,^32^ anti-inflammatory,^33−35^ antimicrobial,^36−41^ antiviral,^42,43^ anticonvulsant,^44^ antiprotozoal,^45^ antimalarial,^46^ larvicidal,^47^ antituberculosis,^48^ and antifungal^49^ activity. Some compounds with known biological activities containing the *N*-acyl hydrazone structure are shown in Figure 1.

**Scheme 1:**
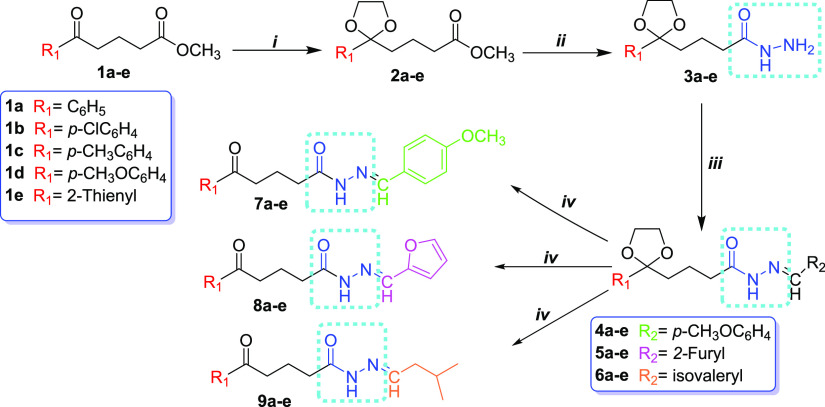
Synthetic Route Was Followed for the Synthesis of the Novel *N*-Acyl Hydrazones Reagents and conditions: (i) Ethylene glycol, triethyl orthoformate, *p*-toluenesulfonic acid monohydrate, toluene, reflux, 24 h (90–96%); (ii) hydrazine monohydrate, EtOH, reflux, 6 h (95–98%); (iii) R_2_CHO (R_2_ = *p*-MeOC_6_H_4_**4a-e**; 2-furyl **5a-e**; isovaleryl **6a-e**), DMF, reflux (51–95%); (iv) Bi(NO_3_)_3_·5H_2_O, CH_2_Cl_2_, rt., 2–4 h (98%).

In the previous study of our group, we synthesized γ- and δ-imino esters from γ- and δ-oxo methyl ester derivatives and found that they exhibited high antioxidant activity.^50^ In another study, we obtained γ-oxime esters and determined that they showed elastase inhibition activity.^51^ Based on our previous studies and literature data, in this work, we focused to designing and synthesizing novel *N*-acyl hydrazones from methyl δ-oxo pentanoate with different substituted groups with promising considerable anticancer properties. For this purpose, we synthesized fifteen novel *N*-acyl hydrazone derivatives **7a-e**, **8a-e**, and **9a-e** from their corresponding methyl δ-oxo pentanoates with aryl, substituted aryl, and heteroaryl groups (Scheme 1). The structures of the compounds **7a-e**, **8a-e**, and **9a-e** were elucidated by FT-IR, ^1^H NMR, ^13^C NMR, and LC–MS analysis methods, and their purity was confirmed by HPLC. Compounds **7a-e**, **8a-e**, and **9a-e** were screened for their in vitro anticancer activity against breast (MCF-7) and prostate (PC-3) cancer cell lines using a 3-(4,5-dimethylthiazol-2-yl)-2,5- diphenyltetrazolium bromide (MTT) assay. In addition, molecular docking studies were applied to investigate the antiproliferative effects of these novel compounds.

## 2 Results and Discussion

### 2.1 Chemistry

In this study, we synthesized fifteen new *N*-acyl hydrazones **7a-e**, **8a-e**, and **9a-e** derived from methyl δ-oxo pentanoates with aryl, substituted aryl, and heteroaryl groups **1a-e** (Scheme 1).

**Figure 2:**
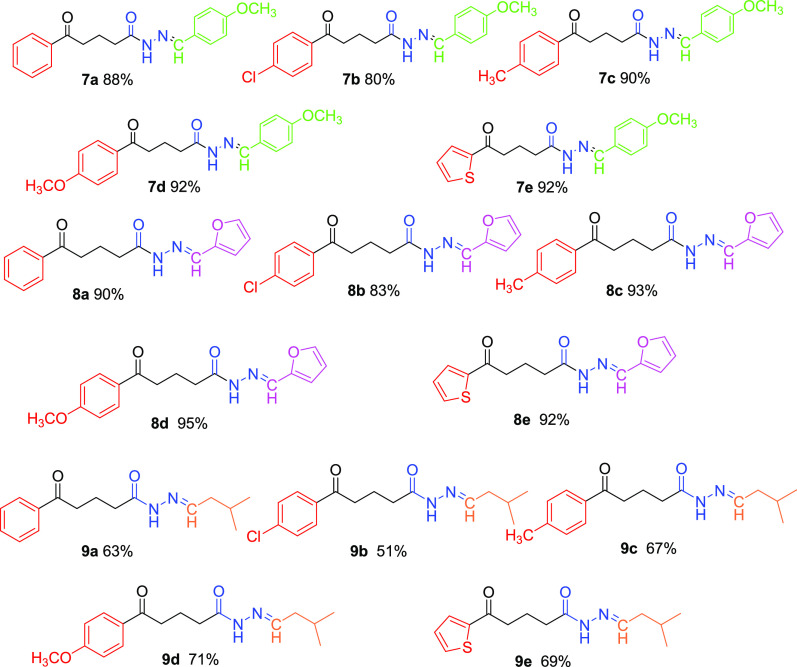
Structure of the target products.

First, we obtained the methyl δ-oxo pentanoate derivatives with substituted aryl and heteroaryl groups **1b-e** used as starting compounds according to the Friedel–Crafts acylation reaction in 85–90% yield.^52,53^ Compound **1a** was obtained from the reaction of δ-oxo-δ-phenyl-pentanoic acid with methanol in the presence of concentrated sulfuric acid in 100% yield. Next, compounds **1a-e** were reacted with hydrazine monohydrate in ethanol in order to obtain δ-oxo pentane hydrazide derivatives as the key intermediate. When we examined the reaction of similar molecules with hydrazine in the literature, we saw that the reaction was carried out without protecting the carbonyl group.^54,55^ However, in our experiments, we observed that both C=O groups in the molecule reacted with hydrazine hydrate, thus reducing the reaction efficiency (45–50%). Thus, we decided to protect the carbonyl group in the δ-position in order to increase the reaction yield and prevent the formation of byproducts. A model reaction was used to determine optimum reaction conditions; we reacted compound **1a** with varying molar ratios of ethylene glycol (EG), triethyl orthoformate (TMOF), and *p*-toluenesulfonic acid monohydrate (*p*-TsOH). The results are given in Table 1. In Table 1, entry 2 shows that the reaction was performed with molar ratios of 1:3:3:0.01 **1a**/EG/TMOF/*p*-TsOH with a 96% yield. Therefore, compounds **2a-e** were successfully obtained in 90–96% isolated yields. Product formation was determined by the GC–MS chromatographic method. Then, ketal the intermediates **3a-e** were prepared by refluxing compounds **2a-e** with hydrazine monohydrate in ethanol in 95–98% yields. The formation of the intermediates **3a-e** was confirmed by the molecular ion peak observed by the GC–MS spectrum. Finally, compounds **3a-e** were reacted with various aldehydes (*p*-anisaldehyde, 2-furaldehyde, and isovaleraldehyde) in dimethylformamide (DMF) in the refluxing condition to generate δ-ketal *N*-acyl hydrazones **4a-e**, **5a-e**, and **6a-e** in 51–95% isolated yields.^56,57^ Then, the hydrolysis reaction of **4a** was investigated in the presence of several acid catalysts and solvents in order to optimize reaction conditions. The results are shown in Table 2. In Table 2, entry 4 shows that the reaction was carried out in the presence of Bi(NO_3_)_3_·5H_2_O in CH_2_Cl_2_ at room temperature with a 98% yield. According to these reaction conditions, *N*-acyl hydrazone derivatives **7a-e**, **8a-e**, and **9a-e** were obtained with 95–98% isolated yields. All the target molecules **7a-e**, **8a-e**, and **9a-e** are shown in Figure 2.

**Table 1:**
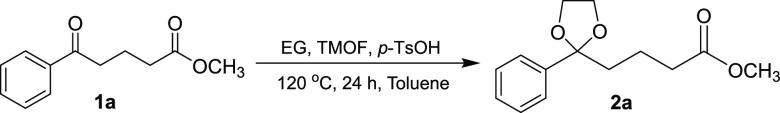
Reaction Condition Experiments for Ketalization

molar ratio				
entry	**1a**:EG	**1a**:TMOF	**1a**:*p*-TsOH	conv. (%)^a^
1	1:1.1	1:0	1:0.05	25
**2**	**1:3**	**1:3**	**1:0.01**	**96**
3	1:5	1:3	1:0.01	75
4	1:5	1:3	1:0.1	82

a Determined by GC–MS

**Table 2:**
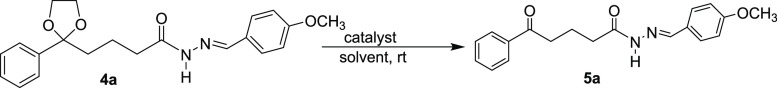
Reaction Condition Experiments for Deprotection of Ketal *N*-Acyl Hydrazones

entry	catalyst	solvent	time	conv (%)^e^	by product (conv. %)^e^
1	*p*-TsOH^a^	acetone:water	40 min	45	38
2	*p*-TsOH^b^	acetone:water	40 min	49	35
3	1 M HCl^c^	THF	3 h	—	—
**4**	**Bi(NO_3_)_3_·5H_2_O**^d^	**CH_2_Cl_2_**	**2 h**	**98**	—

a Molar ratio of **4a**:catalyst = 1:1.

b Molar ratio of **4a**:catalyst = 1:0.5.

c Molar ratio of **4a**:catalyst = 1:0.01.

d Molar ratio of **4a**:catalyst = 1:0.25.

e Determined by GC–MS.

The molecular structures of synthesized *N*-acyl hydrazones **7a-e**, **8a-e**, and **9a-e** were identified through spectroscopic methods (Figures S1–S45, Supporting Information). The purity of these compounds was determined at 97.8–100% by HPLC analysis (Figures S46–S60, Supporting Information).

In the FT-IR spectra, weak −NH bands were observed at 3279–3172 cm^–1^ and N–N bands at 1178–1015 cm^–1^ in all target compounds. Carbonyl absorption (C=O), belonging to both the azomethine group and the aliphatic chain, was seen between 1515–1477 and 1684–1556 cm^–1^, respectively. The peaks around 1596–1488 cm^–1^ are evidence of the presence of the C=N group. The peaks of aromatic ring vibrations were observed between 3090–3026 and 1608–1538 cm^–1^. The literature suggests that the hydrazones could exist as *cis*/*trans* amide conformers and *E*/*Z* geometrical isomers centered on C–N double bonds.^25,58^ In the present study, the peaks observed in the ^1^H and ^13^C NMR spectra of the *N*-acyl hydrazone compounds showed that **7a-e** and **8a-e** were obtained as a mixture of *E* and *Z* isomers. However, compounds **9a-e** were determined to be in single isomer form. Therefore, we evaluated the signals of the ^1^H NMR spectra of the compounds **7a-e** and **8a-e** as set I and set II.^58^ In each compound’s ^1^H NMR spectrum for **7a-e**, **8a-e**, and **9a-e**, the characteristic signals of the azomethine =CH protons found at δ 11.23–8.98 ppm were observed as broad singlets and those of −NH amide protons (−CONHNCH−) as singlets and triplets at δ 7.89–7.09 ppm. Aromatic protons are seen as a doublet, triplet, and multiplet between δ 7.98 and 6.56 ppm. The chemical shift and integral values of other protons are in agreement with their compound structures. In the ^13^C NMR spectra, the azomethine group (−N=CH−) signals were detected at δ 168.7–146.3 ppm. The signals corresponding to C=O of ester and amide were observed at δ 200.2–192.7 and δ 175.2–174.2 ppm, respectively. Other aliphatic and aromatic carbon signals of *N*-acyl hydrazones **7a-e**, **8a-e**, and **9a-e** were seen at the expected values of chemical shift.

### 2.2 Cytotoxicity Assay

In this work, cells were treated with the compounds at concentrations ranging from 1 to 1000 M for 48 h in order to evaluate the in vitro cytotoxic effects on cancer (MCF-7, PC-3) and normal (ME-16C) cell lines. Doxorubicin, the most commonly used chemotherapy drug to treat various types of cancer cells, was chosen as the positive control agent. According to the results, nearly all compounds possess cytotoxic activity against cancer cells with IC_50_ values ranging from 7.52 ± 0.32 to 510.19 ± 2.88 μM (Table 3). Among selected cancer cell lines, MCF-7 cells were more sensitive to the antiproliferative effects of newly synthesized *N*-acyl hydrazones. Doxorubicin demonstrated significantly low IC_50_ values at 0.83 ± 0.07, 0.75 ± 0.04, and 0.80 ± 0.09 for MCF-7, PC-3, and ME-16C cell lines, respectively (Table 3, entry 16). Although doxorubicin seems to be very effective in killing cancer cells at low concentrations, it is also highly toxic to normal cells at the same concentration.

**Table 3:** In Vitro Cytotoxic Activity of Newly Synthesized *N*-Acyl Hydrazone Derivatives^a^

IC_50_ (μM)				
entry	comp. no	MCF-7	PC-3	ME-16C
**1**	**7a**	**25.41 ± 0.82**	**57.33 ± 0.92**	**545.32 ± 0.75**
**2**	**7b**	**10.27 ± 0.63**	**15.00 ± 0.40**	**158.37 ± 1.90**
**3**	**7c**	**9.25 ± 0.54**	**12.57 ± 0.67**	**227.65 ± 1.76**
**4**	**7d**	**7.52 ± 0.32**	**10.19 ± 0.52**	**250.43 ± 1.88**
**5**	**7e**	**12.54 ± 0.84**	**10.81 ± 0.71**	**672.18 ± 2.69**
6	8a	10.98 ± 1.21	26.57 ± 0.92	471.76 ± 2.98
7	8b	50.75 ± 0.63	51.95 ± 0.88	ND
8	8c	471.53 ± 0.57	510.13 ± 1.10	480.53 ± 2.05
9	8d	510.19 ± 2.88	454.71 ± 2.31	ND
10	8e	490.65 ± 3.49	500.52 ± 2.83	ND
11	9a	120.25 ± 1.87	ND	ND
12	9b	210.00 ± 3.14	275.82 ± 5.9	ND
13	9c	95.02 ± 0.76	106.32 ± 3.7	ND
14	9d	115.95 ± 2.7	152.14 ± 0.75	ND
15	9e	100.08 ± 1.39	125.46 ± 1.1	ND
16	doxorubicin	0.83 ± 0.07	0.75 ± 0.04	0.80 ± 0.09

a IC_50_: The concentration that inhibits 50% of cell proliferation; ND: Not determined; ± values represent the standard deviation of the mean.

**Figure 3:**
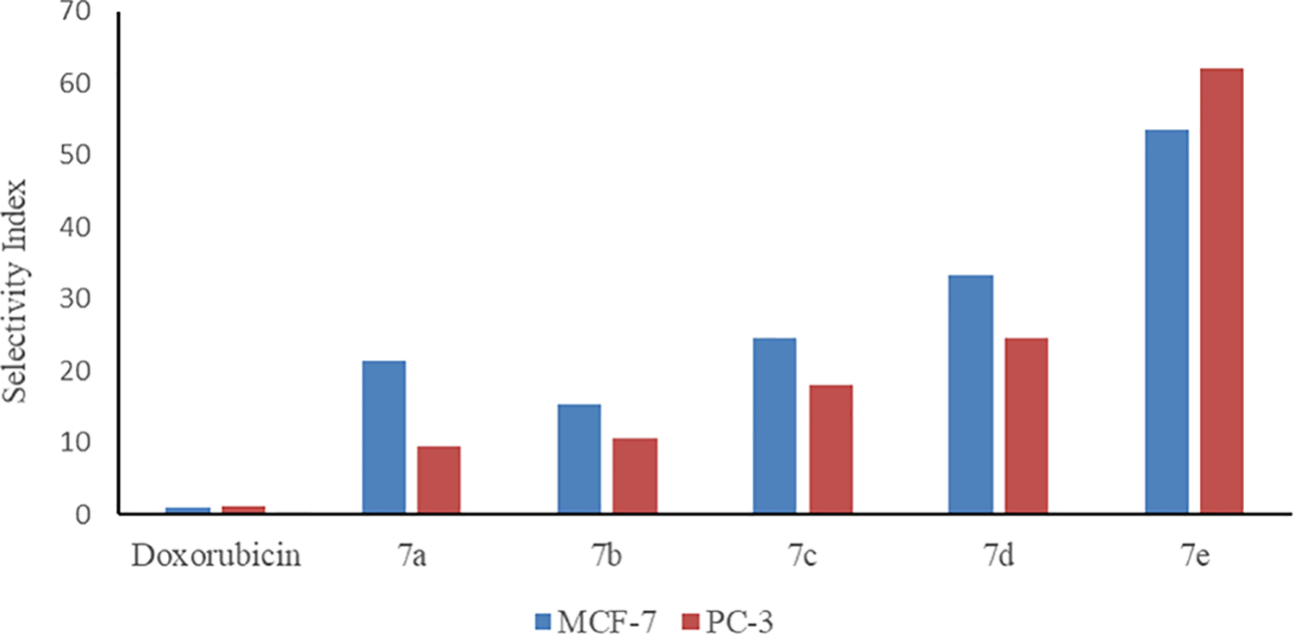
SI of synthesized *N*-acyl hydrazone derivatives **7a-e**.

Compounds **7a-e** exhibited more potent cytotoxic activity in cancer cells than compounds **8a-e** and **9a-e**. Therefore, the selectivity index (SI) of compounds **7a-e** was calculated and is presented in Figure 3. SI is a ratio of the IC_50_ value in cancer cell lines (MCF-7, PC3) to the IC_50_ value in non-cancer cell lines (ME16C). SI values greater than 1.0 demonstrate higher anticancer activity.^59^ The data revealed that compound **7d** was the most cytotoxic compound with IC_50_ values at 7.52 ± 0.32 and 10.19 ± 0.52 μM against MCF-7 and PC-3 cells, respectively (Table 3, entry 4). Moreover, **7d** selectively inhibited the growth of cancer cell lines exhibiting a SI of 33.30 and 24.57 for MCF-7 and PC-3 cells, respectively (Figure 3). **7e** was also characterized by high cytotoxicity and the greatest selectivity with 53.60 and 62.18 SI values for MCF-7 and PC-3 cells, respectively (Figure 3). These results suggest that compounds **7d** and **7e** are promising because of their elevated antiproliferative activity along with considerable selectivity (Table 3, entry 5).

The cytotoxic activity of synthesized *N*-acyl hydrazones in cancer cell lines differed among cells. **7d**, **7c**, and **7b** exhibited the highest antiproliferative effects in MCF-7 cells, whereas **8c**, **8e**, and **8d** were the lowest toxic compounds, respectively. On the other hand, the cytotoxicity order differs in PC-3 cells as **7d**, **7e**, and **7c** were characterized as the most toxic compounds, whereas **8d**, **8e**, and **8c** were the least toxic compounds. The methoxy group in the phenyl ring attached to the azomethine group at the R_2_ position activates the ring due to its electron donor effect. As a result of this effect, it can be assumed that compounds **7a-e** carrying the p-methoxy phenyl group in the R_2_ position have higher antiproliferative activity than other compounds (**8a-e** and **9a-e**). Among synthesized *N*-acyl hydrazones, **8b**, **8d**, **8e**, and **9a-e** did not cause any cytotoxic effect in ME-16C cells in the tested range of concentrations. As a result, it was revealed that the *p*-methoxy phenyl group at the R_2_ position (R_1_–NHN=CH–R_2_) is crucial for inducing cytotoxicity as well as selectivity against the MCF-7 cancer cell line. By replacing the *p*-methoxy phenyl group with furyl or isovaleryl groups, cytotoxic activities were reduced.

### 2.3 Molecular Docking Studies

By molecular docking experiments, these novel compounds’ antiproliferative effects have been examined, and comprehensive evaluations of the molecules’ ideal poses have been conducted. By evaluating the best binding affinity and receptor-ligand interaction of each compound, the good interactions of the compounds within the receptor active pocket of the target receptor proteins are shown in Tables 4 and 5. Considering prior studies of identical structures that contain hydrazone subunits, we made the decision to look for potential binding motifs for MCF-7 and PC-3 in order to examine their anticancer properties for breast cancer (MCF-7) and prostate cancer (PC-3).^60,61^ Thus, the proteins with the highest affinity for the target molecules were determined as 1Z5M for PC-3 and 1X7B for MCF-7. Doxorubicin, an often-used anticancer medicine, was selected to compare our results.

**Table 4:**
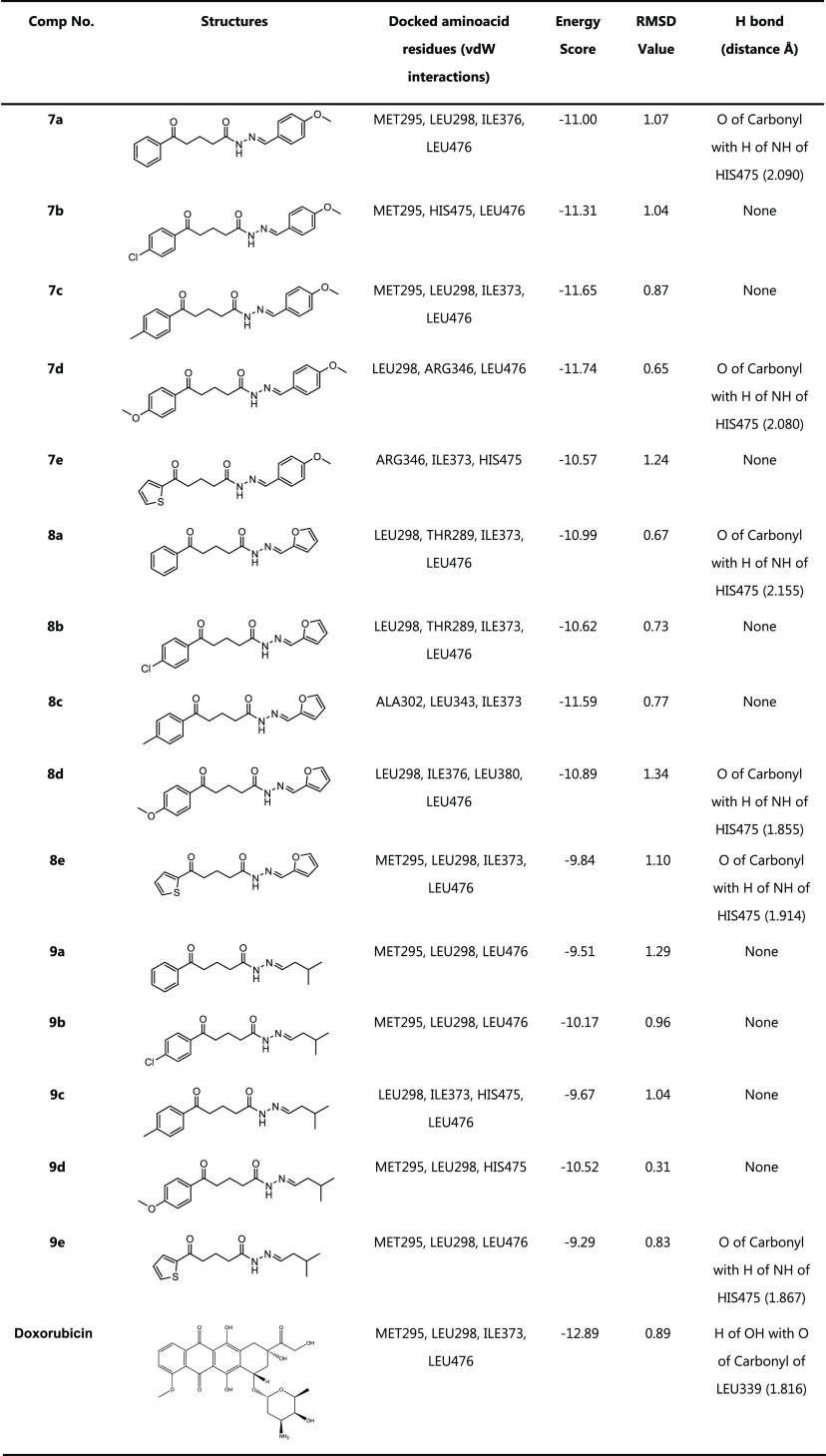
Results of MCF-7 Breast Cancer-1X7B Docking

**Table 5:**
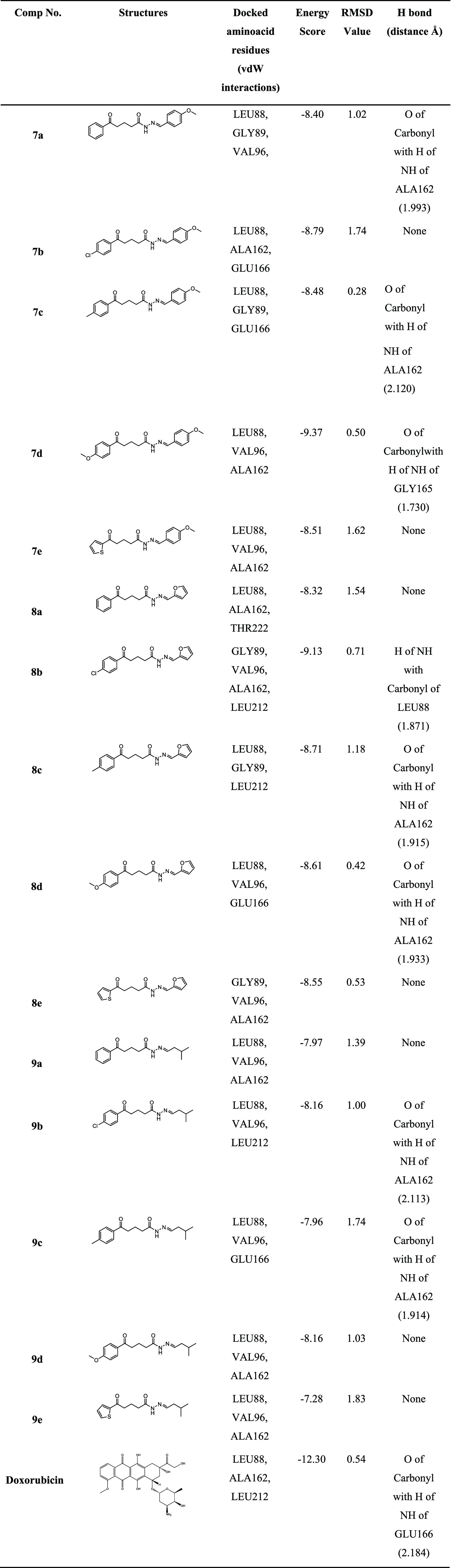
Results of PC-3 Prostate Cancer-1Z5M Docking

After the docking interactions, the conformation with relatively low docking energy scores is selected because the ligand’s strongest binding potential inside the target is indicated by the conformation with the lowest negative binding energy values.

**Figure 4:**
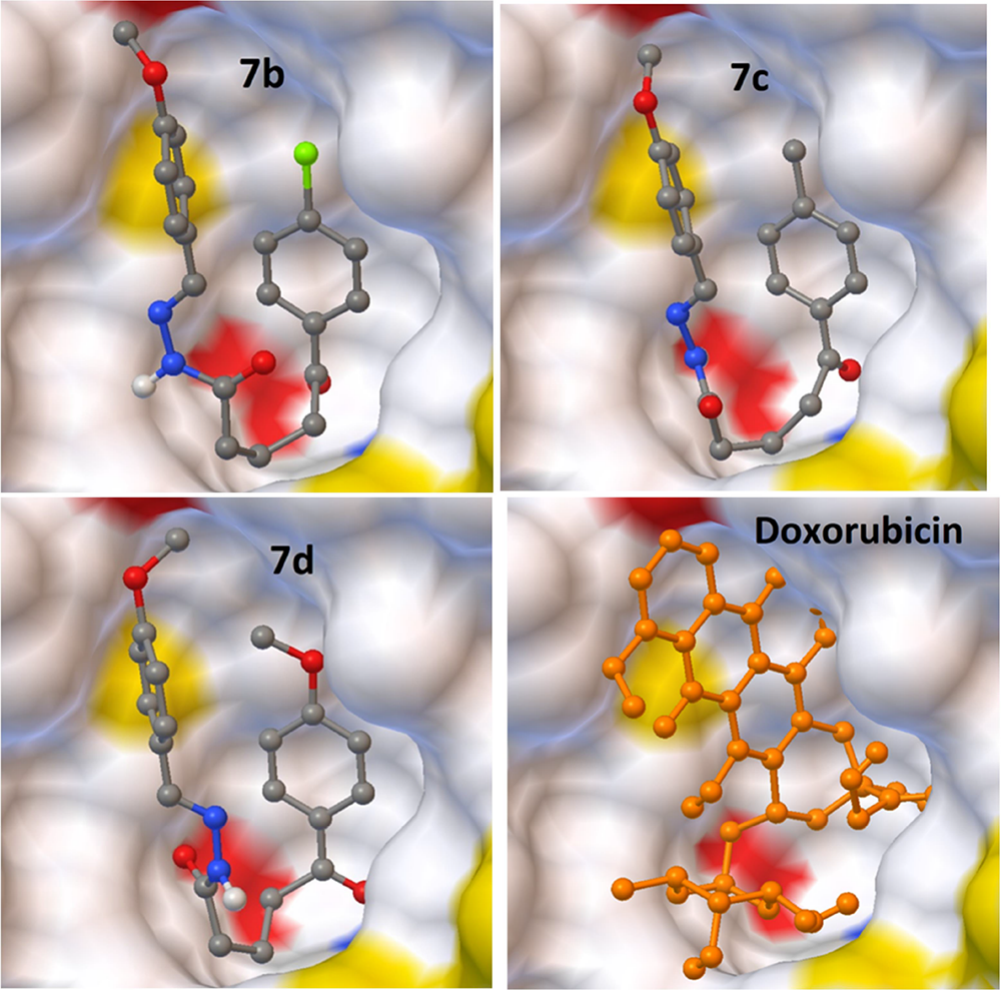
Interaction of the best-docked poses of the reference drug doxorubicin and compounds **7b-d** with the 1X7B target.

**Figure 5:**
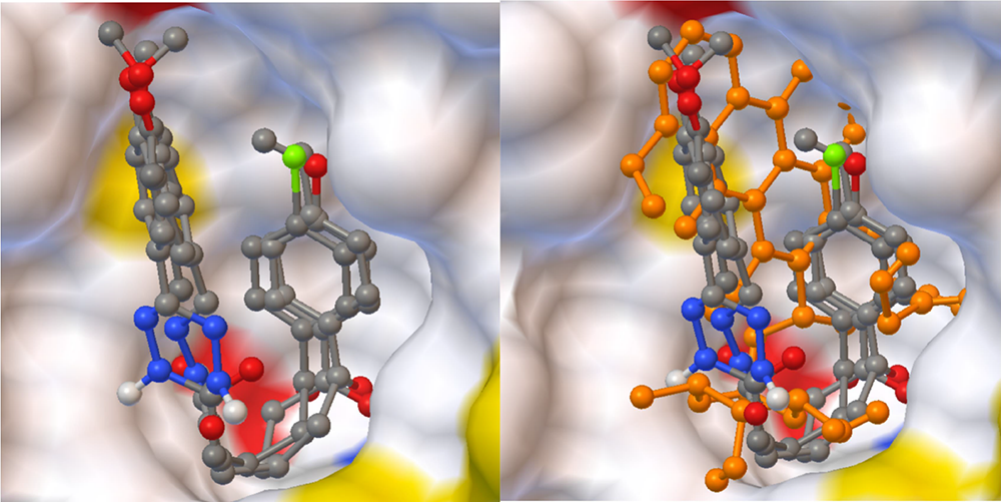
Superimposing poses of best-scored compounds with and without reference drug doxorubicin against breast cancer.

**Figure 6:**
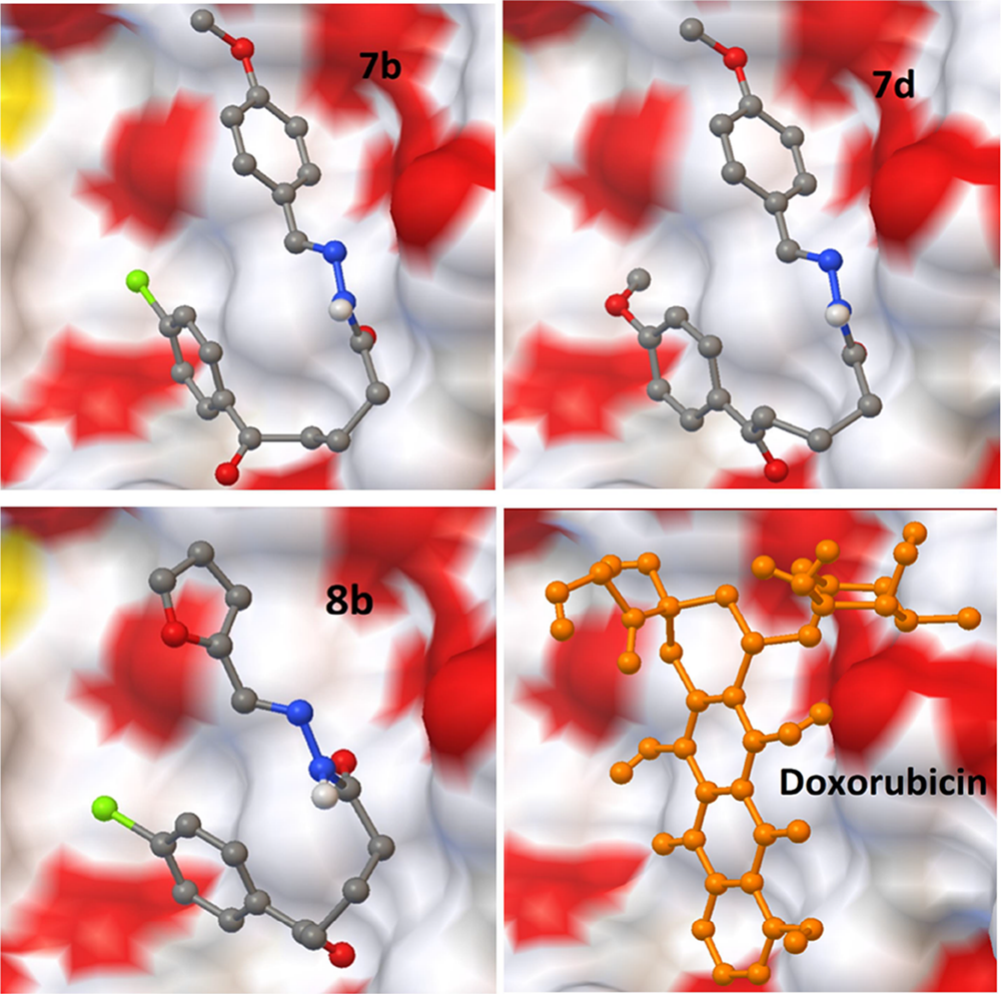
Interaction of the best-docked poses of compounds **7b**, **7d**, **8b** and reference drug doxorubicin to 1Z5M target.

**Figure 7:**
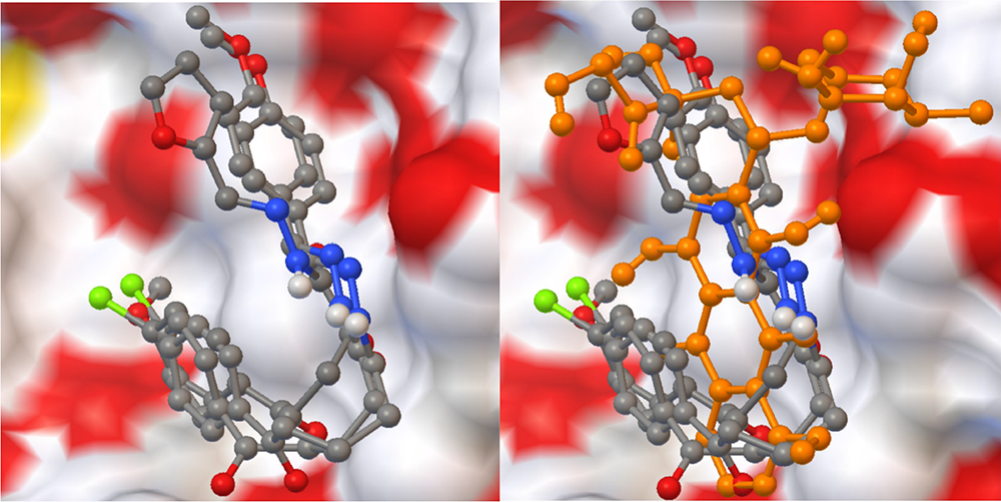
Superimpsing poses of best-scored compounds with and without reference drug doxorubicin against prostate cancer.

Nearly all of the compounds demonstrated adequate binding free energies for MCF-7 and PC-3 that ranged between −9.67 and −11.74 and −7.28 and −9.37 Kcal/mol, respectively. Figures 4–7 show that the compounds bind to the active position and overlap with the reference compounds. According to our preliminary findings, these substances have a respectable level of ligand-receptor binding interactions.

**Figure 8:**
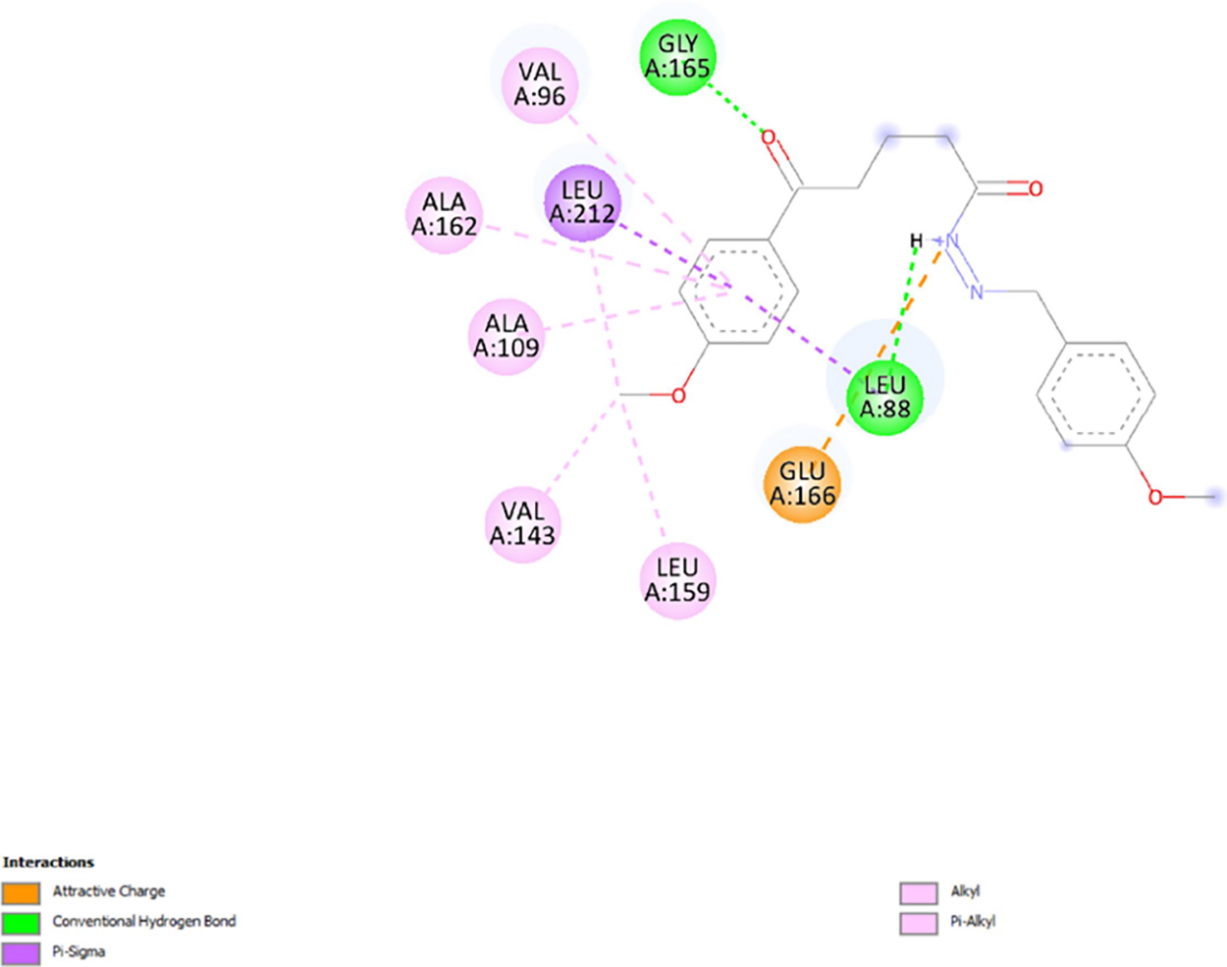
2D diagram of interactions involved between 1Z5M and **7d**.

**Figure 9:**
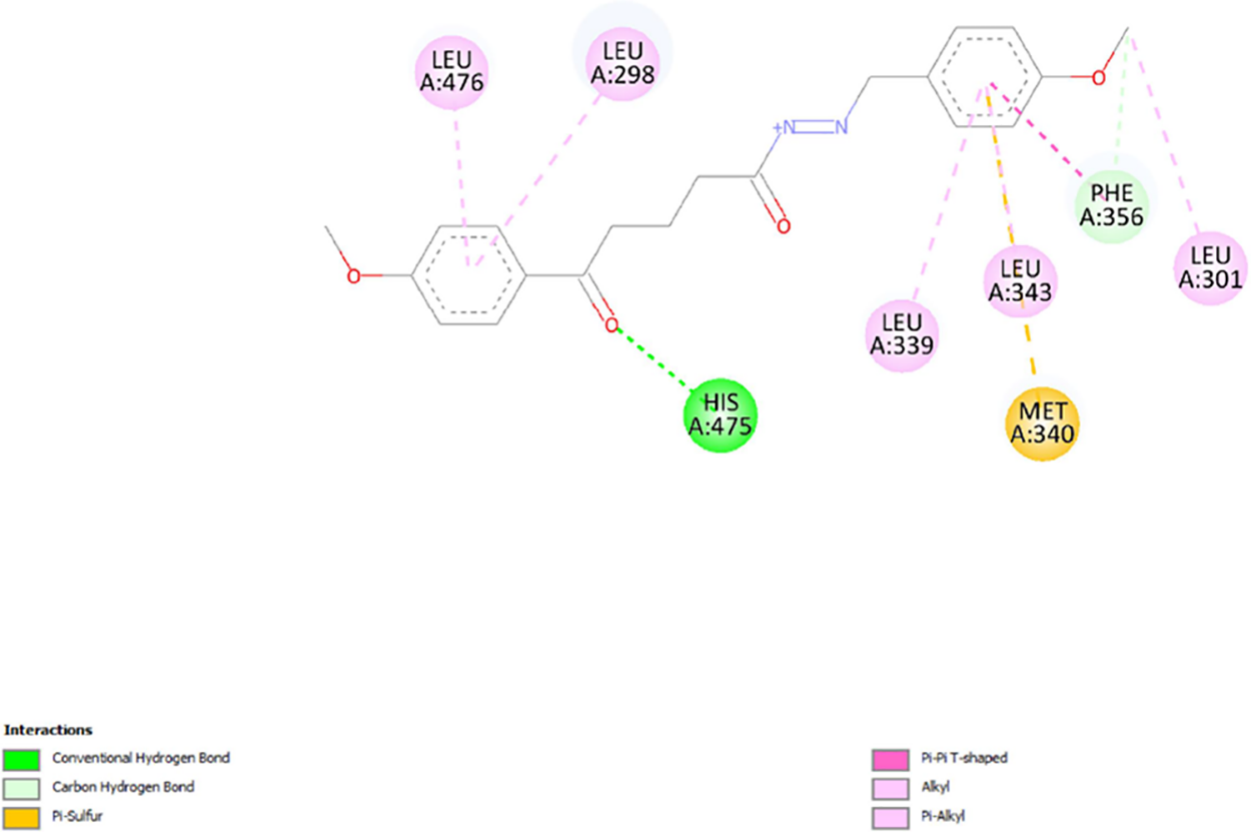
2D diagram of interactions involved between 1X7B and **7d**.

Compounds **7b-d** and **8b** have the smallest RMSD scores with the lowest binding energy scores in each target. As shown in the tables, some of them also exhibited powerful hydrogen bonding with related amino acid residues. The figures show that the active conformations of each compound bind the active site and overlap with each other. The results established that these compounds forecasted the best ligand-receptor binding interactions. Additionally, we evaluated the interactions involved between the target proteins (1Z5M and 1X7B) and compound **7d** which exhibited high affinity toward 1Z5M and 1X7B and has proven to possess good in vitro anticancer activity. Figures 8 and 9 show the interactions between ligands and proteins. Compound **7d** interacts with nine residues to be complexed with 1Z5M as seen in Figure 8. It implicated five alkyl interactions with VAL A: 96, ALA A: 109, VAL A: 143, LEU A: 159, and ALA A: 162; one Pi-sigma interaction with LEU A: 212; attractive charges with GLU A:166 and hydrogen bonds with GLY A:165 and LEU A:88 residues which interact with compound **7d** were identified to be relevant in the complexation process between **7d** and 1Z5M. In Figure 9, compound **7d** interacts with eight residues to be complexed with 1X7B. It implicated five alkyl interactions with LEU A: 298, LEU A: 301, LEU A: 339, LEU A: 343, and LEU A: 476; one carbon-hydrogen bond with PHE A: 356; one pi-sulfur bond with MET A:340; and a hydrogen bond with HIS A:475.

### 2.4 Drug-like Properties

The Swiss ADME Calculation program has been used to determine drug-likeness rankings in order to better understand the structure–activity correlations of compounds. Table 6 lists the molecular weight, logP, TPSA, blood–brain barrier (BBB) crossing, GI absorption characteristics, and kind of CYPP450 inhibition of drugs. Almost all substances have been shown to have quite low levels that will cross lipid barriers. Compounds have been identified to have lipophilicity values less than 4.

**Table 6:** Drug-like Characteristics of Compounds **7a-e**, **8a-e**, and **9a-e** Computed by the Swiss ADME Online Software Program

comp no.	*M*_W_ (g/mol)^a^	Log*P*^b^	TPSA^c^	BB^d^	GI Abs^e^	type of CYP Inh^f^	rule of five^g^
**7a**	324.37	2.98	67.76 Å	yes	high	CYP1A2, CYP2C19, CYP2D6	yes
**7b**	358.82	3.57	67.76 Å	yes	high	CYP1A2, CYP2C19, CYP2C9, CYP2D6, CYP3A4	yes
**7c**	338.40	3.30	67.76 Å	yes	high	CYP1A2, CYP2C19, CYP2D6, CYP3A4	yes
**7d**	354.40	2.98	76.99 Å	yes	high	CYP1A2, CYP2C19, CYP2D6, CYP3A4	yes
**7e**	330.40	3.04	96.00 Å	no	high	CYP1A2, CYP2C19, CYP2C9	yes
**8a**	284.31	2.32	71.67 Å	yes	high	CYP1A2, CYP2C19, CYP2C9	yes
**8b**	318.75	2.84	71.67 Å	yes	high	CYP1A2, CYP2C19, CYP2C9	yes
**8c**	298.34	2.66	71.67 Å	yes	high	CYP1A2, CYP2C19, CYP2C9	yes
**8d**	314.34	2.26	80.90 Å	no	high	CYP1A2, CYP2C19, CYP2C9	yes
**8e**	290.34	2.38	99.91 Å	no	high	CYP1A2, CYP2C19, CYP2C9	yes
**9a**	274.36	2.90	58.53 Å	yes	high	CYP2C19	yes
**9b**	308.80	3.39	58.53 Å	yes	high	CYP1A2, CYP2C19	yes
**9c**	288.38	3.21	58.53 Å	yes	high	CYP2C19	yes
**9d**	304.38	2.90	67.76 Å	yes	high	CYP1A2	yes
**9e**	280.39	2.78	86.77 Å	no	high	CYP1A2, CYP2C19	yes
**doxorubicin**	543.52	1.31	206.07 Å	no	low	none	no

a Molecular weight (recommended value <500).

b Logarithm of the partition coefficient of the compound between n-octanol and water (recommended value <5).

c Polar surface area (recommended value ≤140 Å^2^).

d Indicates whether the compound passes BBB or not.

e Degree of gastrointestinal absorption.

f Represents the inhibition of CYP450 subtypes.

g Indicates whether the compound obeys Lipinski’s rule of five or not.

## 3 Conclusions

In summary, we designed, synthesized, and identified novel *N*-acyl hydrazone derivatives **7a-e**, **8a-e**, and **9a-e** and tested them for in vitro cytotoxicity. Overall, this study presents evidence that *N*-acyl hydrazone derivatives exhibit significant and selective antiproliferative activity in cancer cells. When the results obtained were evaluated, it was determined that all compounds had cytotoxic activity against cancer cell lines (MCF-7 and PC-3) with IC_50_ values ranging from 7.52 ± 0.32 to 672.18 ± 2.69 μM. Besides, no antiproliferative effect was observed in healthy cells at this dose range. This result shows that all synthesized compounds have selective cytotoxic properties. Compounds **7a-e** were found to have stronger cytotoxic activity in cancer cells than compounds **8a-e** and **9a-e**. Among compounds **7a-e**, it was determined that compound **7d** was the most active compound with IC_50_ values at 7.52 ± 0.32 and 10.19 ± 0.52 μM against MCF-7 and PC-3 cells, respectively. Moreover, **7d** showed no cytotoxic effect on ME-16C cells at the same concentration, although it strongly inhibited the growth of cancer cell lines. Among the selected cancer cell lines, breast cancer (MCF-7) cells were found to be more sensitive to the antiproliferative effects of newly synthesized *N*-acyl hydrazones.

Molecular docking experiments were used to explore the antiproliferative properties of these new compounds, and appropriate binding free energies of almost all compounds for MCF-7 and PC-3 were determined between −9.67 and −11.74 and −7.28 and −9.37 Kcal/mol, respectively. According to the results obtained from molecular docking studies, it was determined that all synthesized *N*-acyl hydrazone derivatives exhibited very good ligand-receptor binding interactions. It has also been discovered that all compounds have relatively favorable values for crossing lipid barriers (lipophilicity value <4).

Since conventional chemotherapy drugs are highly toxic to normal cells, newly synthesized *N*-acyl hydrazone derivatives could be promising candidates for anticancer drug development through their selective cytotoxic activity. Therefore, the potential anticancer efficacy and mechanism of action of *N*-acyl hydrazone derivatives must be further investigated in vivo.

## 4 Experimental Section

### 4.1 Materials and Apparatuses

All of the chemicals and solvents utilized in the syntheses and in vitro tests were obtained from commercial suppliers (Merck, Sigma-Aldrich, Acros Organics, and Thermo Fisher Scientific) and used without additional purification. Solvents used for chromatography were of technical grade and distilled before use. Thin-layer chromatography (TLC) was used to monitor chemical reactions under 254 nm UV light. The synthesized starting compounds and *N*-acyl hydrazones were purified using column chromatography on silica gel (0.063–0.200 mm) with hexane-ethylacetate. Melting points were measured with a Buchi melting point apparatus B-540. Gas chromatography–mass spectrometry (GC–MS) data were recorded on a Shimadzu QP2010 Plus. The purity of the *N*-acyl hydrazone derivatives was determined on the Shimadzu/DGU-20A5 HPLC apparatus. FT-IR spectra were recorded on Bruker Vertex. ^1^H NMR and ^13^C NMR spectra were recorded at 500 and 126 MHz, respectively. DMSO d_6_ was used as a solvent, and Me_4_Si was used as the internal standard. LC–MS data were recorded on Shimadzu 8040.

### 4.2 Synthesis

#### 4.2.1 General Procedure for the Protection of Methyl δ-Oxo Pentanoate Derivatives (**2a-e**)

To a solution of δ-oxo methyl ester **1a-e** (1 mmol), ethylene glycol (3 mmol), and triethyl orthoformate (3 mmol) in toluene (5 mL) was added *p*-toluenesulfonic acid monohydrate (0.01 mmol). The reaction mixture was heated to reflux for 24 h until all starting material was consumed in the TLC analysis. Next, the reaction mixture was cooled and quenched with a saturated NaHCO_3_ solution. The mixture was extracted with petroleum ether (40–60 °C) three times. The combined organic phases were washed with saturated NaCl solution and then dried over anhydrous Na_2_SO_4_ and concentrated under reduced pressure. The products **2a-e** were obtained as yellow oil in a 90–96% yield.

#### 4.2.2 General Procedure for the Synthesis of δ-Ketal Hydrazides (**3a-e**)

The 80% hydrazine monohydrate (3 mL) was added to a solution of protected δ-oxo methyl ester **2a-e** (5 mmol) in absolute ethanol (15 mL) and was stirred at reflux for 6 h. The reaction mixture was monitored by TLC (eluent: hexanes/EtOAc 1:1) and visualized using UV light. After the reaction is complete, the ethanol was removed under reduced pressure. The mixture was extracted with ethyl acetate three times. The organic phase was washed with distilled water, dried over anhydrous Na_2_SO_4_, and concentrated under reduced pressure. The ketal hydrazides **3a-e** were obtained in a 95–98% isolated yield.

#### 4.2.3 General Procedure for the Synthesis of δ-Ketal *N*-Acyl Hydrazones (**4a-e**, **5a-e**, **6a-e**)^56,57^

The δ-ketal hydrazide **3a-e** (1 mmol) and aldehyde (anisaldehyde, furfural, or isovaleraldehyde) (2 mmol) in DMF (2 mL) were added to a reaction flask, and then the mixture was heated to reflux. The reaction progress was monitored by TLC (eluent: hexane:EtOAc (1:1). After finishing the reaction, the solvent was removed under reduced pressure. The crude product was purified by column chromatography on silica gel (eluent: hexanes/EtOAc 3:1). The pure products **4a-e**, **5a-e**, and **6a-e** were obtained in 51–95% isolated yields.

#### 4.2.4 General Procedure for the Synthesis of *N*-Acyl Hydrazone Derivatives by Deprotection of δ-Ketal *N*-Acyl Hydrazones (**7a-e**, **8a-e**, **9a-e**)

A solution of δ-ketal *N*-acyl hydrazones **4a-e**, **5a-e**, **6a-e** (1 mmol) and Bi(NO_3_)_3_·5H_2_O (0.25 mmol) in CH_2_Cl_2_ (5 mL) was stirred at room temperature for 2–4 h. Next, the mixture was filtered, and the filtrate was washed first with a 10% aqueous NaHCO_3_ solution and then with saturated NaCl, dried over anhydrous Na_2_SO_4_, and concentrated under reduced pressure. The pure products **7a-e**, **8a-e**, and **9a-e** were obtained in a 98% isolated yield.

### 4.3 HPLC Analysis

The purity of the target compounds was defined by normal phase high-performance liquid chromatography (HPLC) using a 250 × 4,6 mm, 5 μm AD-H chiral column using Hekzan/isopropanol (70:30). The sample (10 μL) was injected into the column with 1 mL/min flow rate.

### 4.4 Biological Section

#### 4.4.1 Cell Culture

Human breast cancer (MCF-7; ATCC Number: HTB-22), prostate cancer (PC-3; ATCC Number: CRL-1435), and breast epithelial (ME-16C; ATCC Number: CRL: 4101) cell lines were procured from American Type Culture Collection (ATCC Distributor: LGC Standards, Wessel, Germany) to be used in the cytotoxic activity assays. Cells were detached with a 0.25% Trypsin/EDTA solution upon 70–80% confluency and seeded into a new culture flask including DMEM-LG with 10% FBS and 0.1 mg/mL primocin, incubated in a 37 °C, 5% CO_2_ incubator. The medium was refreshed every 48 h, and the cells were passaged every 5–6 days.

#### 4.4.2 MTT Assay

Newly synthesized compounds were tested for their cytotoxic activity on cancer (MCF-7, PC-3) and normal (ME-16C) cell lines using the MTT assay. MTT is used to assess cell viability by determining the mitochondrial activity of living cells according to their ability to reduce the yellow tetrazolium salt MTT into purple formazan crystals. The MTT assay (Thermo) was carried out according to the manufacturer’s instructions. Briefly, cells were seeded on 96-well plates at a density of 1 × 10^4^ cells/well and incubated in a 37 °C, 5% CO_2_ incubator. Cells were then treated with different concentrations (1–1000 μM) of compounds for 48 h. Following incubation, 10 μL of MTT solution was added to the cells and incubated for 4 h, and then 100 μL of solubilization solution was added and incubated overnight. Absorbance was measured at 570 nm using the microplate reader (Synergy H1, Biotek). For all experiments, doxorubicin was used as the positive control, and untreated cells were used as the negative control. DMSO was used as the solvent of the compounds, and the final concentration did not exceed 0.5% v/v. All the experiments were performed three times, and each was carried out in triplicate.

### 4.5 Molecular Docking Procedure

To provide a theoretical perspective on probable molecular interactions between compounds from the **7a-e**, **8a-e**, and **9a-e** series and target proteins, molecular docking experiments were conducted. Energy minimization was used to calculate theoretical binding affinities based on the results of docking calculations. The computation of molecular docking, energy reduction, and molecular visualization of docking data was carried out using the Autodock Vina software suite. The Chem Draw drawing program was used to prepare model inhibitor compounds for molecular docking in the **7a-e**, **8a-e**, and **9a-e** series. Before the docking procedure, the special **7a-e**, **8a-e**, and **9a-e** series compounds were drawn and edited in the SD File format using the Chem 3D suite program. Protonation, charging, and conformation minimization utilizing the root-mean-square gradient have all been applied to these molecular structures.

Three-dimensional coordinates of X-ray crystal structures of target proteins were obtained from the Structural Bioinformatics Research Collaboration (RCSB) Protein Data Bank.^62^ For use in docking calculations, a structure with the PDB IDs 1X7B for MCF-7 and 1Z5M for PC-3 was selected as the crystal structure model matching these target proteins. The software tool Autodock Vina was used to correct structural defects in these target proteins. As docking calculations are being performed, default parameters are being used (temperature 300 Kelvin, pH 7, solvent 0.1 M, electrostatic energy cutoff 15 A). The final molecular docking score values were calculated using the average score of the top 10 final docking postures determined by the binding minimum energy (kcal/mol) for each molecule.^62,63^

## Supporting Information Available

Experimental details; general procedures; experimental characterization data (IR, NMR, LC–MS, and HPLC purity); HPLC chromatograms; FT-IR spectra and ^1^H, ^13^C NMR spectra of all target products (PDF)
