# Supplementary material for: Novel N-Acyl Hydrazone Compounds as Promising Anticancer Agents: Synthesis and Molecular Docking Studies
Source: ACS Omega. 2023 May 20;8(22):20073–84. doi: 10.1021/acsomega.3c02361 (PMC10249086; doi:10.1021/acsomega.3c02361)
Supplement: Supplementary file 1 — ao3c02361_si_001.pdf [file ao3c02361_si_001.pdf]

Supporting Information for

Novel *N*-acyl hydrazone compounds as promising anticancer agents: synthesis and molecular docking studies

Yağmur Biliz[a], Belma Hasdemir*[,b], Hatice Başpınar Küçük[b], Merve Zaim[c], Ahmet Mesut Şentürk[d] Aynur Müdüroğlu Kırmızıbekmez[d], İhsan Kara[c]

[a] *Istanbul University-Cerrahpaşa, Institute of Graduate Studies, Istanbul, Turkey*

[b] *Department of Chemistry, Faculty of Engineering, Istanbul University-Cerrahpaşa, Istanbul, Turkey*

[c]*SANKARA Brain and Biotechnology Research Center, Entertech Technocity, Avcilar, Istanbul, Turkey*

[d]*Istanbul Biruni University Department of Pharmeceutical Chemistry, Faculty of Pharmacy, 34010 Topkapı, Istanbul/Turkey*

[e]*Department of Physical Therapy and Rehabilitation, School of Health Sciences, Nisantasi University, Maslak, Istanbul, Turkey*

E-mail: b.hasdemir@iuc.edu.tr

Content

Materials and Apparatuses.. S2

General procedure for protection methyl δ-oxo pentanoate derivatives……………….. S2

General procedure for the synthesis of δ-ketal hydrazides……………………………… S3

General procedure for the synthesis of δ-ketal *N*- acyl hydrazones…………………… S3

General procedure of the synthesis of *N*-acyl hydrazone derivatives by deprotection of δ-ketal *N*-acyl hydrazones.. S3

Experimental Characterization Data of 7a-e, 8a-e and 9a-e…………………………… S4

IR and NMR Spectra of all the synthesized compounds……………………………… S11

HPLC chromatograms of all the synthesized compounds……………………………… S34

1. Materials and Apparatuses

All of the chemicals and solvents utilized in the syntheses and in vitro tests were obtained from commercial suppliers (Merck, Sigma- Aldrich, Acros Organics, and Thermo Fisher Scientific) and used without additional purification. Solvents used for chromatography were of technical grade and distilled before use. Thin-layer chromatography was used to monitor chemical reactions under 254 nm UV light. The synthesized starting compounds and N-acyl hydrazones were purified using column chromatography on silica gel (0.063–0.200mm) with hexane-ethylacetate. Melting points were measured with a Buchi melting point apparatus B-540. Gas chromatography-mass spectrometry (GC-MS) data were recorded on a Shimadzu QP2010 Plus. The purity of the N-acyl hydrazone derivatives was determined on Shimadzu/DGU-20A5 HPLC apparatus. FT-IR spectra were recorded on Bruker Vertex. 1H NMR and 13C NMR spectra were recorded at 500 MHz and 126 MHz, respectively. DMSO d6 was used as a solvent, and Me4Si was used as the internal standard. LC-MS data were recorded on Shimadzu 8040.

2. General procedure for protection methyl δ-oxo pentanoate derivatives (2a-e)

To a solution of δ-oxo methyl ester **1a-e** (1 mmol), ethylene glycol (3 mmol), and triethyl orthoformate (3 mmol) in toluene (5 mL) was added *p*-toluenesulfonic acid monohydrate (0.01 mmol). The reaction mixture was heated to reflux for 24 hours until all starting material was consumed in the thin-layer chromatography analysis. Next, the reaction mixture was cooled and quenched with a saturated $NaHCO_3$ solution. The mixture was extracted with petroleum ether (40-60°C) three times. The combined organic phases were washed with saturated NaCl solution and then dried over anhydrous Na_2SO_4, and concentrated under reduced pressure. The products **2a-e** were obtained as yellow oil in a 90-96 % yield.

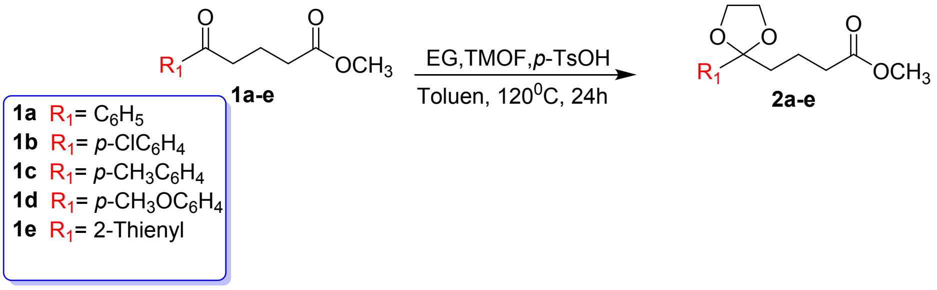

Scheme S1. Synthesis of compounds **2a-e**

3. General procedure for the synthesis of δ-ketal hydrazides (3a-e)

The 80 % hydrazine monohydrate (3 mL) was added to a solution of protected δ-oxo methyl ester **2a-e** (5 mmol) in absolute ethanol (15 mL) and was stirred at reflux for 6 h. The reaction mixture was monitored by TLC (eluent: hexanes/EtOAc 1:1) and visualized using UV light. After the reaction is complete, the ethanol was removed under reduced pressure. The mixture was extracted with ethyl acetate three times. The organic phase was washed with distilled water, dried over anhydrous Na_2SO_4, and concentrated under reduced pressure. The ketal hydrazides **3a-e** was obtained in a 95-98 % isolated yield.

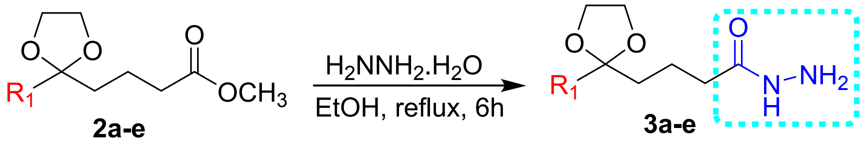

Scheme S2. Synthesis of δ-ketal hydrazides **3a-e**

4. General procedure for the synthesis of δ-ketal *N*-acyl hydrazones (4a-e, 5a-e, 6a-e)[1,2]

The δ-ketal hydrazide **3a-e** (1 mmol) and aldehyde (anisaldehyde, furfural or isovaleraldehyde) (2 mmol) in DMF (2 mL) were added to a reaction flask, then the mixture was heated to reflux. The reaction progress was monitored by TLC (eluent: hexane: EtOAc (1:1). After finishing the reaction, the solvent was removed under reduced pressure. The crude product was purified by column chromatography on silica gel (eluent: hexanes/EtOAc 3:1). The pure products **4a-e, 5a-e, 6a-e** were obtained in 51-95% isolated yield.

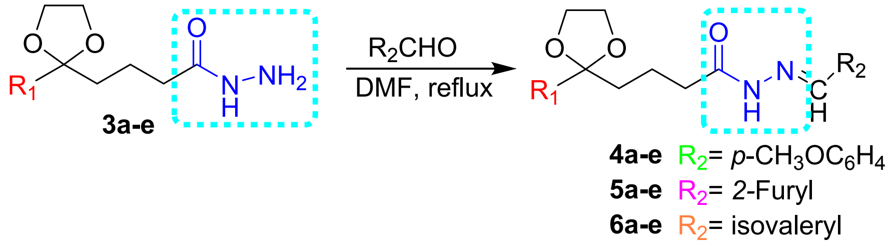

Scheme S3. Synthesis of δ-ketal *N*-acyl hydrazones 4**a-e**, **5a-e**, **6a-e**

5. General procedure of the synthesis of *N*-acyl hydrazone derivatives by deprotection of δ-ketal *N*-acyl hydrazones (7a-e, 8a-e, 9a-e)

A solution of δ-ketal *N*-acyl hydrazones **4a-e**, **5a-e**, **6a-e** (1 mmol) and $Bi(NO_3)_3.5H_2O$ (0.25 mmol) in CH_2Cl_2 (5 mL) was stirred at room temperature for 2-4 h. Next, the mixture was filtered, the filtrate was washed first with 10% aqueous $NaHCO_3$ solution then with saturated NaCl and dried over

anhydrous Na_2SO_4 and concentrated under reduced pressure. The pure products **7a-e**, **8a-e**, **9a-e** were obtained in 98% isolated yield.

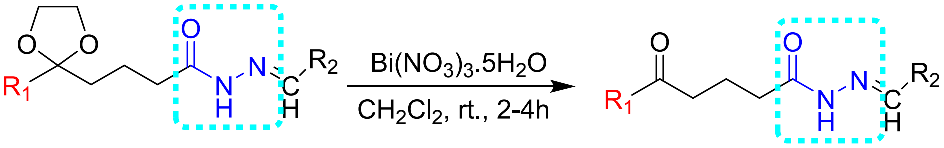

4a-e; 5a-e; 6a-e **7a-e; 8a-e; 9a-e**

Scheme S4. Deprotection of δ-ketal *N*-acyl hydrazones

6. Experimental Characterization Data of 7a-e, 8a-e and 9a-e

N'-(4-methoxybenzylidene)-5-oxo-5-phenylpentanehydrazide (7a)

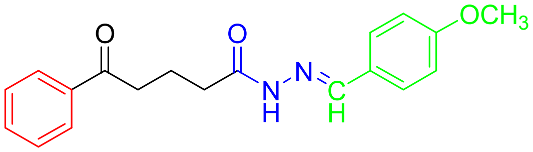

White powder, Yield 88 %, mp 199-200 ºC, IR (ATR) υ_{max}/cm^{-1}: 3279 (NH), 3049 (CH, aryl), 2933 (CH, alkyl), 1663, 1604 (2C=O), 1505 (C=N), 1380 (C-O), 1021 (N-N), 822 (CH, aryl). Two sets of signals observed in NMR experiments. ^{1}H NMR (500 MHz, DMSO) δ: (**first set**) 9.87 (brs, 1H), 7.99 (d, *J* = 7.1 Hz, 2H), 7.78 (s, 1H), 7.58 (d, *J* = 8.8 Hz, 2H), 7.54 (t, *J* = 7.4 Hz, 1H), 7.43 (t, *J* = 7.7 Hz, 2H), 6.90 (d, *J* = 8.8 Hz, 2H), 3.84 (s, 3H), 3.14 (t, *J* = 7.2 Hz, 2H), 2.90 (t, *J* = 7.2 Hz, 2H), 2.24- 2.18 (m, 2H). ^{1}H NMR (500 MHz, DMSO) δ: (**second set**) 9.90 (brs, 1H), 8.10 (s, 1H), 7.85 (d, *J* = 8.8 Hz, 2H), 7.68 (d, *J* = 8.6 Hz, 2H), 7.54 (t, *J* = 7.4 Hz, 1H), 7.43 (t, *J* = 7.7 Hz, 2H), 7.02 (d, *J* = 8.7 Hz, 2H), 3.90 (s, 3H), 3.14 (t, *J* = 7.2 Hz, 2H), 2.90 (t, *J* = 7.2 Hz, 2H), 2.24- 2.18 (m, 2H). ^{13}C NMR (126 MHz, *DMSO*) δ: (**first and second set**) 200.2, 200.1, 174.2, 168.4, 161.1, 160.9, 146.1, 142.9, 137.0, 133.5, 133.5, 129.1, 129.1, 128.9, 128.6, 128.3, 127.2, 114.7, 114.6, 55.6, 37.8, 37.6, 33.6, 31.3, 20.1, 19.6. LC-MS (m/z): 324 (M^+).

N'-(4-methoxybenzylidene)-5-(4-chlorophenyl)-5-oxopentanehydrazide (7b)

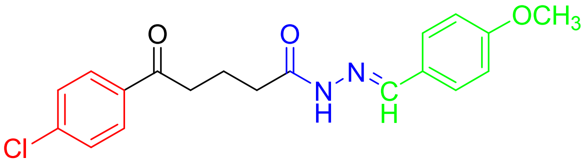

White powder, Yield 80 %, mp 121-122.5 ºC, IR (ATR) υ_{max}/cm^{-1}: 3200 (NH), 3081 (CH, aryl), 2954 (CH, alkyl), 1665, 1607 (2C=O), 1504 (C=N), 1394 (C-O), 1026 (N-N), 819 (CH, aryl). Two sets of

signals observed in NMR experiments. ^{1}H NMR (500 MHz, DMSO) δ: (**first set**) 11.09 (brs, 1H), 7.97 (d, *J*=8.5 Hz, 2H), 7.89 (s, 1H), 7.55-7.52 (m, 4H), 6.95 (d, *J*=8.8 Hz, 2H), 3.77 (s, 3H), 3.09 (t, *J*=7.2 Hz, 2H), 2.67 (t, *J*=7.4 Hz, 2H), 1.93-1.87 (m, 2H). ^{1}H NMR (500 MHz, DMSO) δ: (**second set**) 11.21 (brs, 1H), 8.07 (s, 1H),7.97 (d, *J*=8.5 Hz, 2H), 7.60-7.57 (m, 4H), 6.98 (d, *J*=8.8 Hz, 2H), 3.78 (s, 3H), 3.08-3.05 (m, 2H), 2.26 (t, *J*=7.3 Hz, 2H), 1.93-1.87 (m, 2H). ^{13}C NMR (126 MHz, DMSO) δ: (**first and second set**) 199.2, 199.1, 174.2, 168.4, 160.8, 146.18, 142.9, 138.4, 135.7, 130.2, 129.2, 128.5, 127.3, 114.5, 55.7, 33.6, 31.5, 20.0, 19.2. LC-MS (m/z): 357 (M^+-1).

N'-(4-methoxybenzylidene)-5-oxo-5-p-tolylpentanehydrazide (7c)

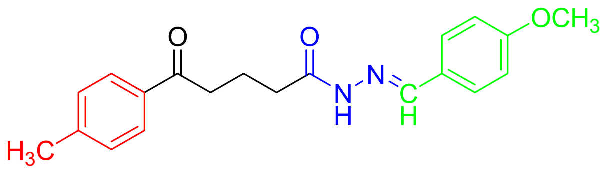

White powder, Yield 90 %, mp 133-134 ºC, IR (ATR) υ_{max}/cm^{-1}: 3244 (NH), 3031 (CH, aryl), 2951 (CH, alkyl), 1681, 1601 (2C=O), 1488 (C=N), 1391 (C-O), 1029 (N-N), 810 (CH, aryl). Two sets of signals observed in NMR experiments. ^{1}H NMR (500 MHz, DMSO) δ: (**first set**) 11.15 (brs, 1H), 7.89 (s, 1H), 7.85 (d, *J* = 8.1 Hz, 2H), 7.54 (d, *J* = 8.7 Hz, 2H), 7.30 (t, *J* = 8.2 Hz, 2H), 6.94 (d, *J* = 8.7 Hz, 2H), 3.77 (s, 3H), 3.05 (t, *J* = 7.3 Hz, 2H), 2.67 (t, *J* = 7.4 Hz, 2H), 2.34 (s, 3H), 1.93-1.87 (m, 2H). ^{1}H NMR (500 MHz, DMSO) δ: (**second set**) 11.15 (brs, 1H), 8.08 (s, 1H), 7.85 (d, *J* = 8.1 Hz, 2H), 7.59 (d, *J* = 8.8 Hz, 2H), 7.30 (t, *J* = 8.2 Hz, 2H), 6.97 (d, *J* = 8.8 Hz, 2H), 3.78 (s, 3H), 3.04-3.00 (m, 2H), 2.35 (s, 3H), 2.26 (t, *J* = 7.4 Hz, 2H), 1.93-1.87 (m, 2H). ^{13}C NMR (126 MHz, DMSO) δ: (**first and second set**) 199.7, 199.5, 174.2, 168.5, 161.0, 160.8, 146.1, 143.7, 142.8, 134.6, 134.6, 129.6, 129.6, 128.9, 128.5, 128.4, 127.3, 114.6, 55.6, 37.7, 37.5, 33.7, 31.7, 21.5, 20.2, 19.5. LC-MS (m/z): 337 (M^+-1).

N'-(4-methoxybenzylidene)-5-(4-methoxyphenyl)-5-oxopentanehydrazide (7d)

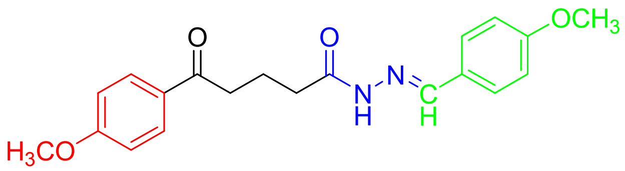

White powder, Yield 92 %, mp 154-155 ºC, IR (ATR) υ_{max}/cm^{-1}: 3238 (NH), 3061 (CH, aryl), 2959 (CH, alkyl), 1668, 1646 (2C=O), 1507 (C=N), 1245 (C-O), 1023 (N-N), 830 (CH, aryl). Two sets of signals observed in NMR experiments. ^{1}H NMR (500 MHz, DMSO) δ: (**first set**) 11.09 (brs, 1H), 7.94 (d, *J* = 8.9 Hz, 2H), 7.89 (s, 1H), 7.54 (d, *J* = 8.8 Hz, 2H), 7.01 (t, *J* = 9.2 Hz, 4H), 3.81 (s, 3H), 3.77 (s, 3H), 3.02 (t, *J* = 7.3 Hz, 2H), 2.67 (t, *J* = 7.4 Hz, 2H) 1.91-1.88 (m, 2H). ^{1}H NMR (500 MHz,

DMSO) δ: (**second set**) 11.21 (brs, 1H), 8.08 (s, 1H), 7.94 (d, J = 8.9 Hz, 2H), 7.59 (d, J = 8.8 Hz, 2H), 6.97-6.94 (m, 4H), 3.82 (s, 3H), 3.78 (s, 3H), 3.01-2.98 (m, 2H), 2.26 (t, J=7.3 Hz, 2H), 1.91-1.88 (m, 2H). ^{13}C NMR (126 MHz, DMSO) δ: (**first and second set**) 198.6, 198.4, 174.2, 168.5, 163.4, 161.0, 160.8, 146.1, 142.8, 130.6, 130.0, 128.9, 128.5, 127.3, 114.6, 114.2, 55.9, 55.7, 37.3, 37.2, 33.7, 31.8, 20.3, 19.7. LC-MS (m/z): 353 (M^+-1).

N'-(4-methoxybenzylidene)-5-oxo-5-(thiophen-2-yl)pentanehydrazide (7e)

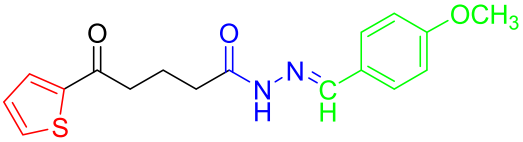

White powder, Yield 92%, mp 130-131 ºC, IR (ATR) υ_{max}/cm^{-1}: 3233 (NH), 3070 (CH, aryl), 2931 (CH, alkyl), 1651, 1604 (2C=O), 1504 (C=N), 1236 (C-O), 1021 (N-N), 827, 714. Two sets of signals observed in NMR experiments. ^{1}H NMR (500 MHz, DMSO) δ: (**first set**) 11.17 (brs, 1H), 7.98 (t, J = 5.7 Hz, 1H), 7.95 (d, J = 3.8 Hz, 1H), 7.91 (s, 1H), 7.55 (d, J = 8.8 Hz, 2H), 7.23-7.21 (m, 1H), 6.96 (d, J = 8.8 Hz, 2H), 3.78 (s, 3H), 3.05 (t, J = 7.3 Hz, 2H), 2.69 (t, J = 7.4 Hz, 2H), 1.94-1.91 (m, 2H). ^{1}H NMR (500 MHz, DMSO) δ: (**second set**) 11.17 (brs, 1H), 8.09 (s, 1H), 7.98 (t, J = 5.7 Hz, 1H), 7.95 (d, J = 3.8 Hz, 1H), 7.61 (d, J = 8.9 Hz, 2H), 7.25-7.24 (m, 1H), 6.99 (d, J = 8.9 Hz, 2H), 3.79 (s, 3H), 3.01 (t, J = 7.2 Hz, 2H), 2.27 (t, J = 7.4 Hz, 1H), 1.94-1.91 (m, 2H). ^{13}C NMR (126 MHz, DMSO) δ: (**first and second set**) 193.4, 193.2, 174.1, 168.4, 161.1, 160.9, 146.1, 144.2, 142.8, 135.1, 135.0, 133.6, 133.5, 129.1, 129.1, 128.9, 128.6, 127.3, 127.3, 114.7, 55.7, 38.3, 38.1, 33.6, 31.7, 20.4, 19.8. LC-MS (m/z): 329 (M^+-1).

N'-((furan-2-yl)methylene)-5-oxo-5-phenylpentanehydrazide (8a)

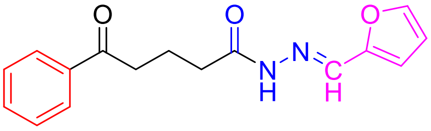

Light Brown powder, Yield 90 %, mp 119-120 ºC, IR (ATR) υ_{max}/cm^{-1}: 3175 (NH), 3089 (CH, aryl), 2962 (CH, alkyl), 1662, 1621 (2C=O), 1399 (C-O), 1015 (N-N), 929, 736. Two sets of signals observed in NMR experiments. ^{1}H NMR (500 MHz, DMSO) δ: (**first set**) 11.22 (brs, 1H), 7.96 (d, J = 7.2 Hz, 2H), 7.84 (s, 1H), 7.76 (s, 1H), 7.53-7.49 (m, 3H), 6.80 (d, J = 3.3 Hz, 1H), 6.58-6.57 (m, 1H), 3.10 (t, J = 7.3 Hz, 2H), 2.64 (t, J = 7.4 Hz, 2H), 1.93-1.86 (m, 2H). ^{1}H NMR (500 MHz, DMSO) δ: (**second set**) 11.22 (brs, 1H), 8.03 (s, 1H), 7.96 (d, J = 7.2 Hz, 2H), 7.79 (s, 1H), 7.64-7.60 (m, 3H), 6.84 (d, J = 3.3 Hz, 1H), 6.60-6.59 (m, 1H), 3.08-3.05 (m, 2H), 2.27 (t, J = 7.4 Hz, 2H), 1.93-1.86 (m, 2H). ^{13}C NMR (126 MHz, DMSO) δ: (**first and second set**) 200.2, 200.0, 174.3, 168.7,

149.9, 149.7, 145.3, 145.1, 137.0, 136.2, 133.5, 133.1, 129.1, 128.3, 113.4, 113.3, 112.5, 112.4, 37.8, 37.6, 33.7, 31.5, 20.0, 19.1. LC-MS (m/z): 283 (M^{+}-1).

5-(4-chlorophenyl)-N'-((furan-2-yl)methylene)-5-oxopentanehydrazide (8b)

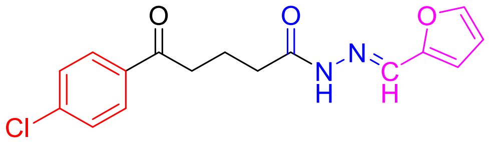

Light Brown powder, Yield 83 %, mp 132-133 °C, IR (ATR) υ_{max}/cm^{-1}: 3263 (NH), 3059 (CH, aryl), 2959 (CH, alkyl), 1654, 1623 (2C=O), 1394 (C-O), 1015 (N-N), 935, 741. Two sets of signals observed in NMR experiments. ^{1}H NMR (500 MHz, DMSO) δ: (**first set**) 11.20 (brs, 1H), 7.96 (d, *J* = 8.6 Hz, 2H), 7.84 (s, 1H), 7.75 (s, 1H), 7.56 (d, *J* = 8.5 Hz, 2H), 6.79 (d, *J* = 3.3 Hz, 1H), 6.57-7-6.56 (m, 1H), 3.10-3.04 (m, 2H), 2.63 (t, *J* = 7.4 Hz, 2H), 1.90-1.85 (m, 2H). ^{1}H NMR (500 MHz, DMSO) δ: (**second set**) 11.20 (brs, 1H), 8.03 (s, 1H), 7.96 (d, *J* = 8.6 Hz, 2H), 7.79 (s, 1H), 6.84 (d, *J* = 3.3 Hz, 1H), 6.59-6.58 (m, 1H), 3.10-3.04 (m, 2H), 2.26 (t, *J* = 7.3 Hz, 2H), 1.90-1.85 (m, 2H). ^{13}C NMR (126 MHz, DMSO) δ: (**first and second set**) 199.2, 199.0, 174.3, 168.7, 149.9, 149.7, 145.3, 145.1, 138.3, 136.2, 135.7, 133.2, 130.2, 129.2, 113.4, 113.3, 112.5, 112.4, 37.8, 37.6, 33.6, 31.4, 19.9, 19.0. LC-MS (m/z): 317 (M^{+}-1).

N'-((furan-2-yl)methylene)-5-oxo-5-p-tolylpentanehydrazide (8c)

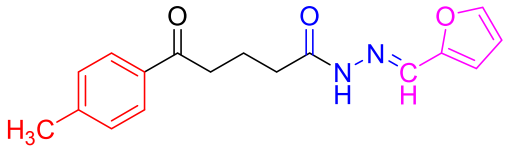

White powder, Yield 93 %, mp 133.5-134.5 °C, IR (ATR) υ_{max}/cm^{-1}: 3175 (NH), 3086 (CH, aryl), 2948 (CH, alkyl), 1676, 1654 (2C=O), 1397 (C-O), 1018 (N-N), 805, 747. Two sets of signals observed in NMR experiments. ^{1}H NMR (500 MHz, DMSO) δ: (**first set**) 11.19 (s, 1H), 7.87-7.85 (m, 2H), 7.80 (s, 1H), 7.77 (s, 1H), 7.32 (t, *J* = 7.1 Hz, 2H), 6.81-6.80 (m, 1H), 6.59-6.58 (m, 1H), 3.07-3.01 (m, 2H), 2.64 (t, *J* = 7.4 Hz, 2H), 2.36 (s, 3H), 1.93-1.86 (m, 2H). ^{1}H NMR (500 MHz, DMSO) δ: (**second set**) 11.29 (s, 1H), 8.04 (s, 1H), 7.87-7.85 (m, 2H), 7.80 (s, 1H), 7.32 (t, *J* = 7.1 Hz, 2H), 6.81-6.80 (m, 1H), 6.61-6.60 (m,1H), 3.07-3.01 (m, 2H), 2.36 (s, 3H), 2.27 (t, *J* = 7.3 Hz, 1H), 1.93-1.86 (m, 2H). ^{13}C NMR (126 MHz, DMSO) δ: (**first and second set**) 199.7, 199.5, 174.3, 168.7, 149.9, 149.7, 145.3, 145.1, 143.8, 143.8, 136.2, 134.6, 133.1, 129.6, 128.4, 113.4, 113.2, 112.5, 112.4, 37.7, 37.5, 33.7, 31.5, 21.5, 20.1, 19.2. LC-MS (m/z): 297 (M^{+}-1).

N'-((furan-2-yl)methylene)-5-(4-methoxyphenyl)-5-oxopentanehydrazide (8d)

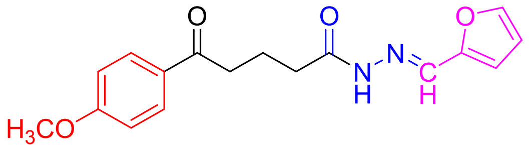

White powder, Yield 95 %, mp 144-145 ºC, IR (ATR) υ_{max}/cm^{-1}: 3172 (NH), 3086 (CH, aryl), 2951 (CH, alkyl), 1659, 1598 (2C=O), 1504 (C=N), 1397 (C-O), 1021 (N-N), 810, 747. Two sets of signals observed in NMR experiments. ^{1}H NMR (500 MHz, DMSO) δ: (**first set**) 11.23 (brs, 1H), 7.95 (d, *J* = 8.9 Hz, 2H), 7.85 (s, 1H), 7.77 (s, 1H), 7.02 (d, *J* = 8.9 Hz, 2H), 6.81 (d, *J* = 3.4 Hz, 1H), 6.59-7-6.58 (m, 1H), 3.83 (s, 3H), 3.04-2.98 (m, 2H), 2.64 (t, *J* = 7.4 Hz, 1H), 1.91-1.85 (m, 2H). ^{1}H NMR (500 MHz, DMSO) δ: (**second set**) 11.23 (brs, 1H), 8.05 (s, 1H), 7.96 (d, *J* = 8.6 Hz, 2H), 7.80 (s, 1H), 7.04 (d, *J* = 6.6 Hz, 2H), 6.85 (d, *J* = 3.4 Hz, 1H), 6.61-6.60 (m, 1H), 3.10-3.04 (m, 2H), 2.27 (t, *J* = 7.3 Hz, 2H), 1.91-1.85 (m, 2H). ^{13}C NMR (126 MHz, DMSO) δ: (**first and second set**) 198.6, 198.4, 174.3, 168.7, 149.9, 149.74, 145.3, 145.1, 136.2, 133.1, 130.6, 130.0, 114.2, 113.4, 113.2, 112.5, 112.4, 55.9, 37.5, 37.2, 33.7, 31.5, 20.2, 19.3. LC-MS (m/z): 313 (M^+-1).

N'-((furan-2-yl)methylene)-5-oxo-5-(thiophen-2-yl)pentanehydrazide (8e)

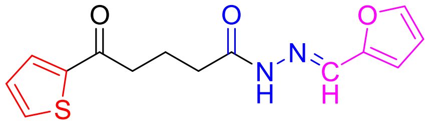

White powder, Yield 92 %, mp 121-122 ºC, IR (ATR) υ_{max}/cm^{-1}: 3236 (NH), 3064 (CH, aryl), 2934 (CH, alkyl), 1654, 1621 (2C=O), 1541 (C=N), 1408 (C-O), 1040 (N-N), 993, 716. Two sets of signals observed in NMR experiments. ^{1}H NMR (500 MHz, DMSO) δ: (**first set**) 11.23 (brs, 1H), 7.98 (t, *J* = 5.5 Hz, 1H), 7.95 (d, *J* = 3.8 Hz, 1H), 7.85 (s, 1H), 7.77 8S, 1H), 7.25-7.22 (m, 1H), 6.81 (d, *J* = 3.4 Hz, 1H), 6.59-6.58 (m, 1H), 3.04 (t, *J* = 7.3 Hz, 2H), 2.64 (t, *J* = 7.4 Hz, 2H), 1.93-1.88 (m, 2H). ^{1}H NMR (500 MHz, DMSO) δ: (**second set**) 11.23 (brs, 1H), 8.04 (s, 1H), 7.98 (t, *J* = 5.5 Hz, 1H), 7.95 (d, *J* = 3.8 Hz, 1H), 7.80 (s, 1H), 7.25-7.22 (m, 1H), 6.85 (d, *J* = 3.4 Hz, 1H), 6.61-6.60 (m, 1H), 2.27 (t, *J* = 7.4 Hz, 2H), 1.93-1.88 (m, 2H). ^{13}C NMR (126 MHz, DMSO) δ: (**first and second set**) 193.3, 193.2, 174.2, 168.6, 149.9, 149.7, 145.3, 145.1, 144.2, 136.2, 135.1, 135.0, 133.6, 133.5, 133.2, 129.1, 113.4, 113.3, 112.5, 112.4, 38.3, 38.0, 33.6, 31.4, 20.3, 19.4. LC-MS (m/z): 289 (M^+-1).

N'-(3-methylbutylidene)-5-oxo-5-phenylpentanehydrazide (9a)

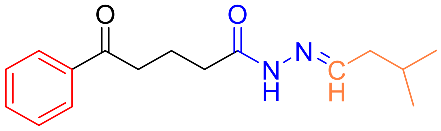

Light yellow oily, Yield 63 %, IR (ATR) υ_{max}/cm^{-1}: 3200 (NH), 3064 (CH, aryl), 2954 (CH, alkyl), 1651 (2C=O), 1551 (C=N), 1408 (C-O), 1051 (N-N), 849, 711. ^{1}H NMR (500 MHz, $CDCl_3$) δ: 9.05 (brs, 1H), 7.98 (d, J = 7.1 Hz, 1H), 7.47-7.44 (m, 2H), 7.34-7.32 (m, 2H), 7.09 (t, J = 5.7 Hz, 1H), 3.10 (t, J = 7.3 Hz, 2H), 2.76 (t, J = 7.2 Hz, 2H), 2.63-2.60 (m, 2H), 2.13-2.10 (m, 2H), 2.00-1.96 (m, 2H), 1.90-1.83 (m, 1H), 0.94 (d, J = 6.7 Hz, 6H). ^{13}C NMR (126 MHz, $CDCl_3$) δ: 199.7, 175.2, 146.3, 142.5, 132.9, 128.5, 128.0, 127.7, 125.6, 110.2, 64.5, 40.8, 39.9, 37.8, 32.4, 31.8, 26.5, 22.3, 18.8. LC-MS (m/z): 275 (M^{+}+1).

5-(4-chlorophenyl)-N'-(3-methylbutylidene)-5-oxopentanehydrazide (9b)

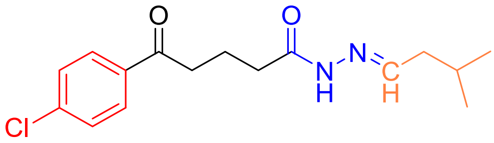

Light yellow oily, Yield 51 %, IR (ATR) υ_{max}/cm^{-1}: 3194 (NH), 3056 (CH, aryl), 2951 (CH, alkyl), 1657 (2C=O), 1554 (C=N), 1452 (C-O), 1087 (N-N), 1007, 832. ^{1}H NMR (500 MHz, $CDCl_3$) δ: 8.98 (brs, 1H), 7.39 (d, J = 8.5 Hz, 2H), 7.30 (d, J = 8.4 Hz, 2H), 7.09 (t, J = 5.7 Hz, 1H), 2.60 (t, J = 7.6 Hz, 2H), 2.13 – 2.10 (m, 2H), 1.96-1.93 (m, 4H), 1.88-1.83 (m, 1H), 0.94 (d, J = 6.7 Hz, 6H). ^{13}C NMR (126 MHz, $CDCl_3$) δ: 198.5, 175.1, 146.4, 141.2, 133.6, 129.5, 128.8, 128.2, 127.2, 109.8, 64.5, 40.8, 39.8, 32.4, 26.5, 22.3, 18.8. LC-MS (m/z): 309 (M^{+}+1).

N'-(3-methylbutylidene)-5-oxo-5-p-tolylpentanehydrazide (9c)

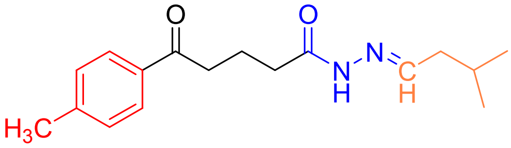

Light yellow oily, Yield 67 %, IR (ATR) υ_{max}/cm^{-1}: 3216 (NH), 3064 (CH, aryl), 2951 (CH, alkyl), 1665 (2C=O), 1546 (C=N), 1449 (C-O), 1178 (N-N), 968, 813. ^{1}H NMR (500 MHz, $CDCl_3$) δ: 9.18 (brs, 1H), 7.88 (d, J = 8.2 Hz, 2H), 7.25 (d, J = 7.9 Hz, 2H), 7.13 (t, J = 5.6 Hz, 1H), 3.06 (t, J = 7.3 Hz, 2H), 2.75 (t, J = 7.2 Hz, 2H), 2.41 (s, 3H), 2.12-2.10 (m, 4H), 1.88-1.83 (m, 1H), 0.94 (d, J = 6.7 Hz, 6H). ^{13}C NMR (126 MHz, $CDCl_3$) δ: 199.4, 175.0, 146.8, 143.6, 129.2, 128.1, 125.6, 64.4, 40.8, 37.7, 31.8, 26.5, 22.3, 21.6, 19.2. LC-MS (m/z): 289 (M^{+}+1).

5-(4-methoxyphenyl)-N'-(3-methylbutylidene)-5-oxopentanehydrazide (9d)

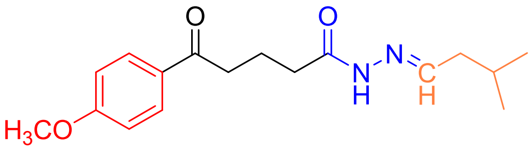

Light yellow oily, Yield 71 %, IR (ATR) υ_{max}/cm^{-1}: 3208 (NH), 3067 (CH, aryl), 2951 (CH, alkyl), 1657 (2C=O), 1596 (C=N), 1455 (C-O), 1164 (N-N), 821, 725. ^{1}H NMR (500 MHz, $CDCl_3$) δ: 9.17 (brs, 1H), 7.96 (d, *J* = 8.9 Hz, 2H), 7.14 (t, *J* = 5.7 Hz, 1H), 6.93 (d, *J* = 8.9 Hz, 2H), 3.87 (s, 3H), 3.04 (t, *J* = 7.3 Hz, 2H), 2.75 (t, *J* = 7.2 Hz, 2H), 2.12-2.10 (m, 4H), 1.90-1.91 (m, 1H), 0.94 (d, *J* = 6.7 Hz, 6H). ^{13}C NMR (126 MHz, $CDCl_3$) δ: 198.4, 175.0, 163.3, 146.7, 130.3, 130.0, 113.6, 55.4, 40.8, 37.5, 31.9, 26.5, 22.3, 19.3. LC-MS (m/z): 306 (M^++2).

N'-(3-methylbutylidene)-5-oxo-5-(thiophen-2-yl)pentanehydrazide (9e)

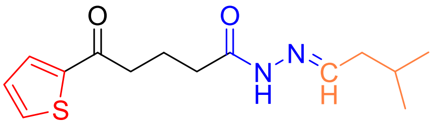

Light brown oily, Yield 69 %, IR (ATR) υ_{max}/cm^{-1}: 3200 (NH), 3064 (CH, aryl), 2954 (CH, alkyl), 1651 (2C=O), 1551 (C=N), 1408 (C-O), 1051 (N-N), 849, 711. ^{1}H NMR (500 MHz, $CDCl_3$) δ: 9.15 (brs, 1H), 7.23-7.21 (m, 1H), 7.14-7.10 (m, 1H), 7.03-7.02 (m, 1H), 6.96-6.94 (m, 1H), 3.03 (t, *J* = 7.3 Hz, 2H), 2.64 (t, *J* = 7.7 Hz, 2H), 2.14-2.09 (m, 4H), 2.05-2.02 (m, 2H), 0.95 (d, *J* = 6.7 Hz, 6H). ^{13}C NMR (126 MHz, $CDCl_3$) δ: 192.7, 175.2, 146.7, 133.3, 131.8, 128.0, 126.7, 124.9, 124.4, 108.9, 65.0, 53.4, 40.9, 40.1, 38.6, 32.4, 31.7, 26.5, 22.3, 18.9. LC-MS (m/z): 281 (M^++1).

References

(1) Küçük, H. B.; Alhonaish, A.; Yıldız, T.; Güzel, M. An Efficient Approach to Access 2,5-Disubstituted 1,3,4-Oxadiazoles by Oxidation of 2-Arenoxybenzaldehyde N-Acyl Hydrazones with Molecular Iodine. *ChemistrySelect* **2022,** *7,* e202201391.

(2) Aydın, E.; Şentürk, A. M.; Küçük, H. B.; Güzel, M. Cytotoxic Activity and Docking Studies of 2-Arenoxybenzaldehyde N-Acyl Hydrazone and 1,3,4-Oxadiazole Derivatives against Various Cancer Cell Lines. *Molecules* **2022**, *27* (21), 7309.

Copies of the IR, ^{1}H NMR and ^{13}C NMR spectra of synthesized compounds

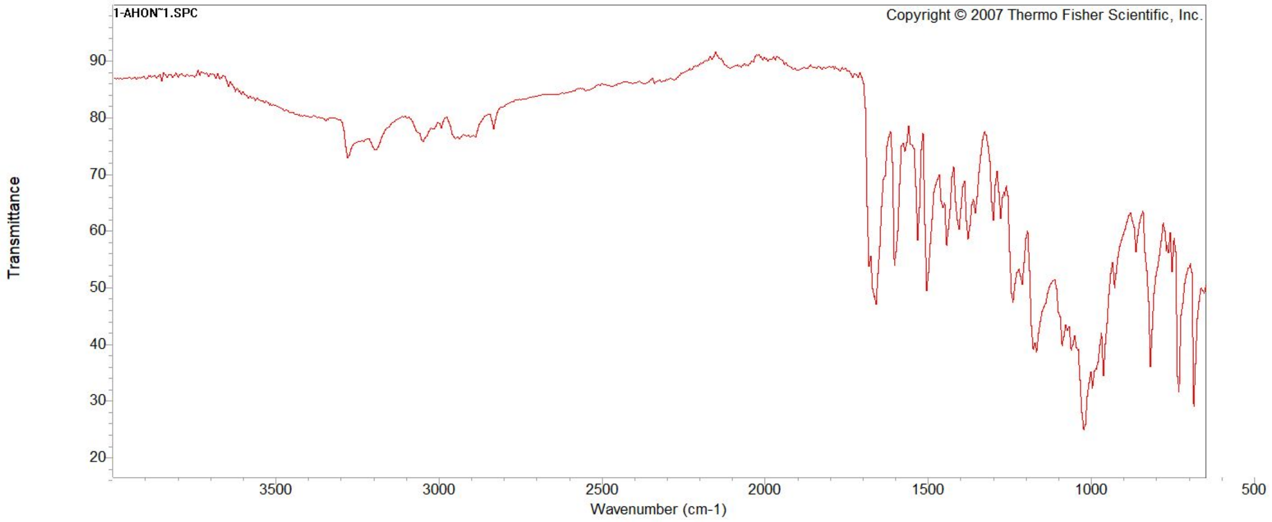

Figure S1. IR spectrum (ATR) of **7a**.

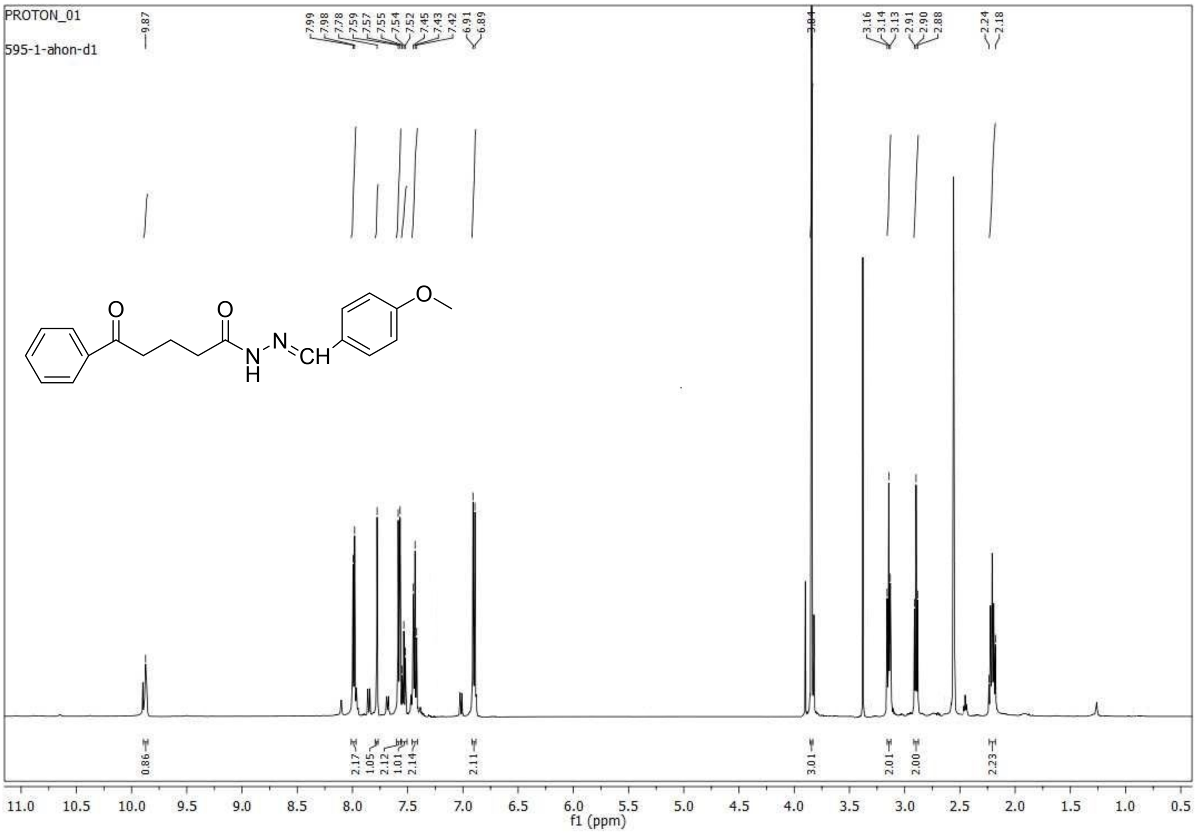

Figure S2. ^{1}H NMR spectrum (500 MHz, DMSO) of **7a**.

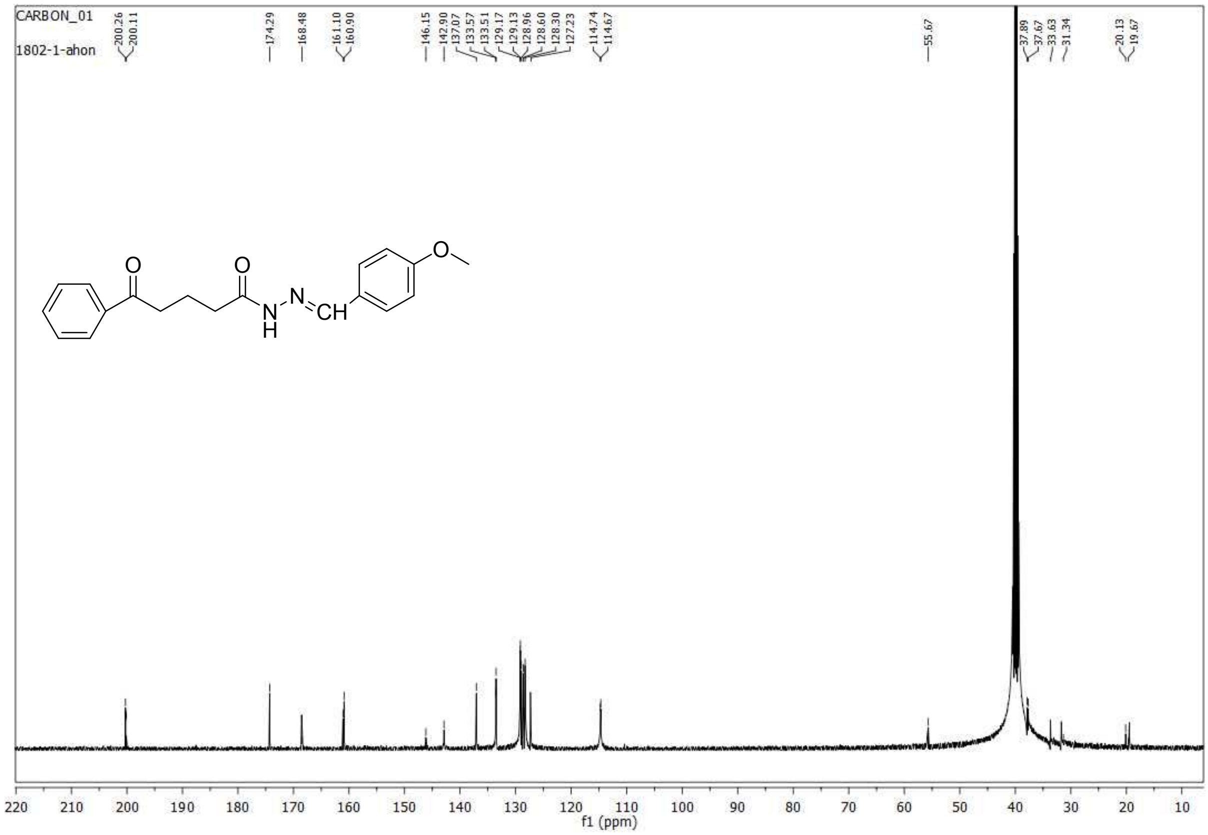

Figure S3. ^{13}C NMR spectrum (126 MHz, DMSO) of **7a**.

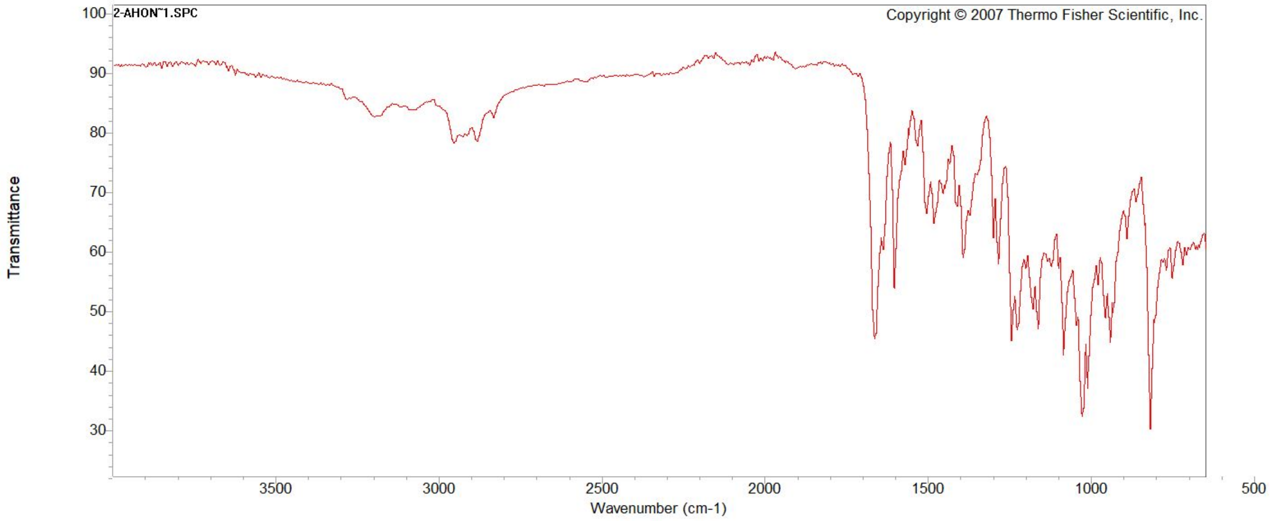

Figure S4. IR spectrum (ATR) of **7b**.

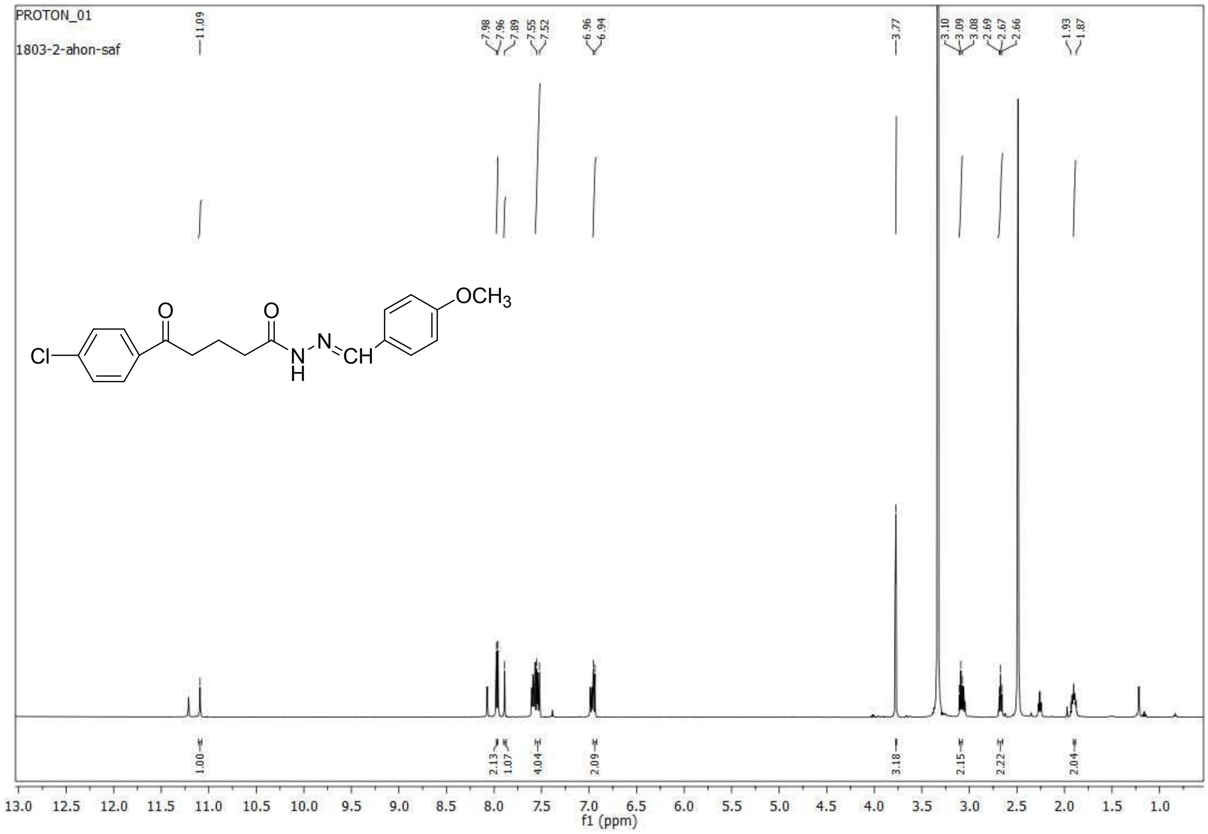

Figure S5. ^{1}H NMR spectrum (500 MHz, DMSO) of **7b**.

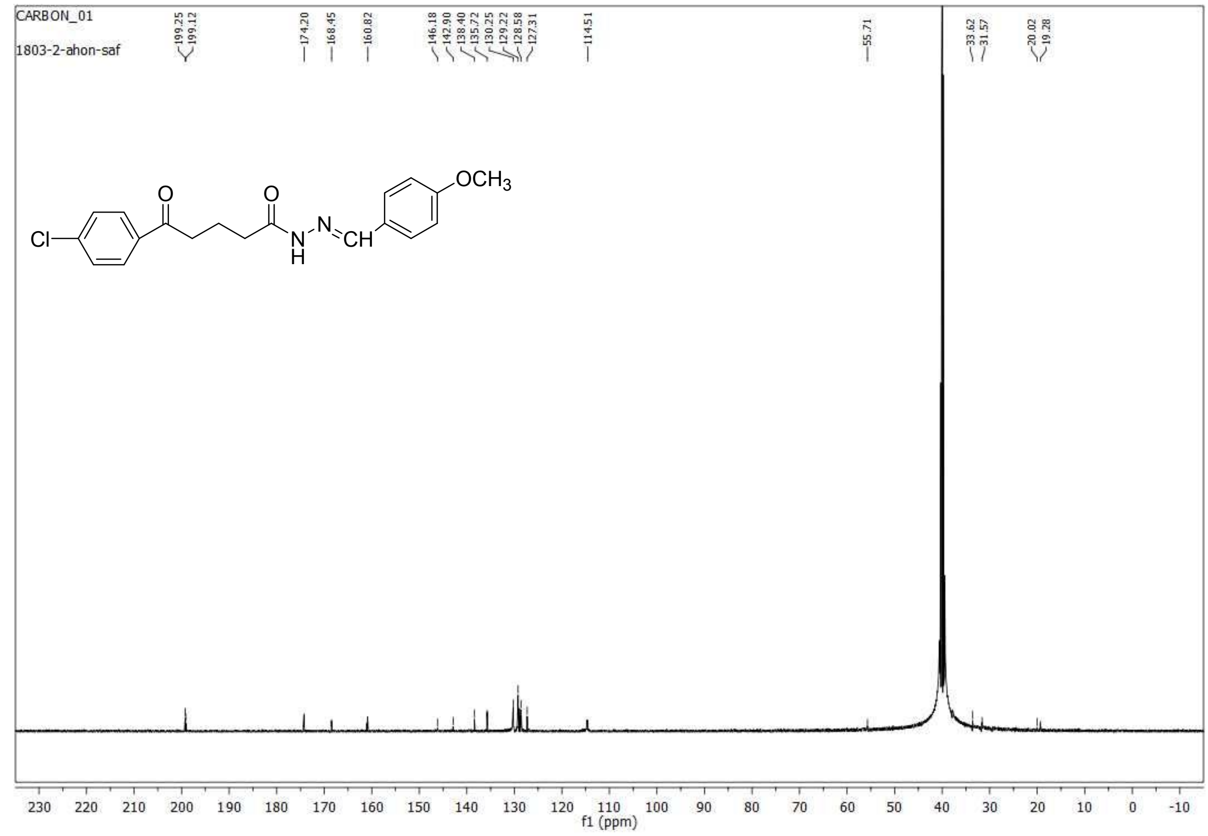

Figure S6. ^{13}C NMR spectrum (126 MHz, DMSO) of **7b**.

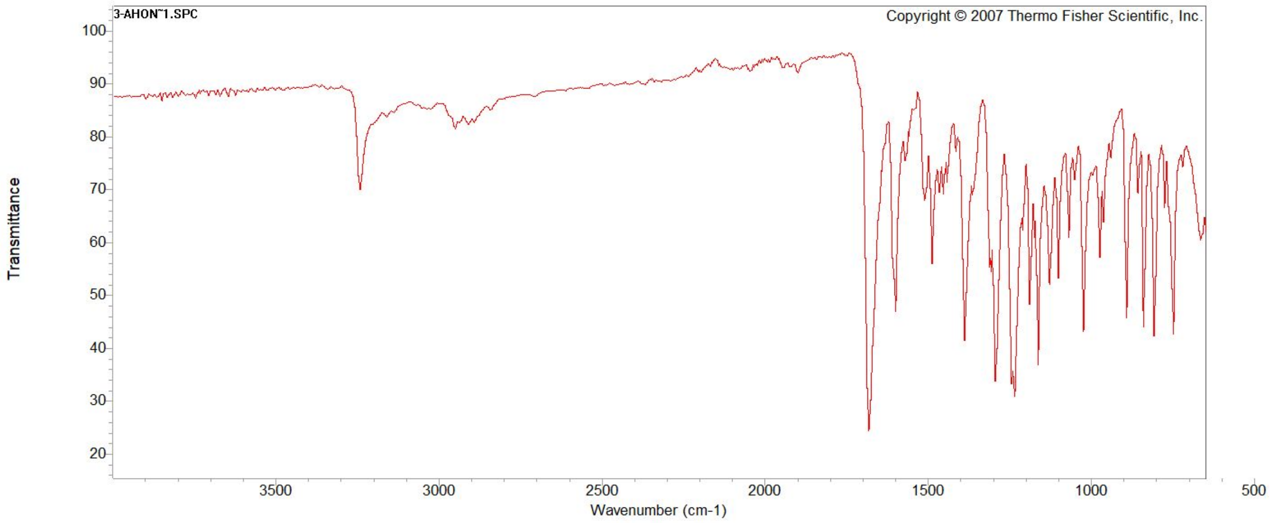

Figure S7. IR spectrum (ATR) of **7c**.

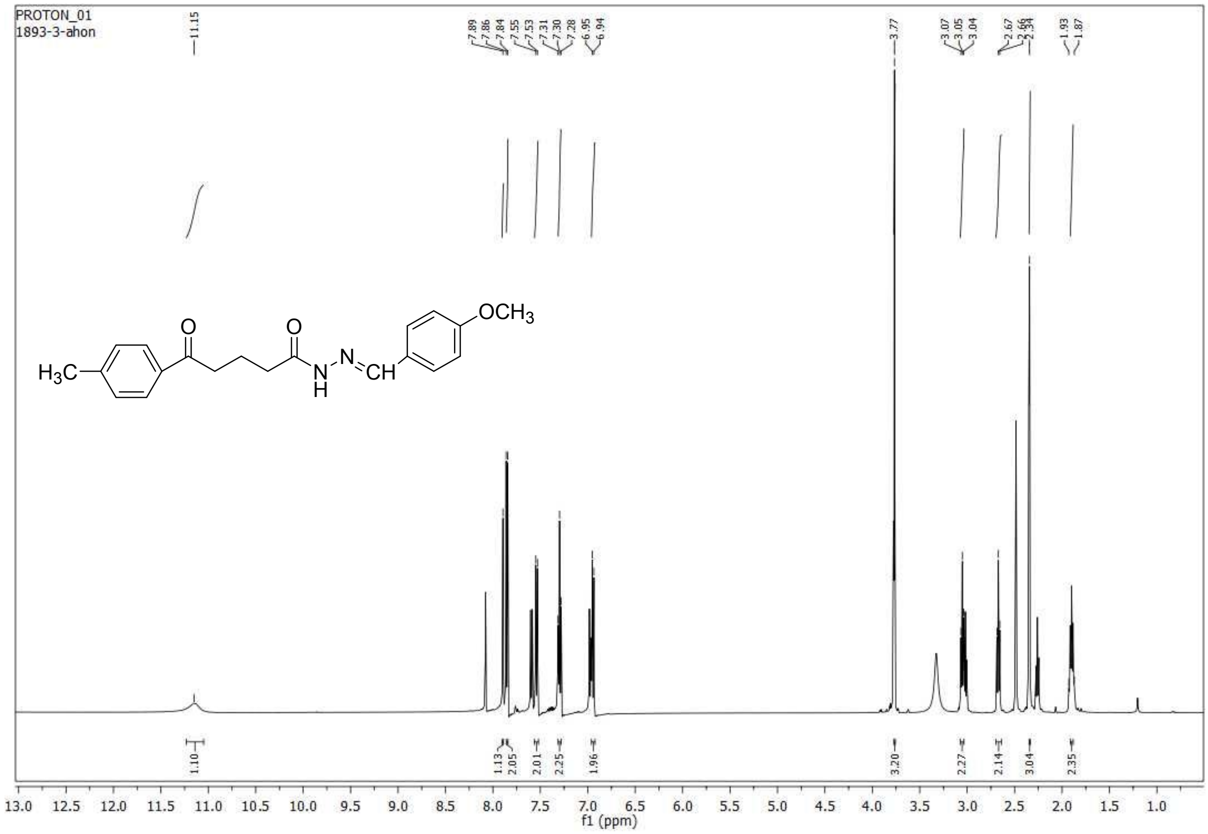

Figure S8. ^{1}H NMR spectrum (500 MHz, DMSO) of **7c**.

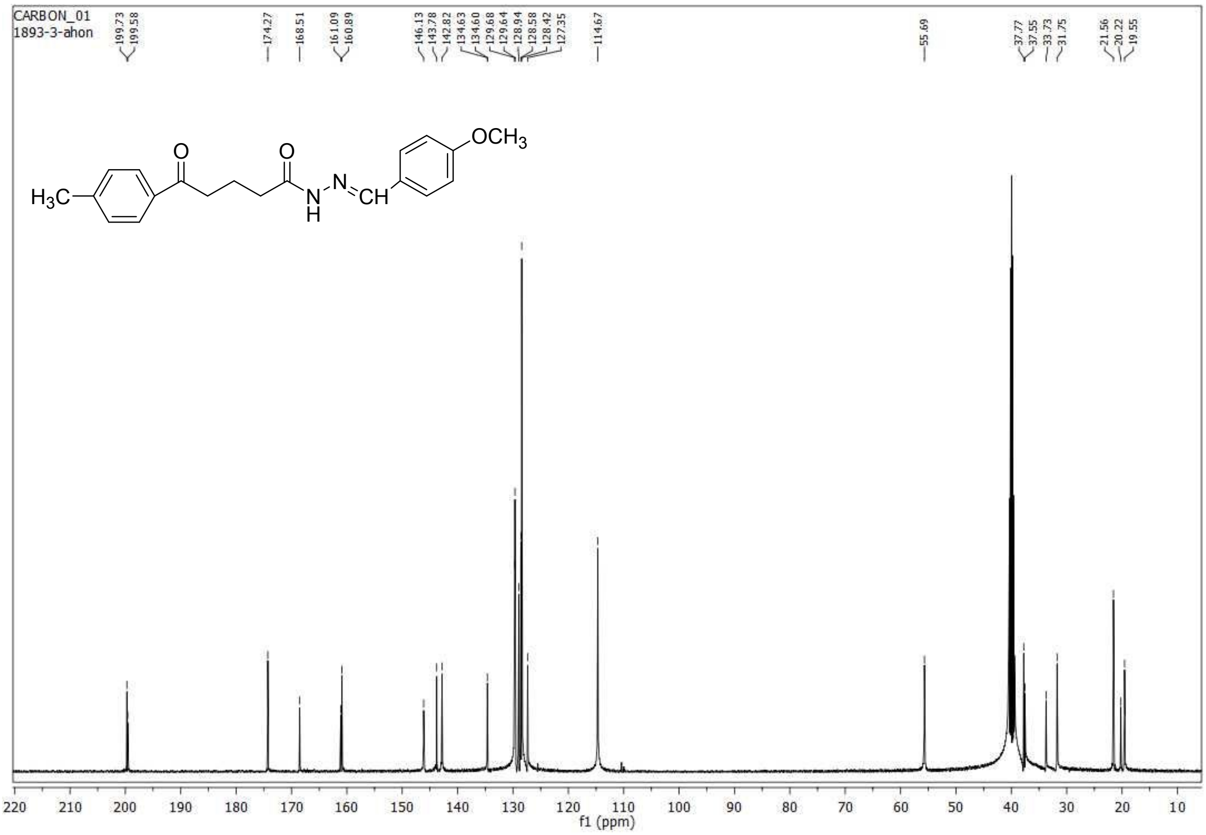

Figure S9. ^{13}C NMR spectrum (126MHz, DMSO) of **7c**.

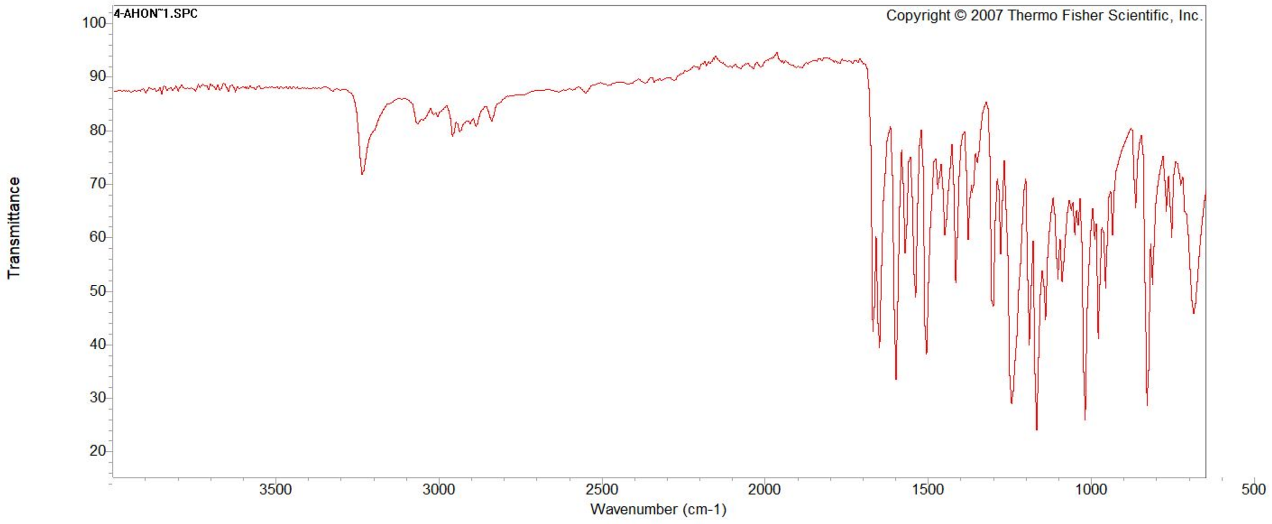

Figure S10. IR spectrum (ATR) of **7d**.

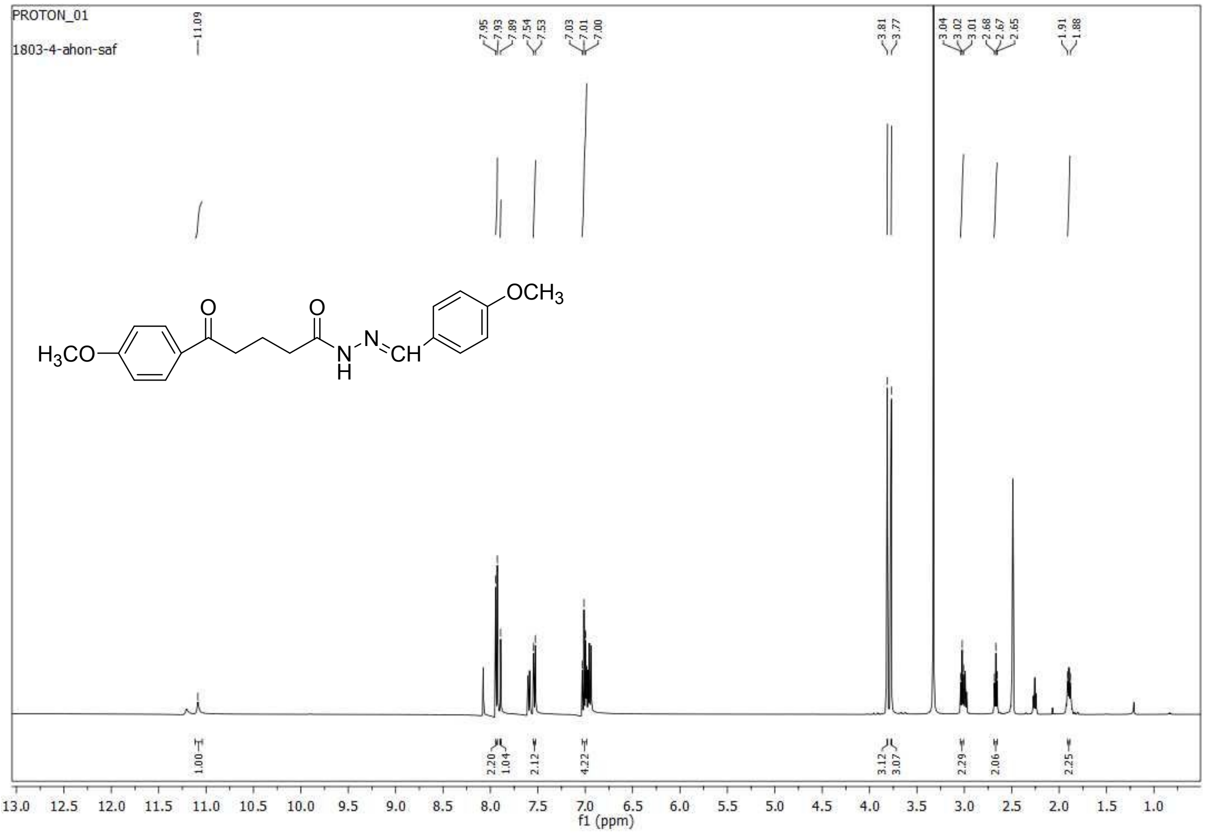

Figure S11. ^{1}H NMR spectrum (500 MHz, DMSO) of **7d**.

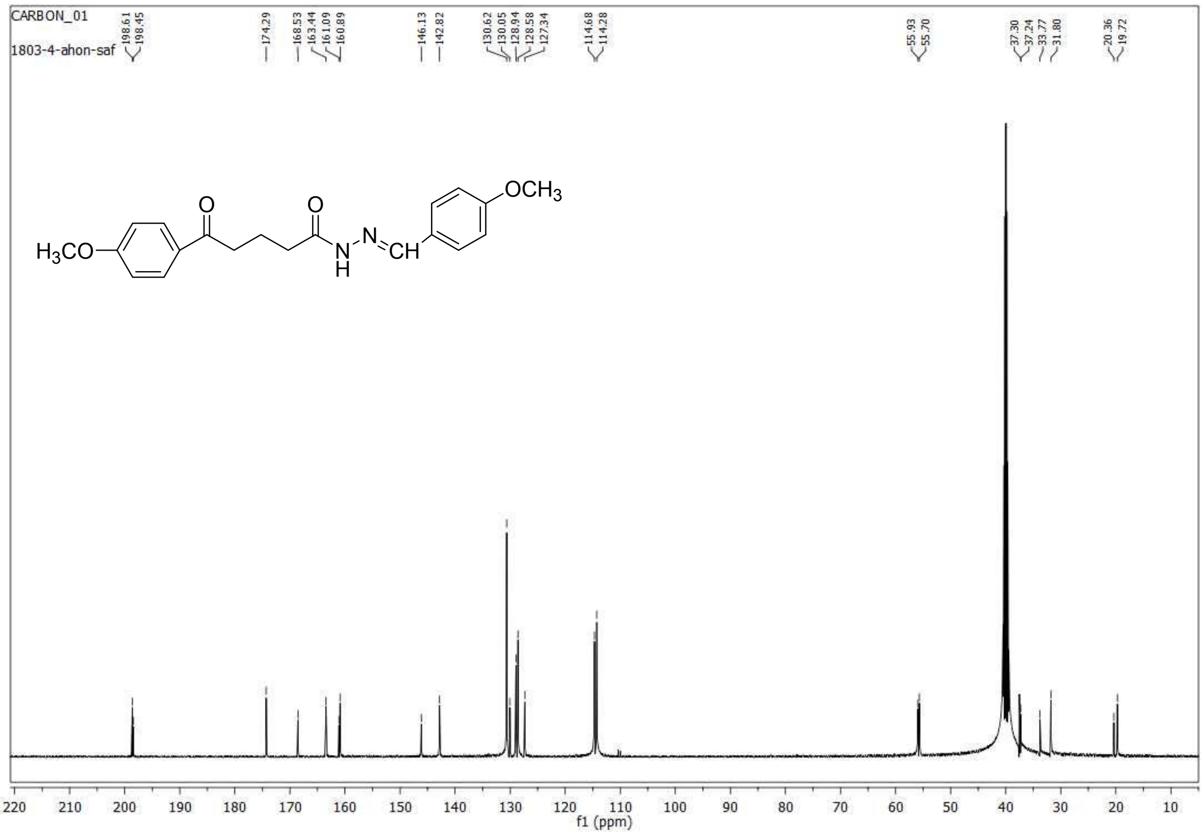

Figure S12. ^{13}C NMR spectrum (126 MHz, DMSO) of **7d**.

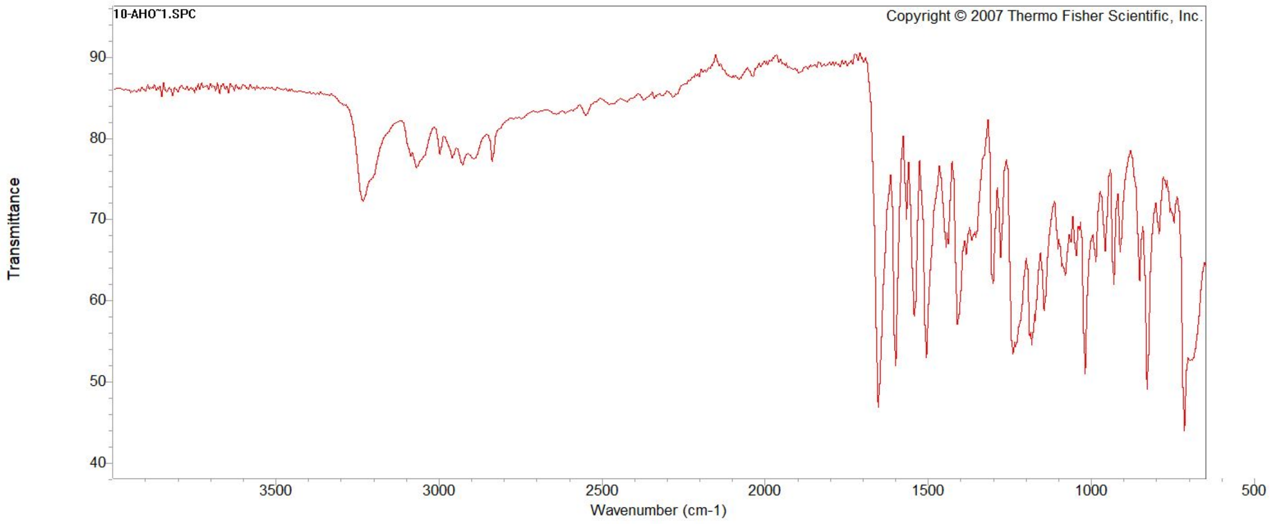

Figure S13. IR spectrum (ATR) of **7e**.

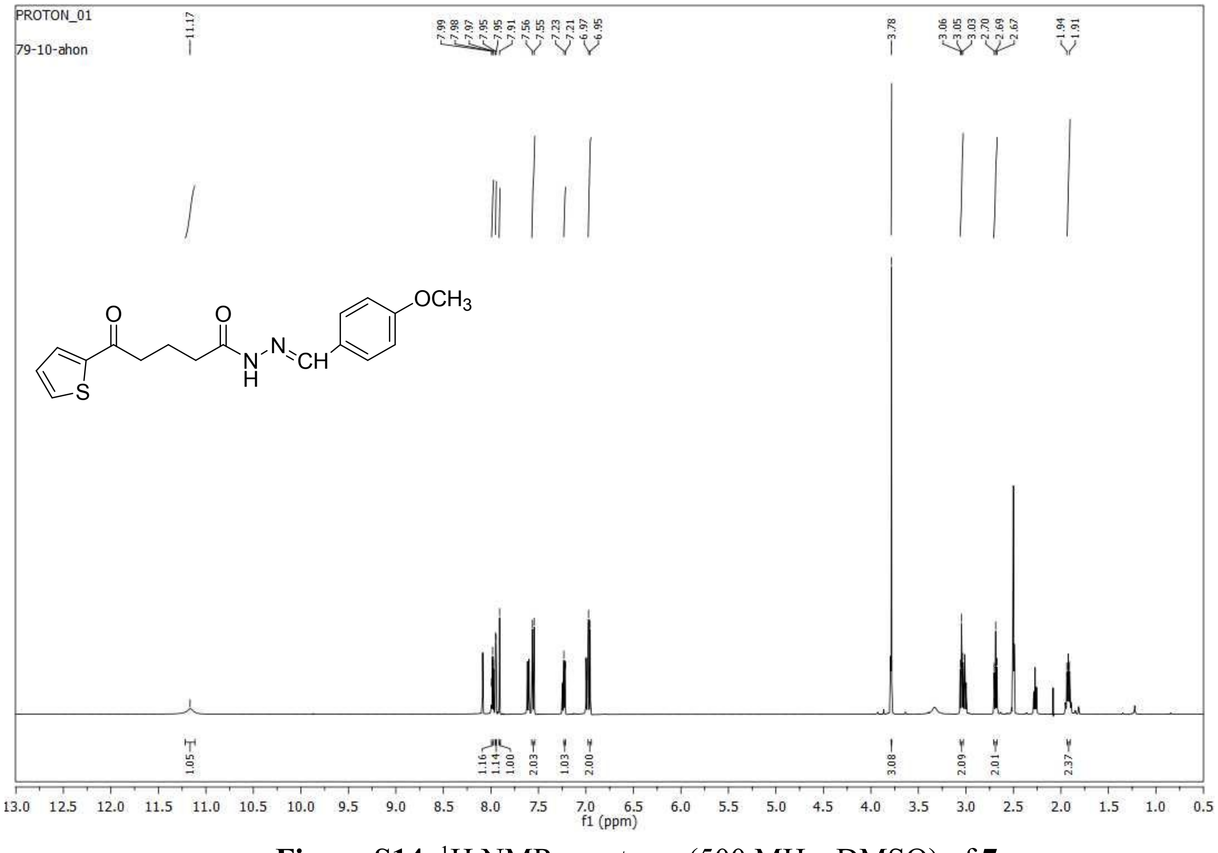

Figure S14. ^{1}H NMR spectrum (500 MHz, DMSO) of **7e**.

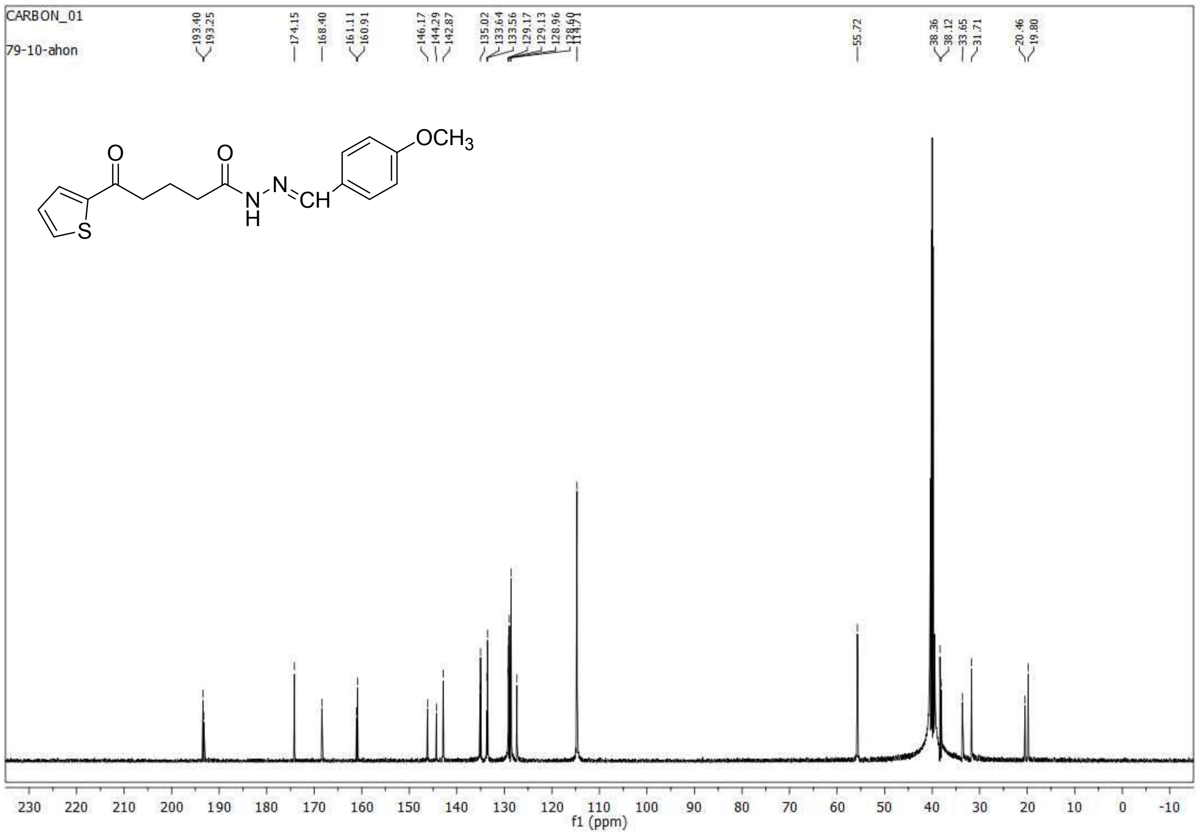

Figure S15. ^{13}C NMR spectrum (126 MHz, DMSO) of **7e**.

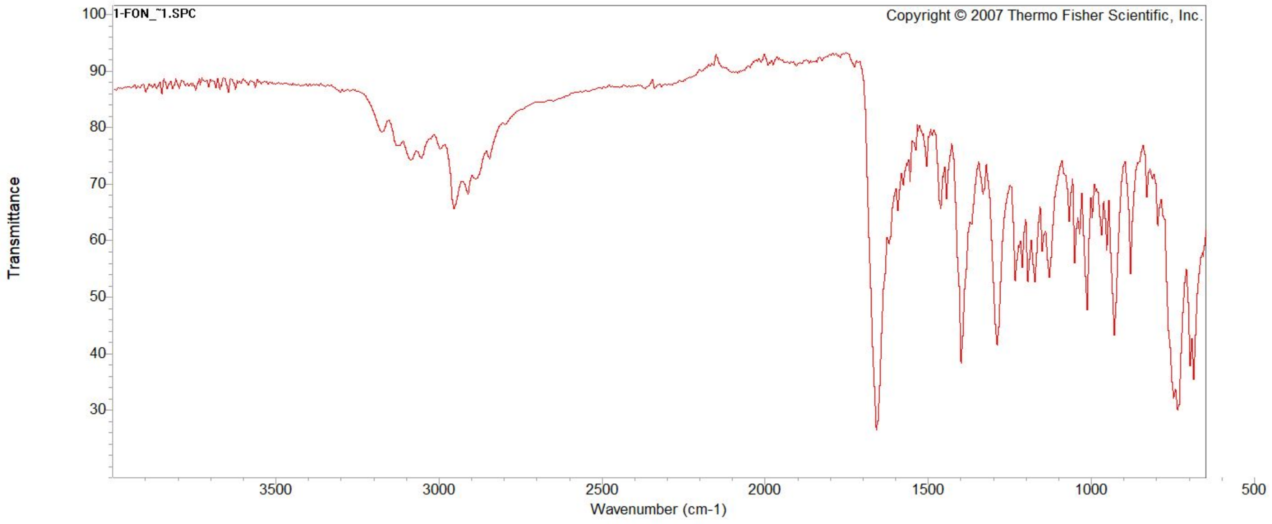

Figure S16. IR spectrum (ATR) of **8a**.

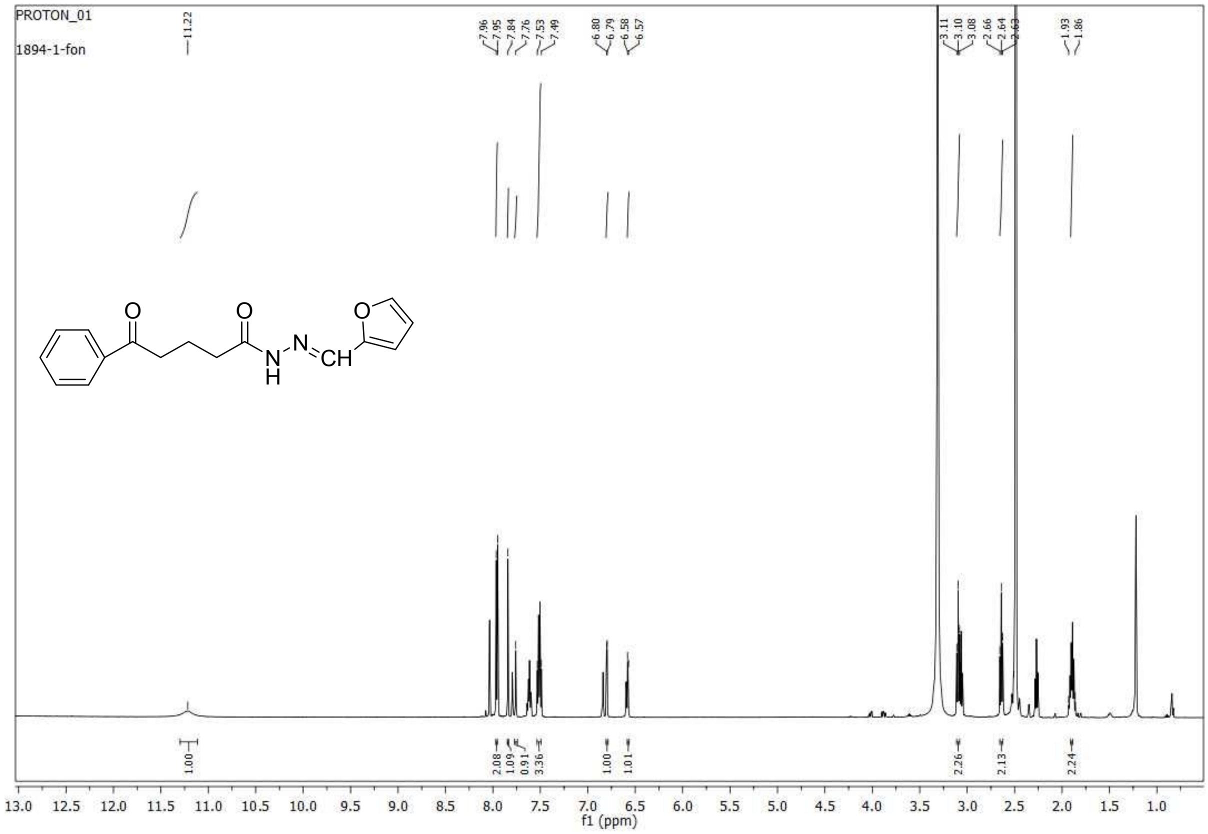

Figure S17. ^{1}H NMR spectrum (500 MHz, DMSO) of **8a**.

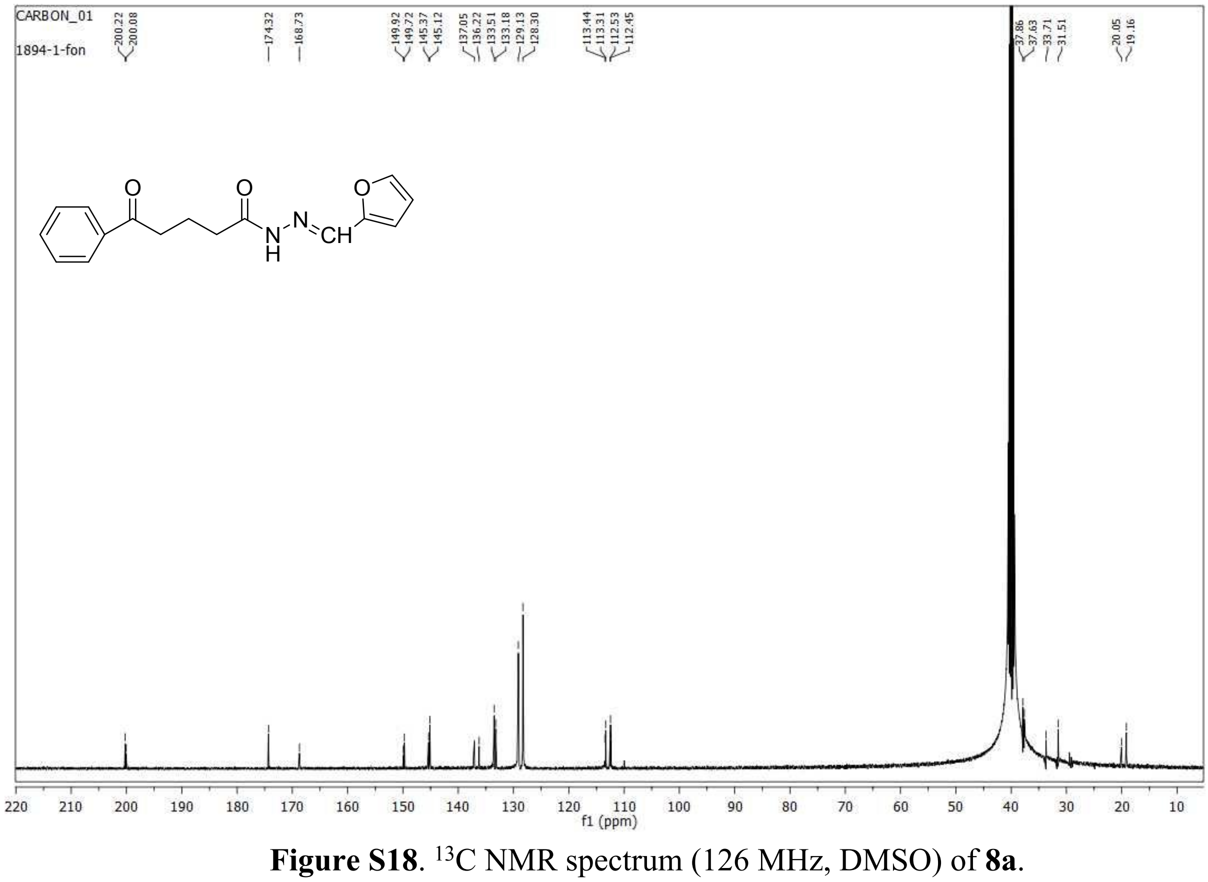

Figure S18. ^{13}C NMR spectrum (126 MHz, DMSO) of **8a**.

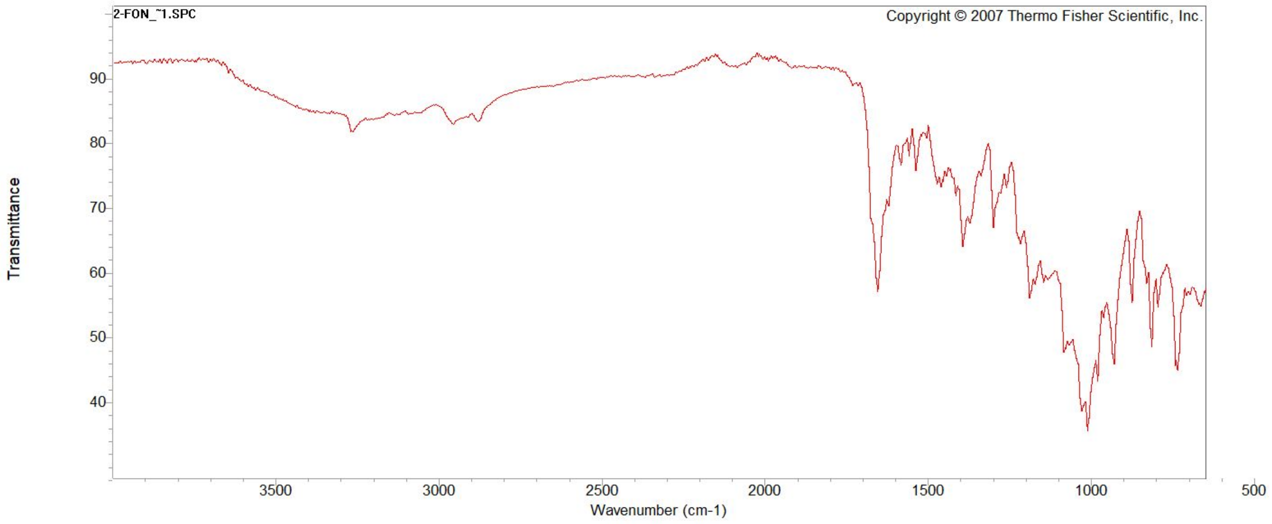

Figure S19. IR spectrum (ATR) of **8b**.

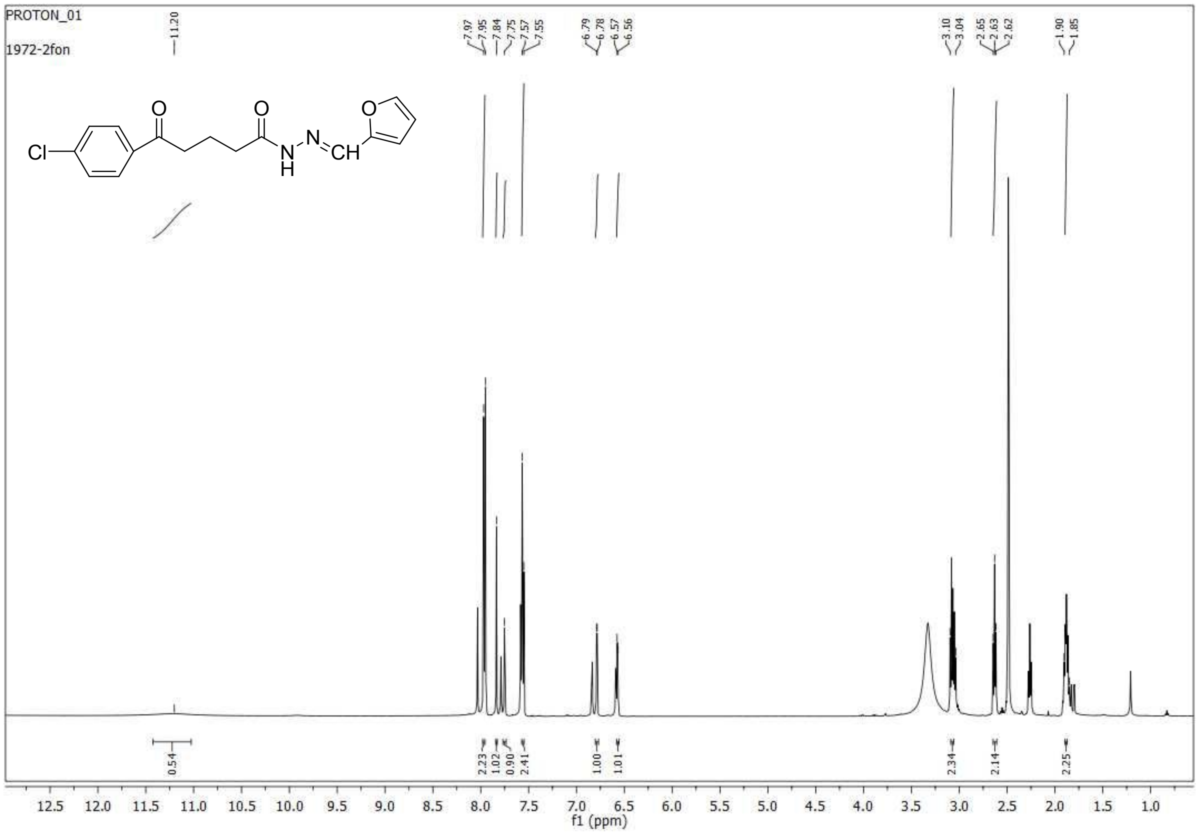

Figure S20. ^{1}H NMR spectrum (500 MHz, DMSO) of **8b**.

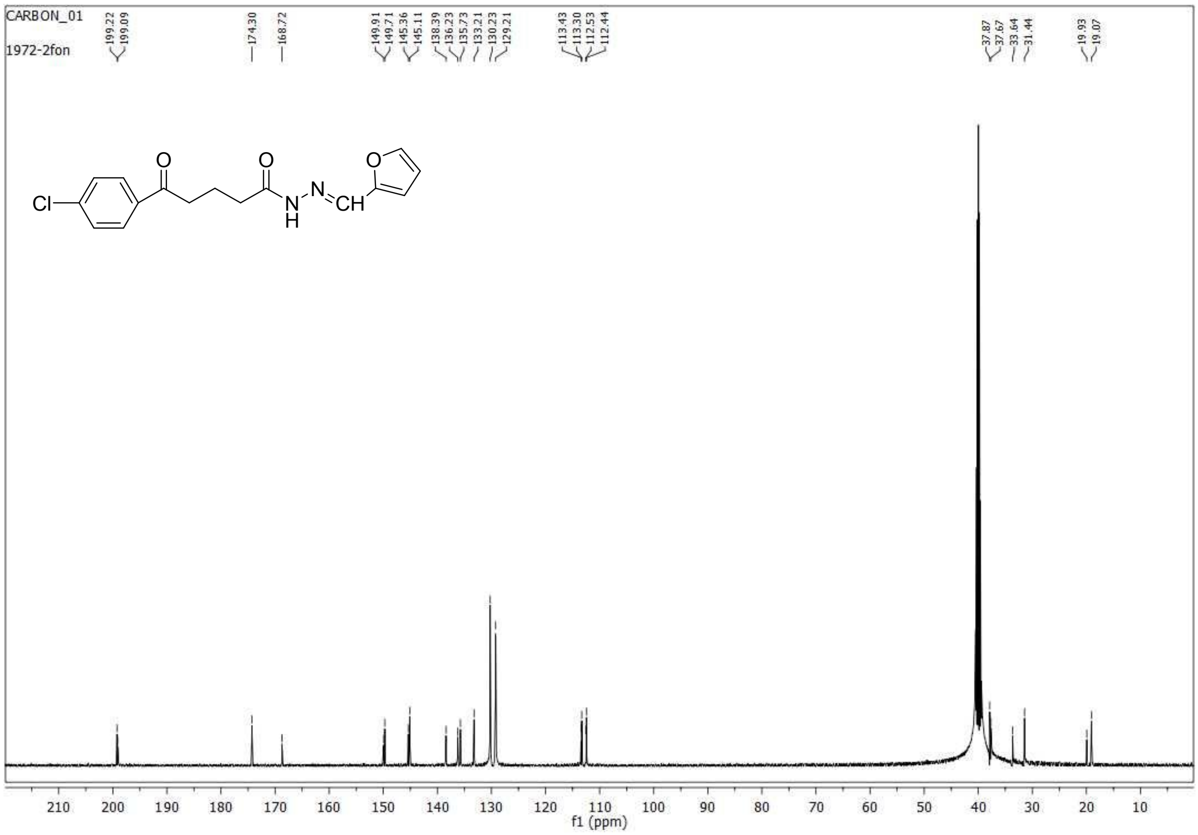

Figure S21. ^{13}C NMR spectrum (126 MHz, DMSO) of **8b**.

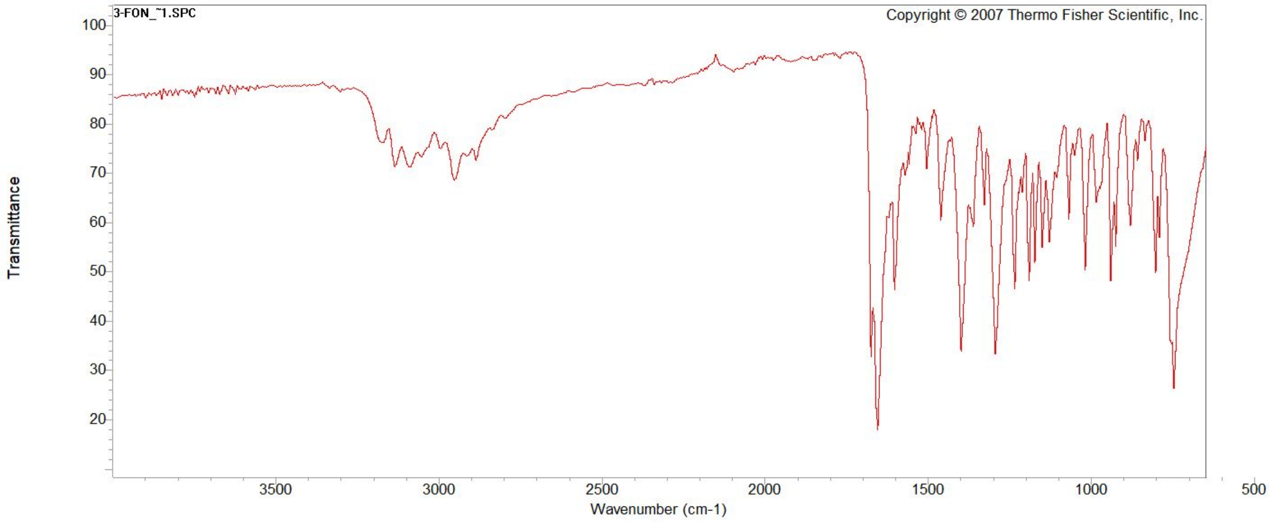

Figure S22. IR spectrum (ATR) of **8c**.

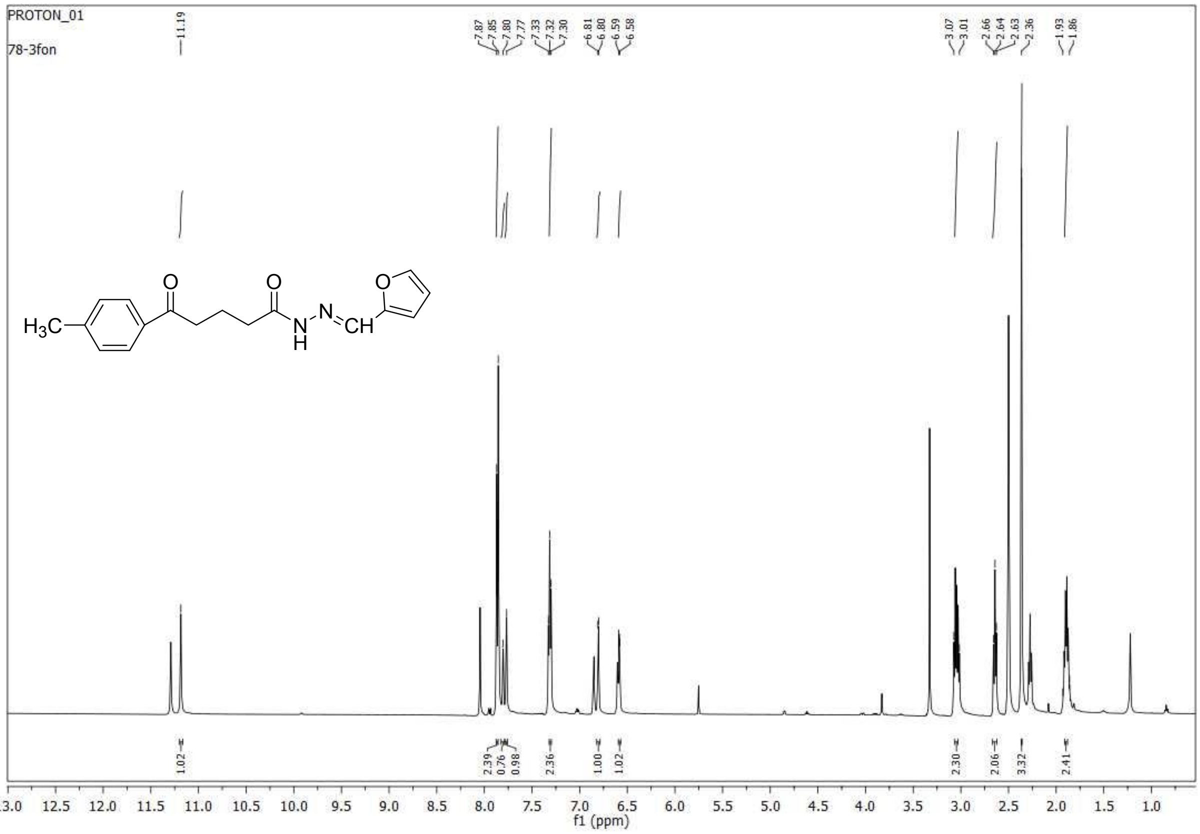

Figure S23. ^{1}H NMR spectrum (500 MHz, DMSO) of **8c**.

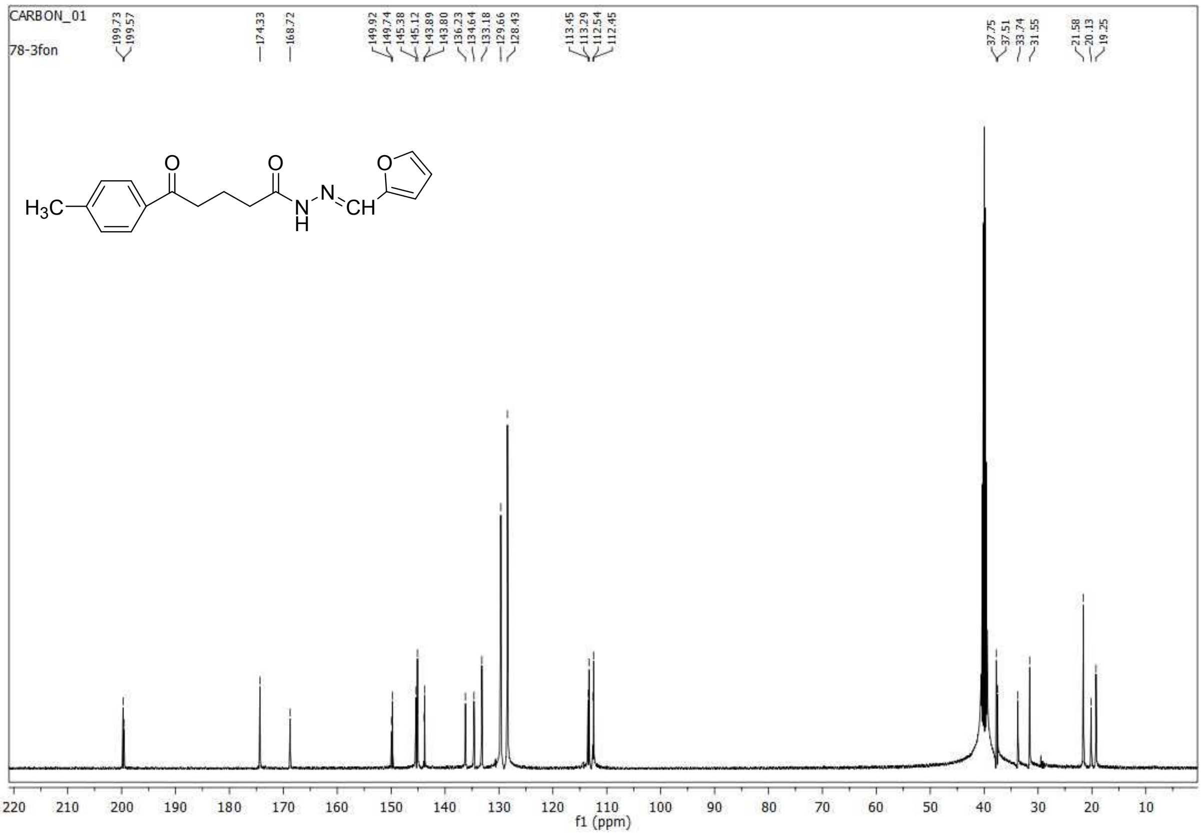

Figure S24. ^{13}C NMR spectrum (126 MHz, DMSO) of **8c**.

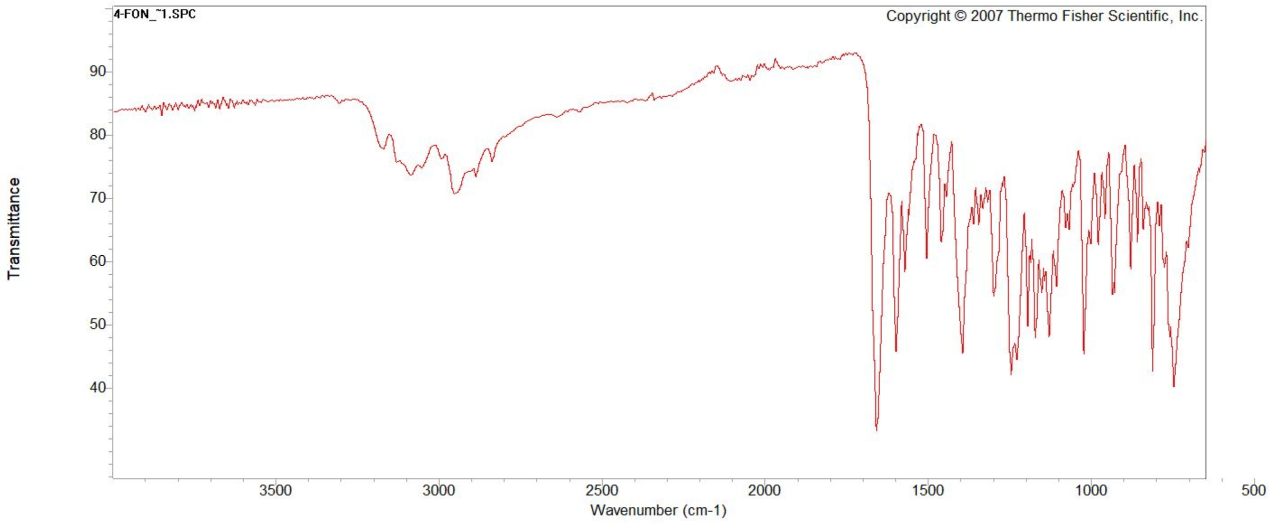

Figure S25. IR spectrum (ATR) of **8d**.

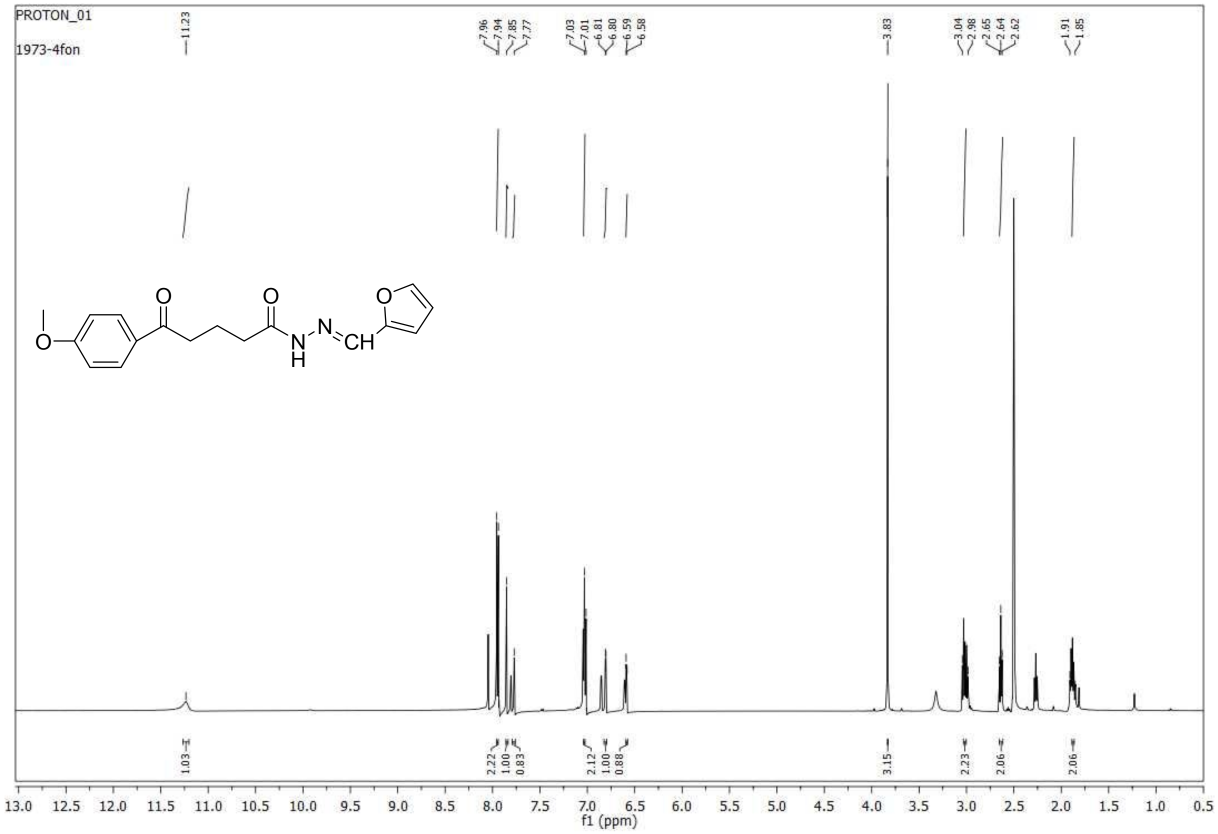

Figure S26. ^{1}H NMR spectrum (500 MHz, DMSO) of **8d**.

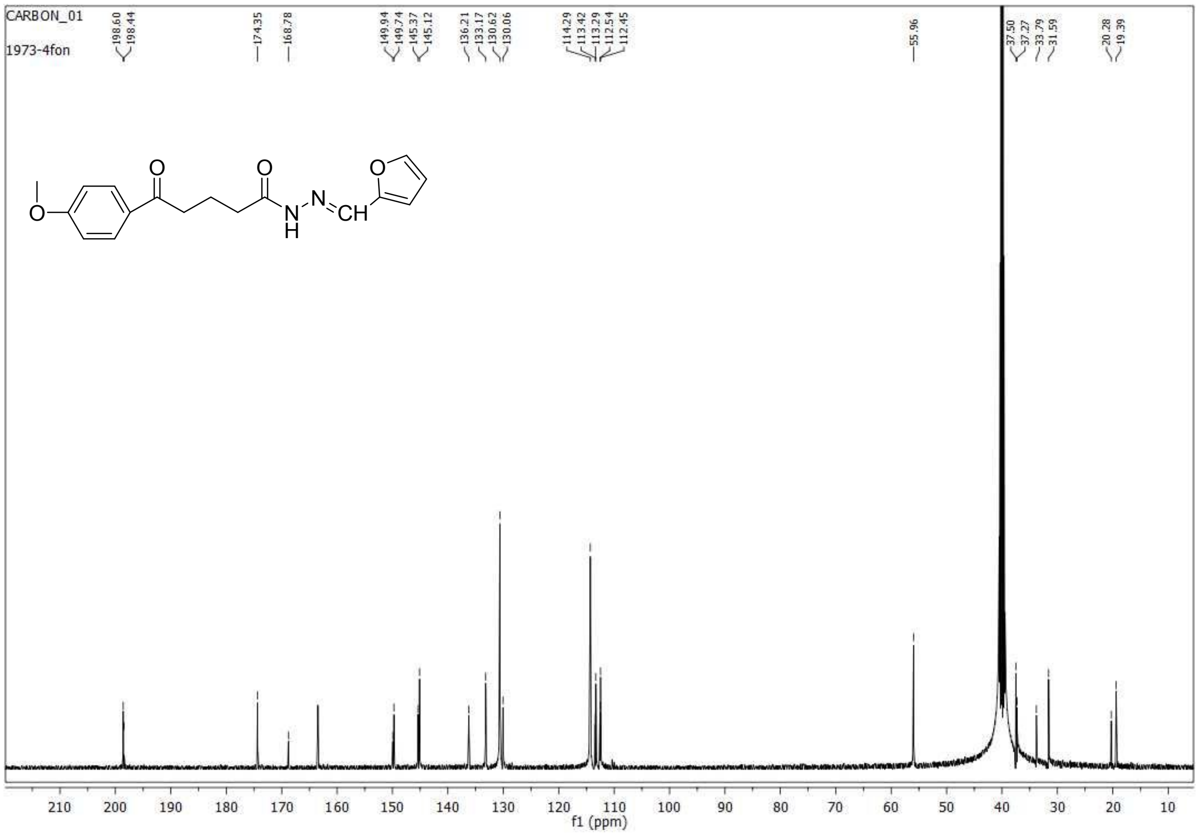

Figure S27. ^{13}C NMR spectrum (126 MHz, DMSO) of **8d**.

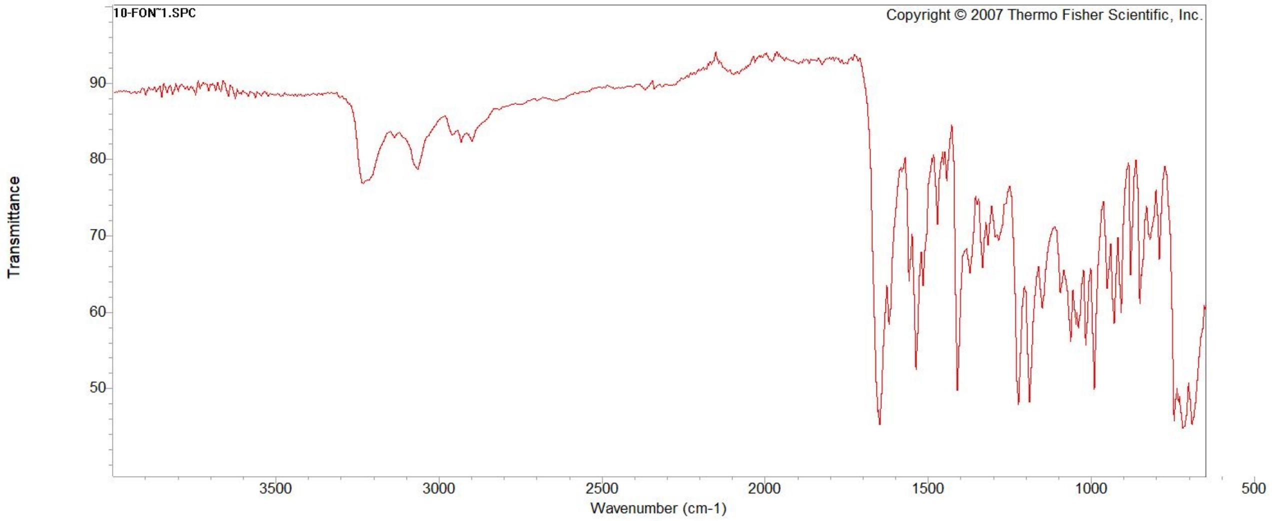

Figure S28. IR spectrum (ATR) of **8e**.

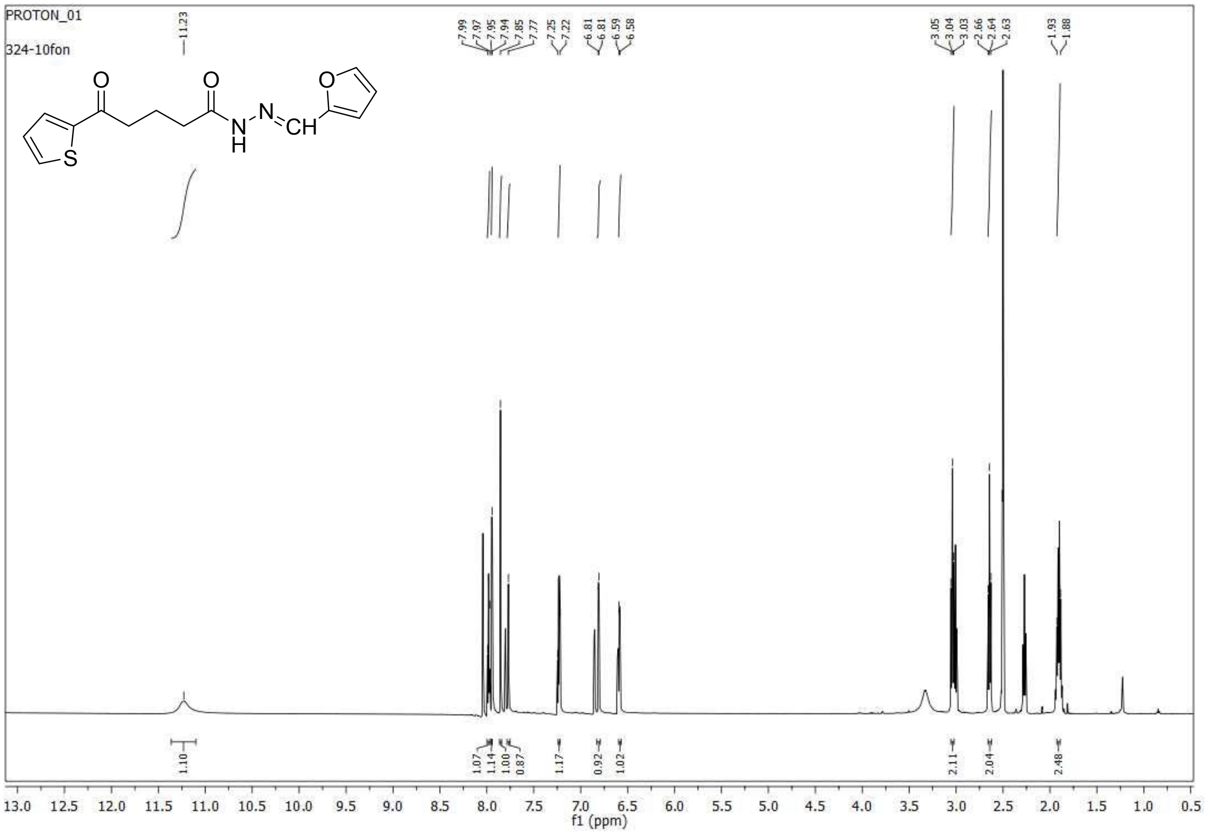

Figure S29. ^{1}H NMR spectrum (500 MHz, DMSO) of **8e**.

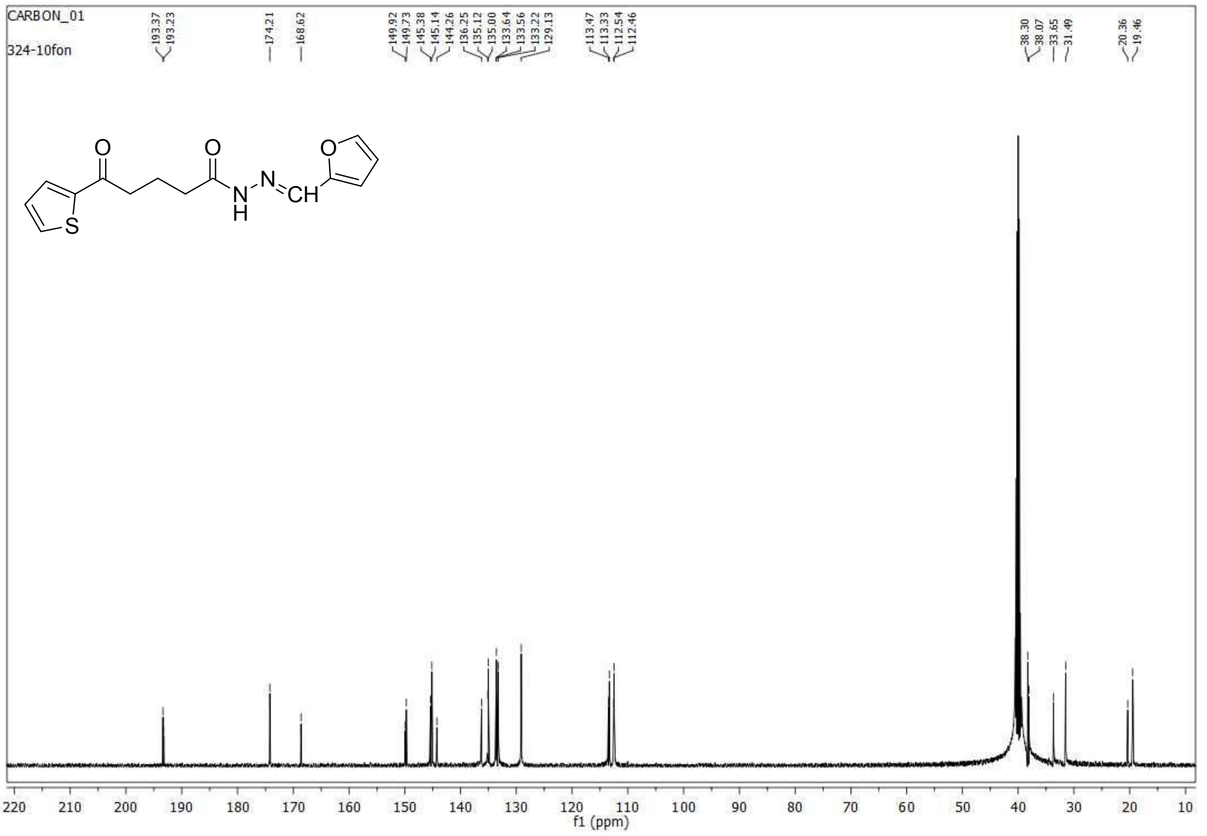

Figure S30. ^{13}C NMR spectrum (126 MHz, DMSO) of **8e**.

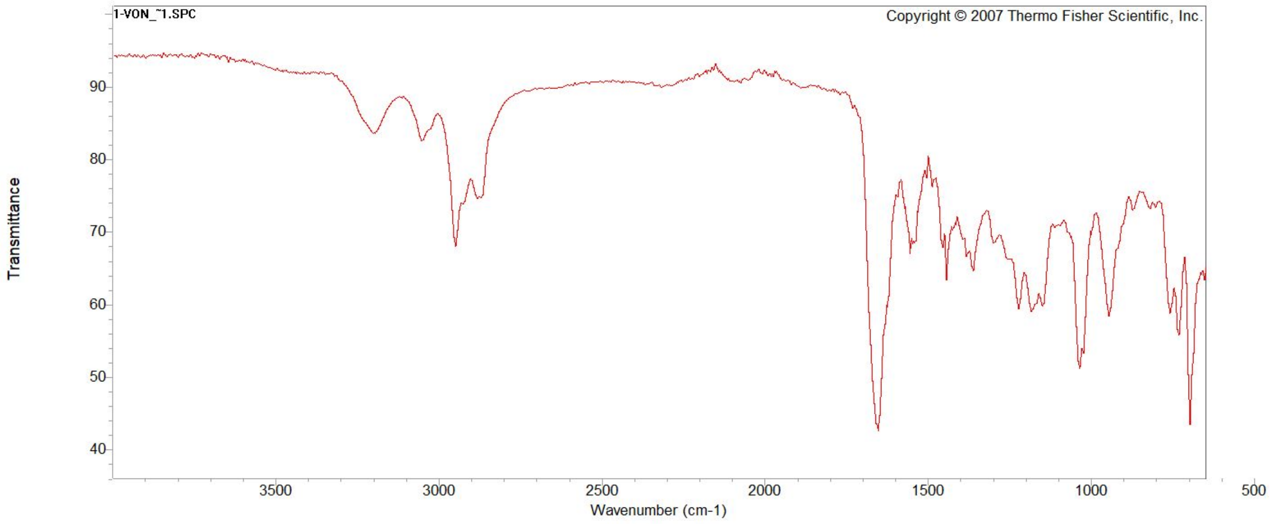

Figure S31. IR spectrum (ATR) of **9a**.

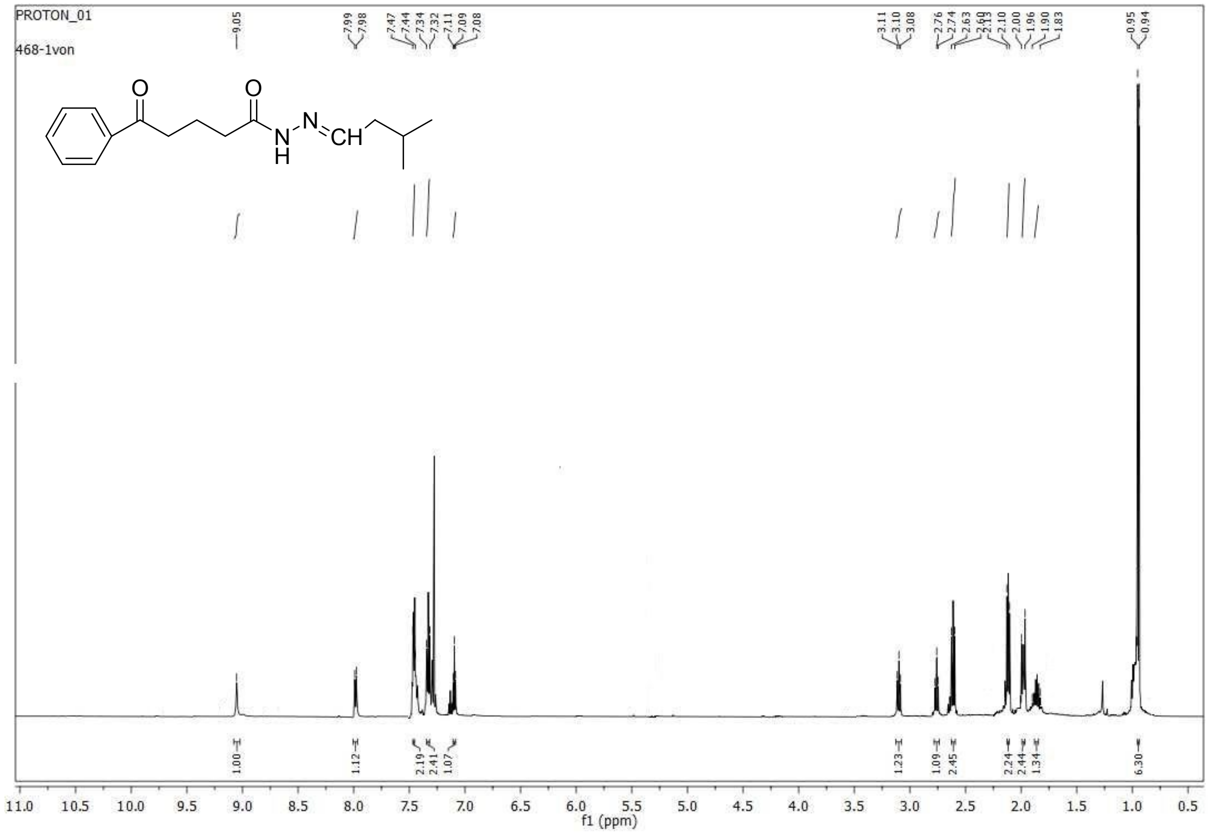

Figure S32. ^{1}H NMR spectrum (500 MHz, DMSO) of **9a**.

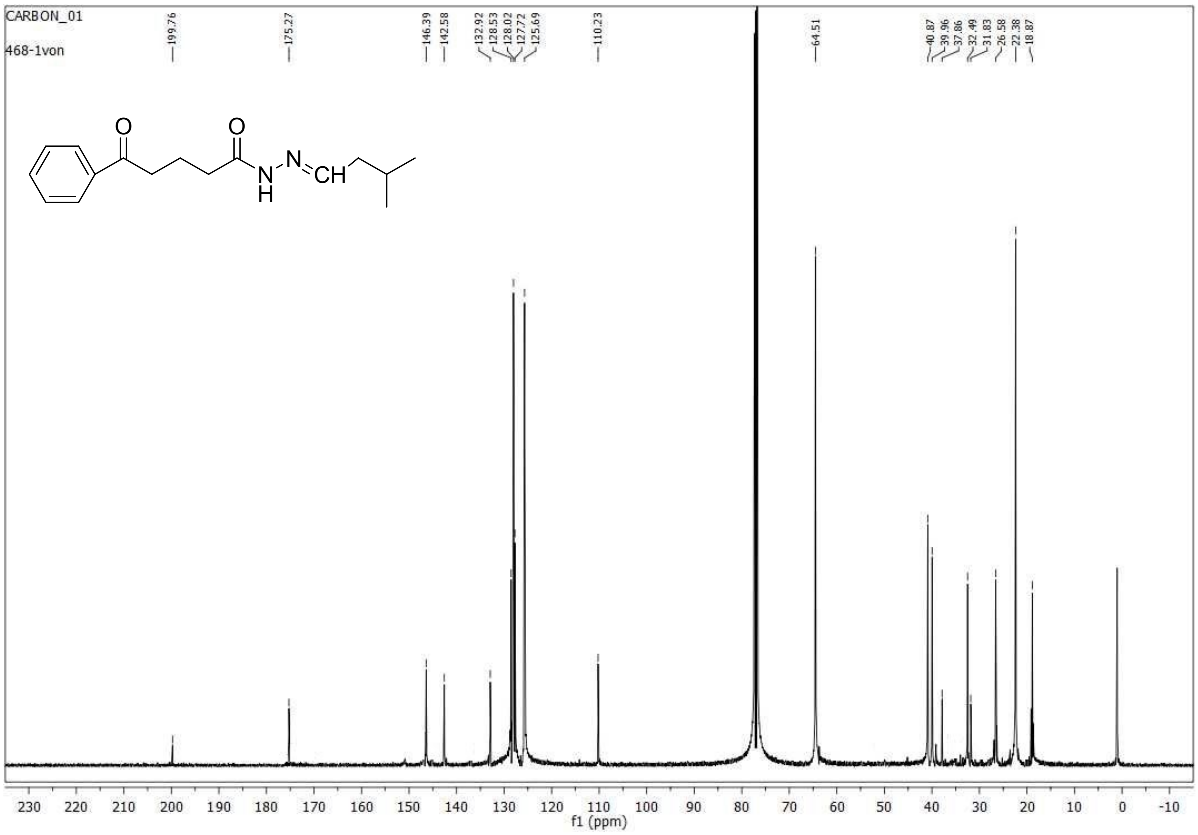

Figure S33. ^{13}C NMR spectrum (126 MHz, DMSO) of **9a**.

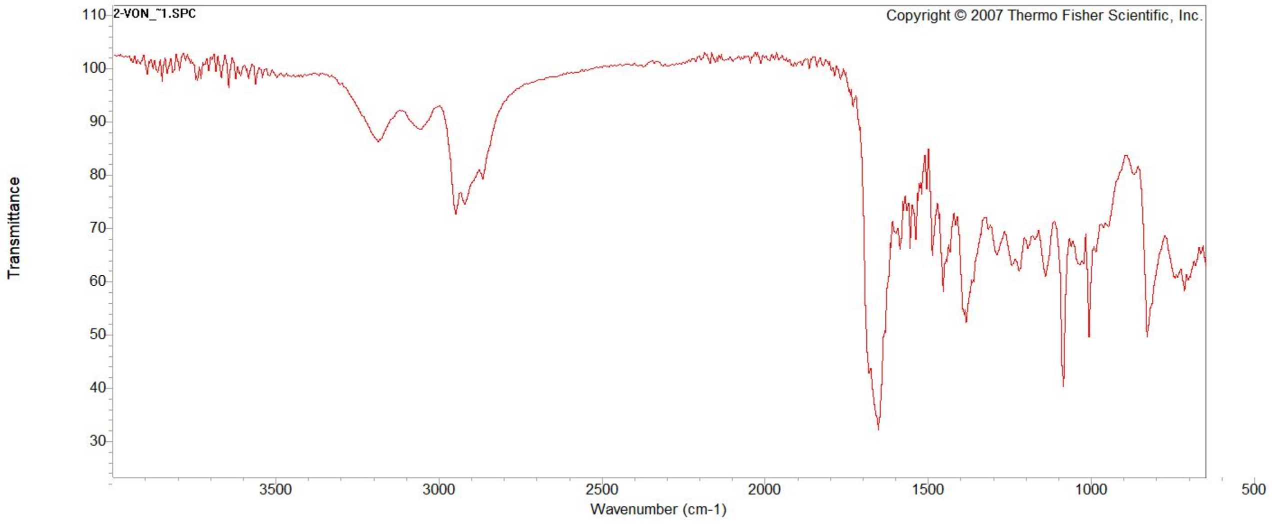

Figure S34. IR spectrum (ATR) of **9b**.

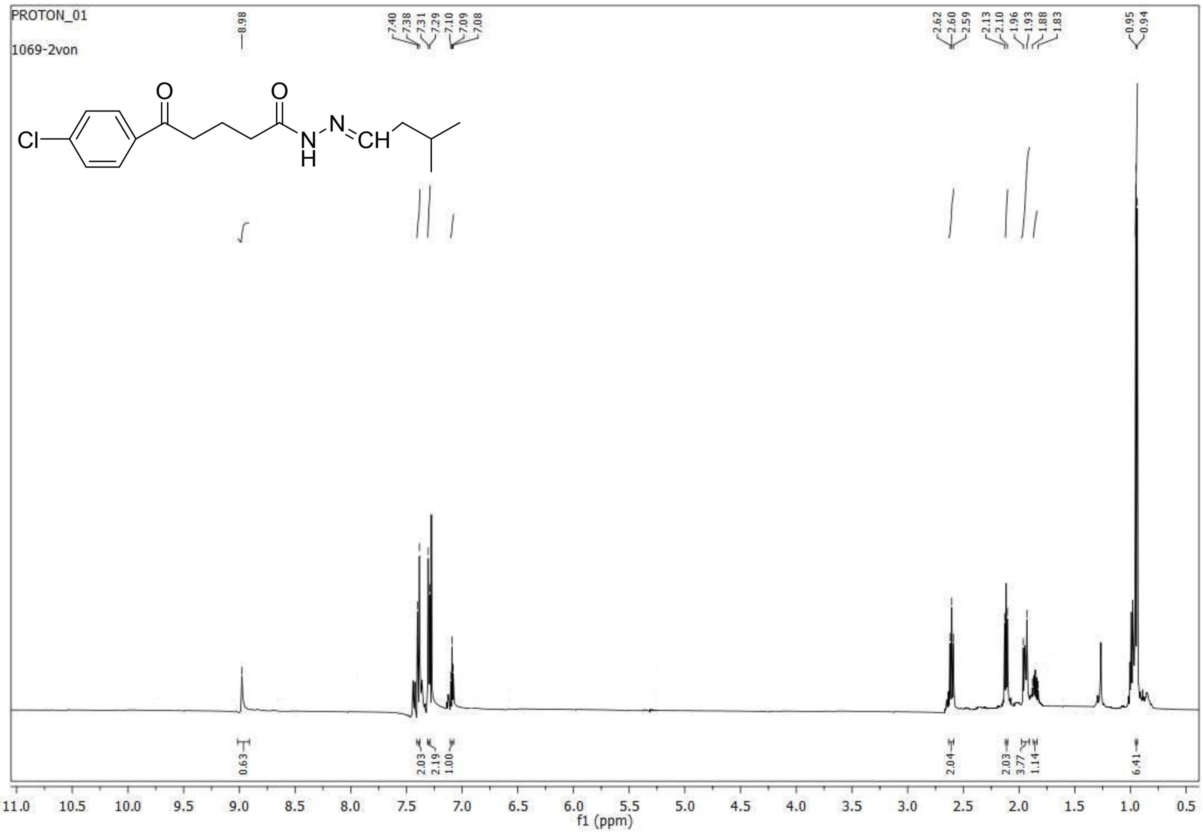

Figure S35. ^{1}H NMR spectrum (500 MHz, DMSO) of **9b**.

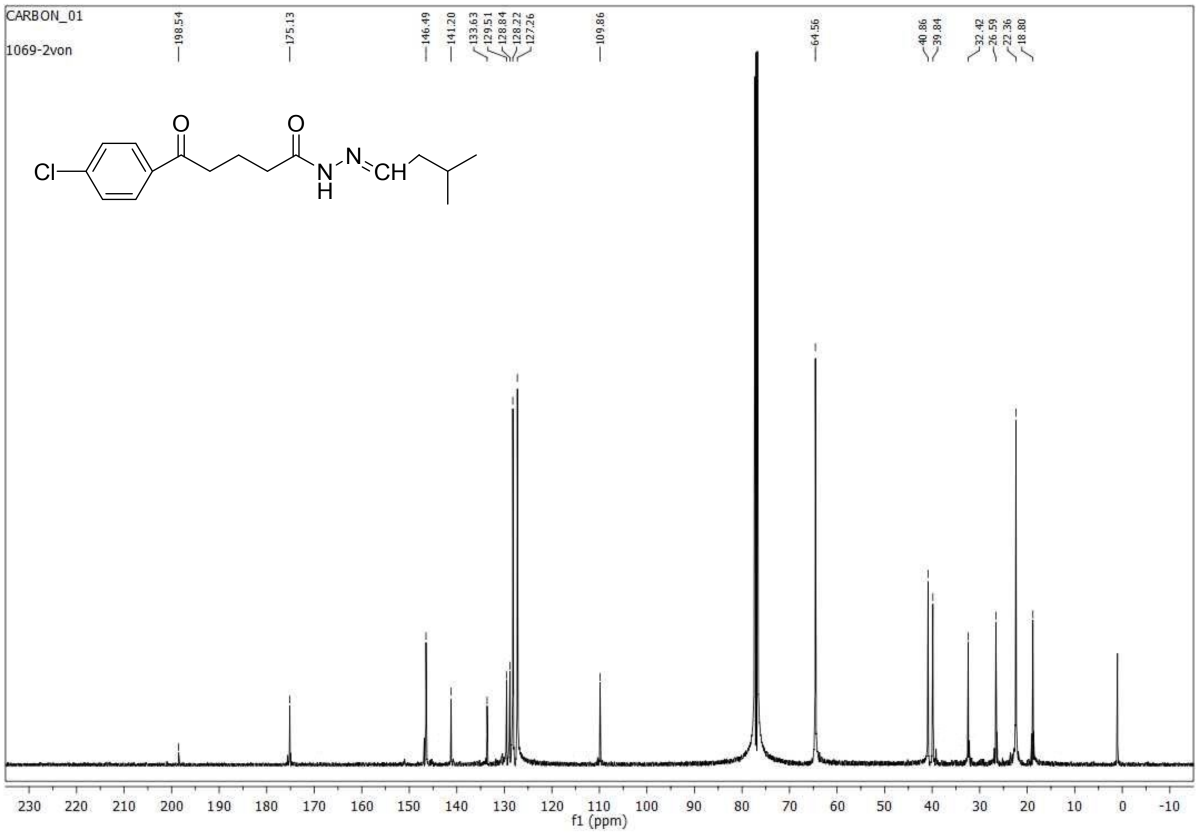

Figure S36. ^{13}C NMR spectrum (126 MHz, DMSO) of **9b**.

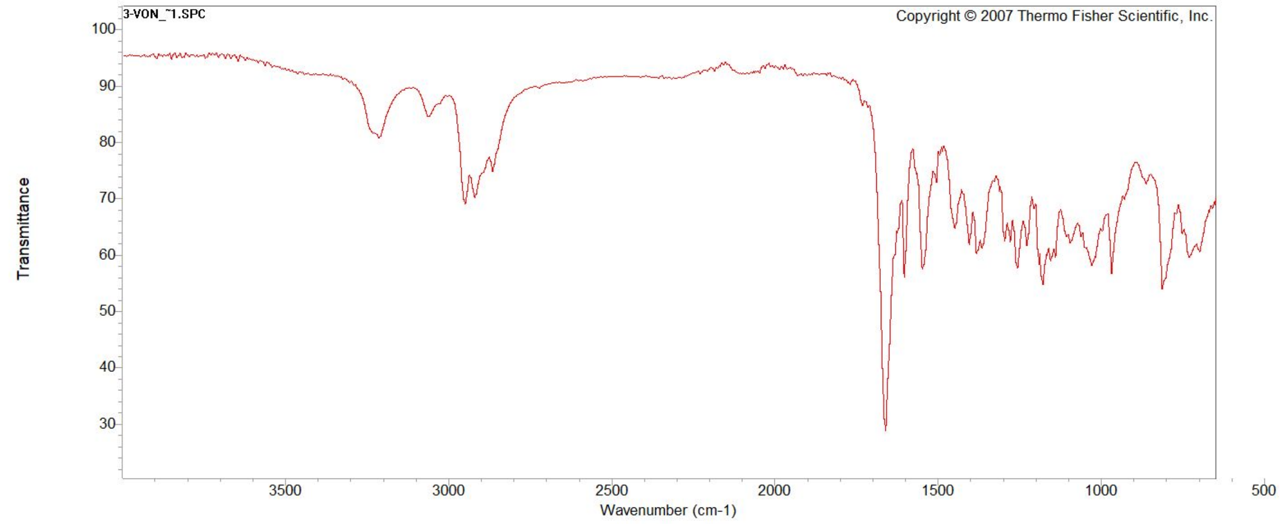

Figure S37. IR spectrum (ATR) of **9c**.

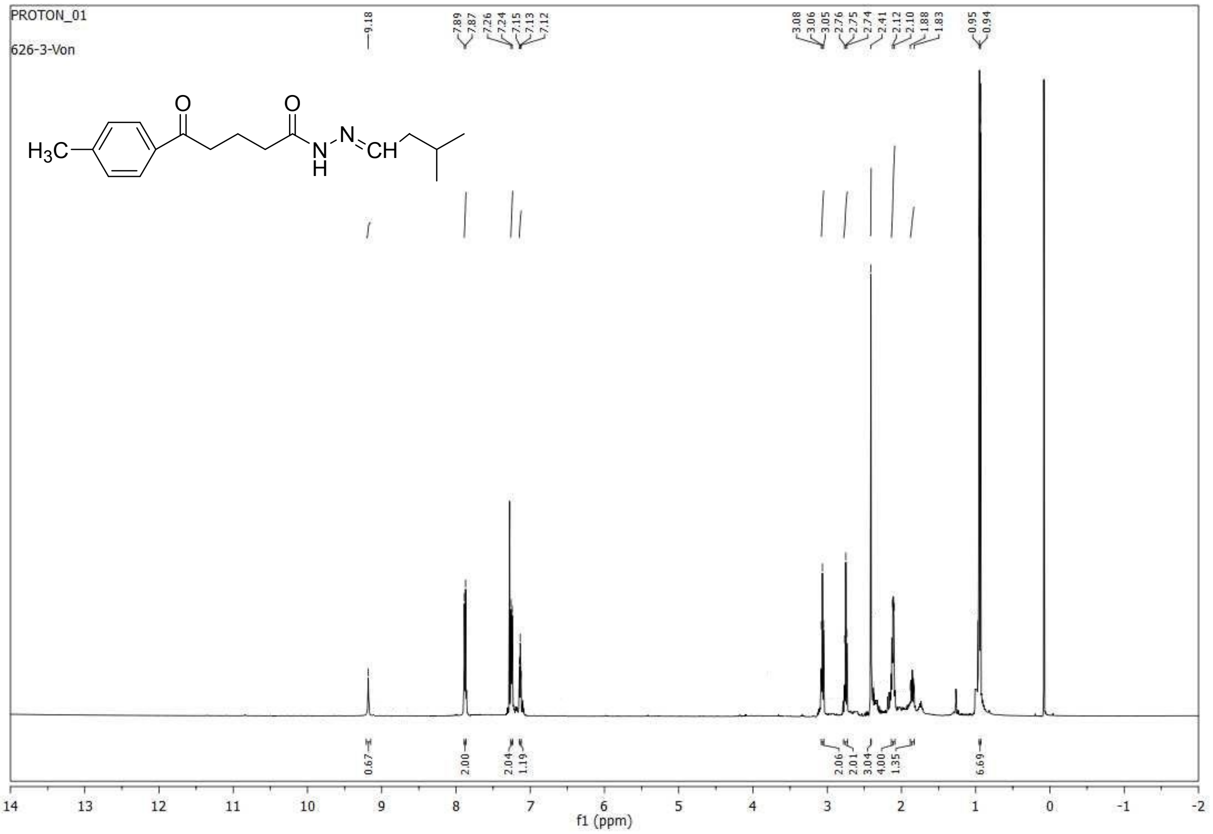

Figure S38. ^{1}H NMR spectrum (500 MHz, DMSO) of **9c**.

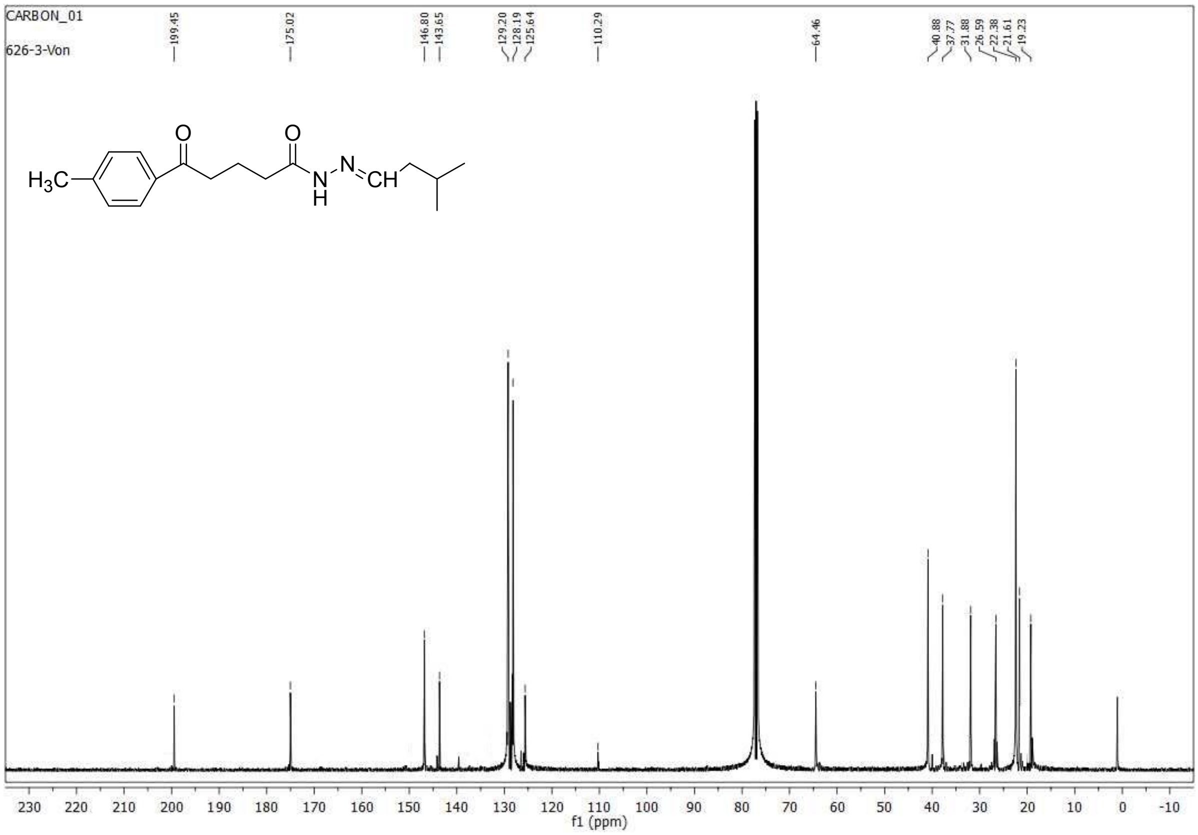

Figure S39. ^{13}C NMR spectrum (126 MHz, DMSO) of **9c**.

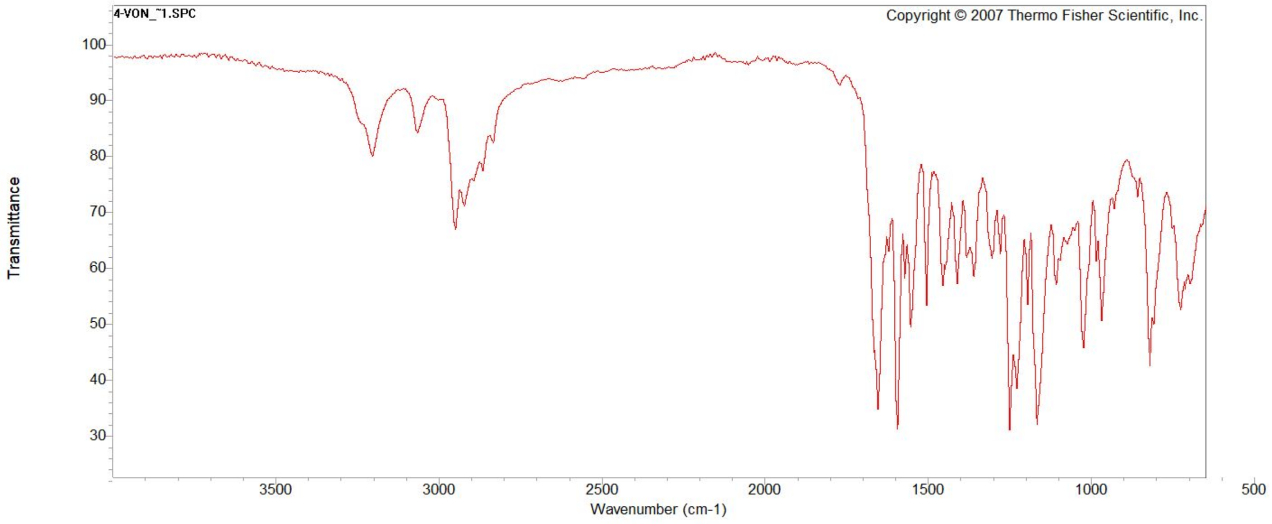

Figure S40. IR spectrum (ATR) of **9d**.

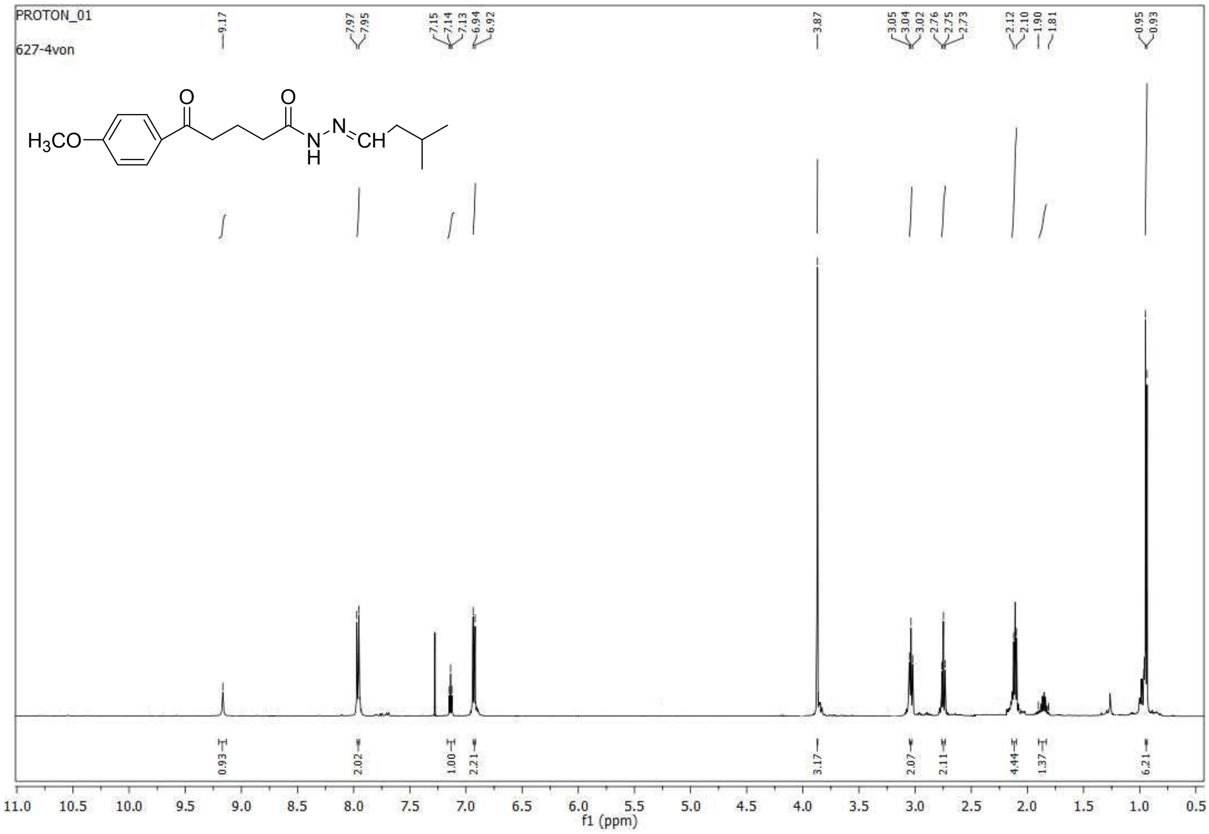

Figure S41. ^{1}H NMR spectrum (500 MHz, DMSO) of **9d**.

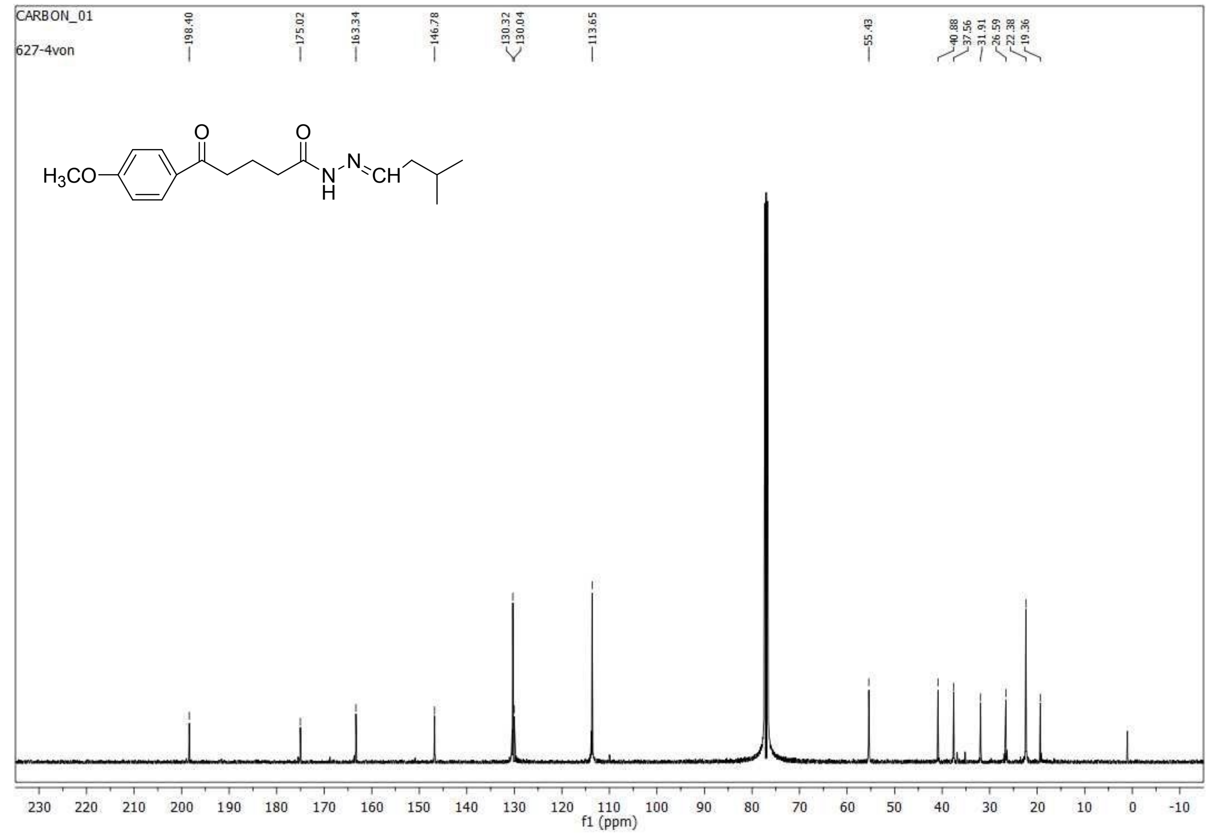

Figure S42. ^{13}C NMR spectrum (126 MHz, DMSO) of **9d**.

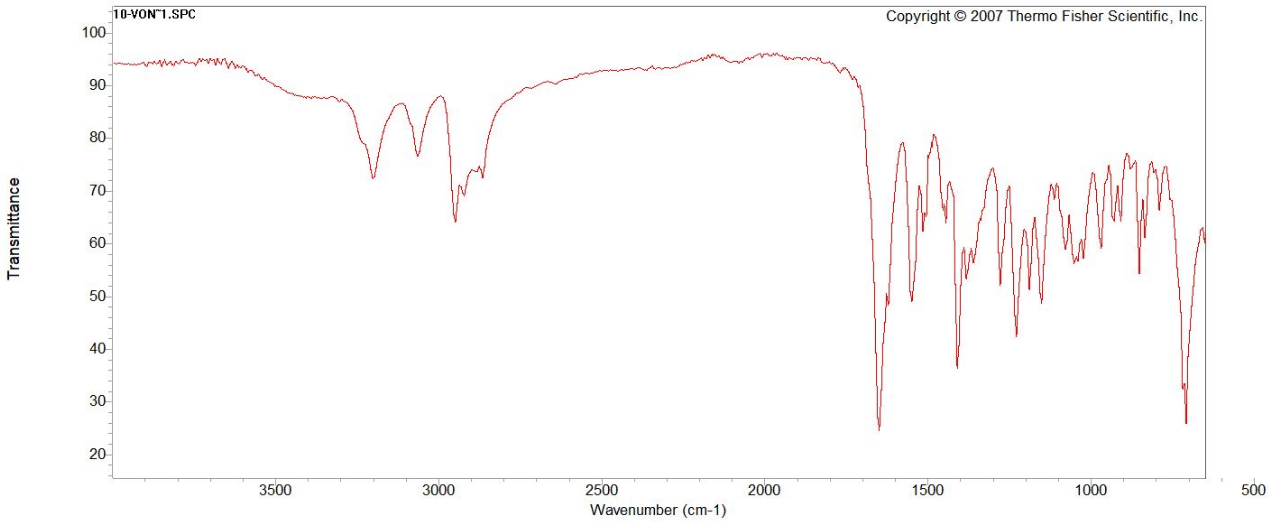

Figure S43. IR spectrum (ATR) of **9e**.

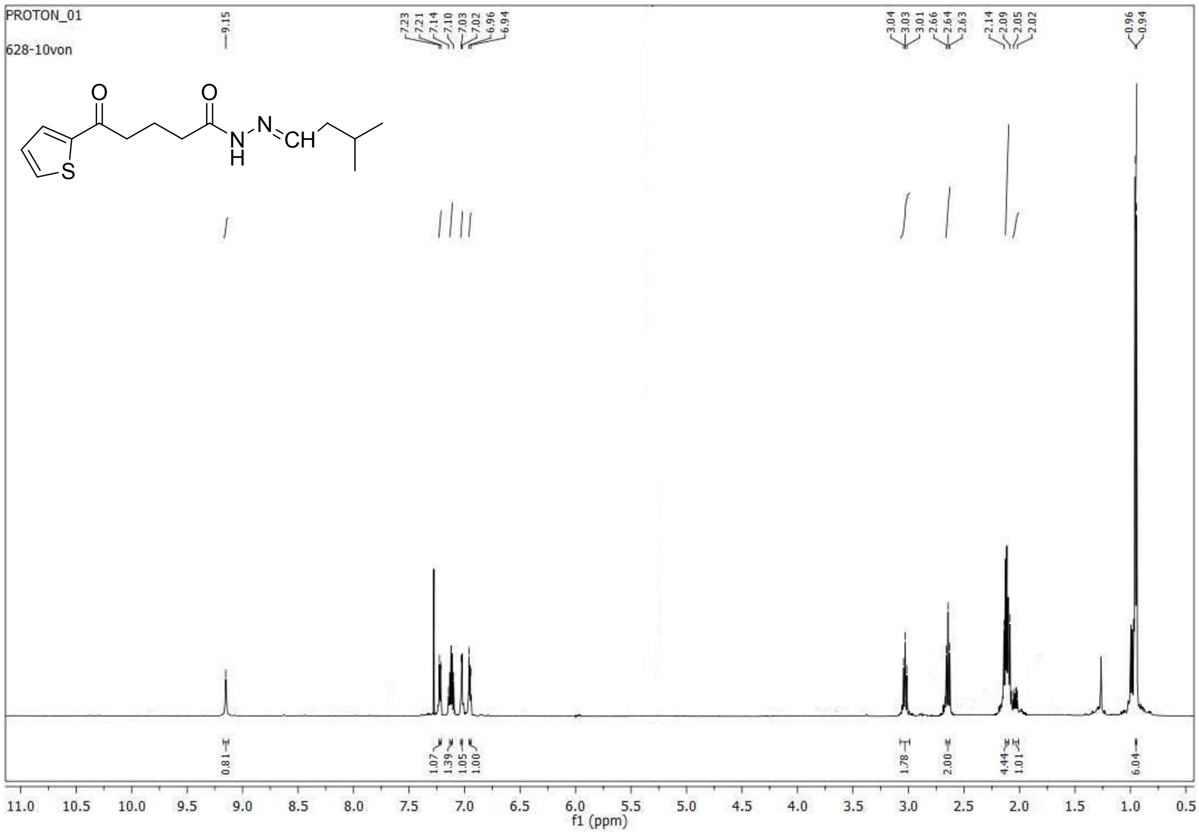

Figure S44. ^{1}H NMR spectrum (500 MHz, DMSO) of **9e**.

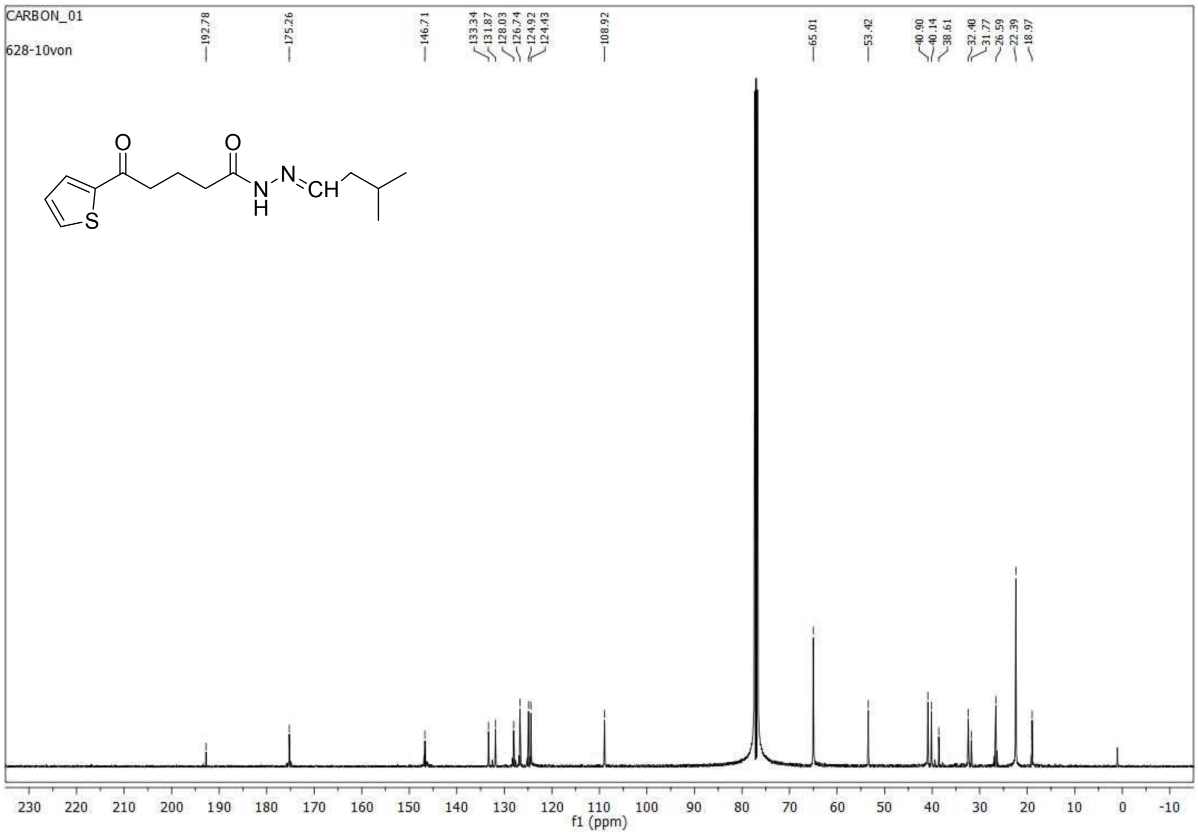

Figure S45. ^{13}C NMR spectrum (126 MHz, DMSO) of **8e**.

HPLC chromatograms of synthesized compounds after purification

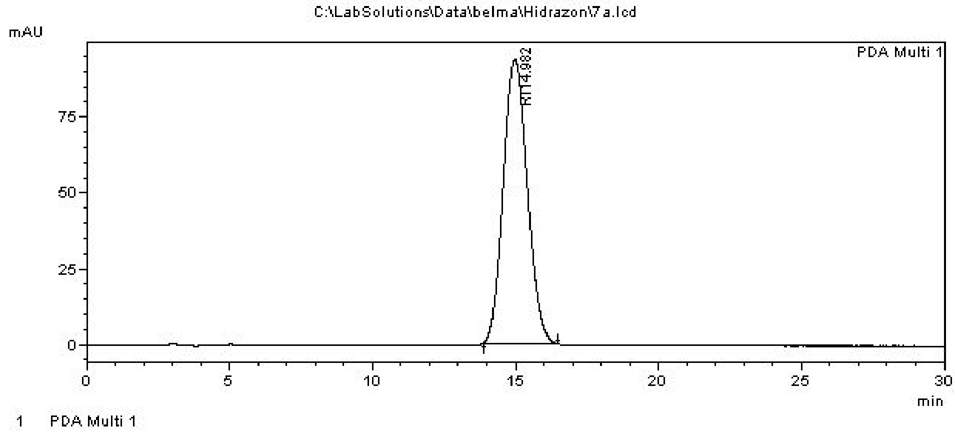

<Results>

PDA Ch1 288nm 4nm

Peak#	Ret. Time	Area	Height	Area %	Height %	Resolution
1	14.982	5492313	93701	100.000	100.000	0.000
Total		5492313	93701	100.000	100.000	

Figure S46: HPLC traces of **7a** after purification showing 100 % purity

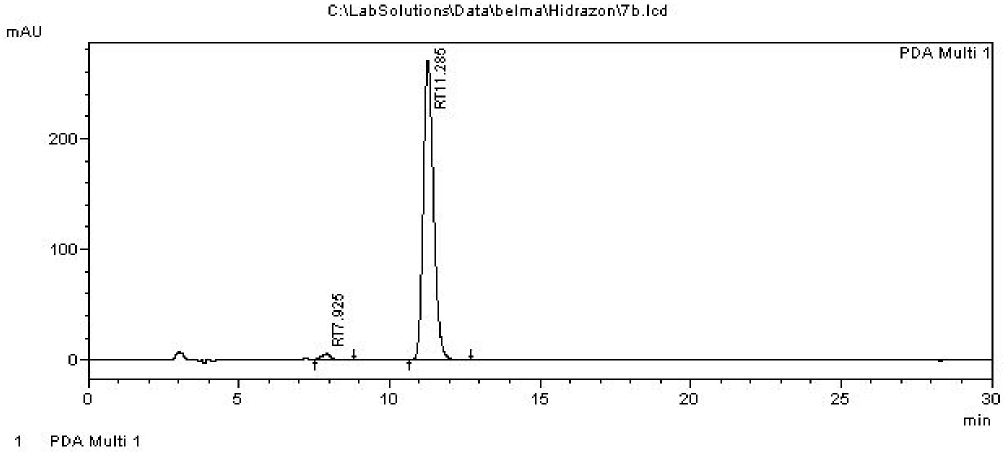

<Results>

PDA Ch1 220nm 4nm

Peak#	Ret. Time	Area	Height	Area %	Height %	Resolution
1	7.925	127817	5285	1.943	1.990	0.000
2	11.285	6449220	260237	98.057	98.010	4.514
Total		6577037	265522	100.000	100.000	

Figure S47: HPLC traces of **7b** after purification showing 98 % purity

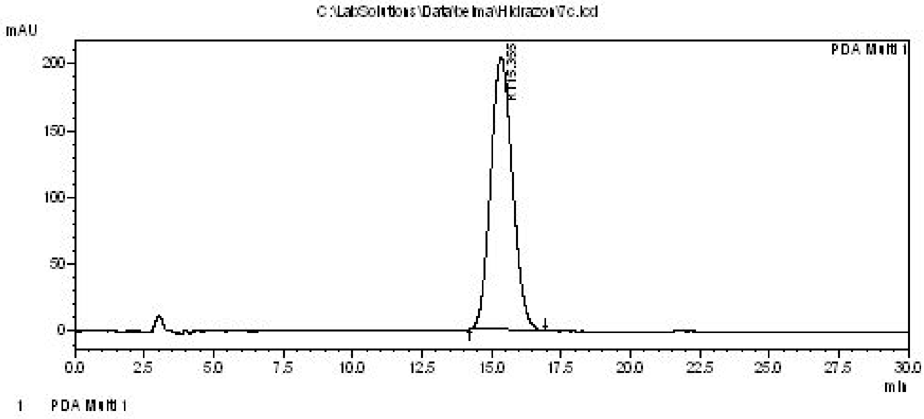

<Results>

PDA Ch1 210nm 4nm

Peak#	Ret. Time	Area	Height	Area %	Height %	Resolution
1	15.355	11167931	204124	100.000	100.000	0.000
Total		11167931	204124	100.000	100.000	

Figure S48: HPLC traces of **7c** after purification showing 100 % purity

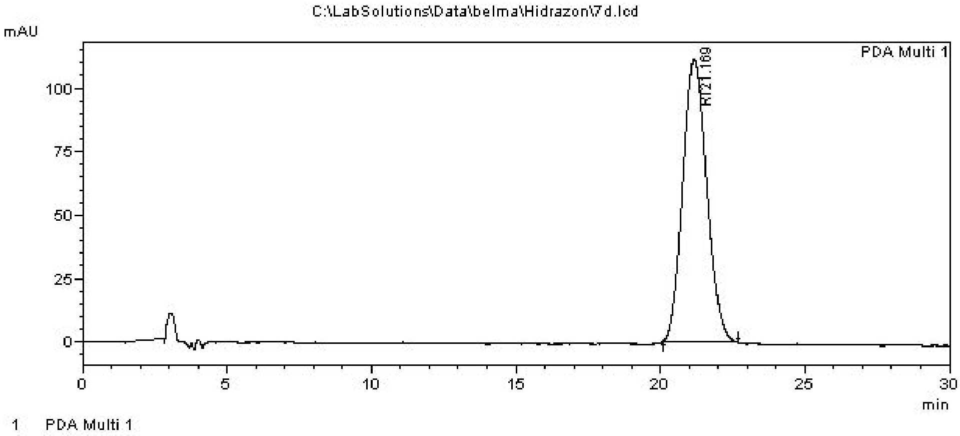

<Results>

PDA Ch1 210nm 4nm

Peak#	Ret. Time	Area	Height	Area %	Height %	Resolution
1	21.169	6461530	111392	100.000	100.000	0.000
Total		6461530	111392	100.000	100.000	

Figure S49: HPLC traces of **7d** after purification showing 100 % purity

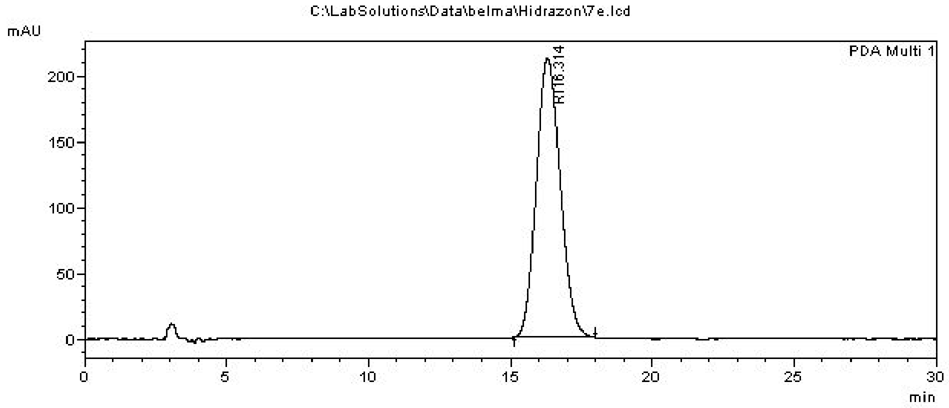

1 PDA Multi 1

<Results>

PDA Ch1 210nm 4nm

Peak #	Ret. Time	Area	Height	Area %	Height %	Resolution
1	16.314	12455594	212225	100.000	100.000	0.000
Total		12455594	212225	100.000	100.000	

Figure S50: HPLC traces of **7e** after purification showing 100 % purity

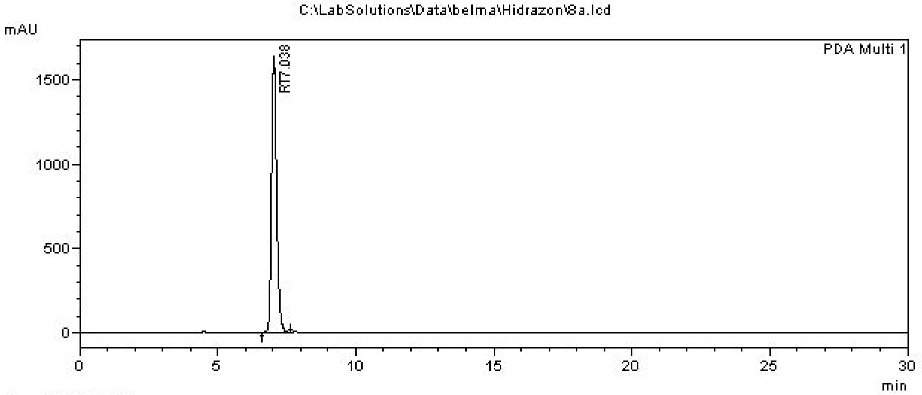

1 PDA Multi 1

<Results>

PDA Ch1 288nm 4nm

Peak #	Ret. Time	Area	Height	Area %	Height %	Resolution
1	7.038	20958254	1640155	100.000	100.000	0.000
Total		20958254	1640155	100.000	100.000	

Figure S51: HPLC traces of **8a** after purification showing 100 % purity

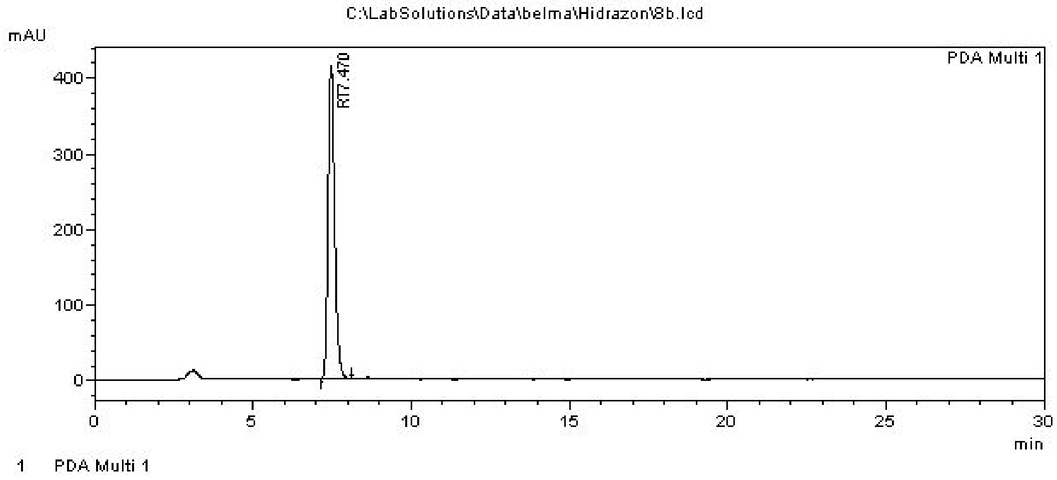

<Results>

PDA Ch1 210nm 4nm

Peak#	Ret. Time	Area	Height	Area %	Height %	Resolution
1	7.470	5866774	413230	100.000	100.000	0.000
Total		5866774	413230	100.000	100.000	

Figure S52: HPLC traces of **8b** after purification showing 100 % purity

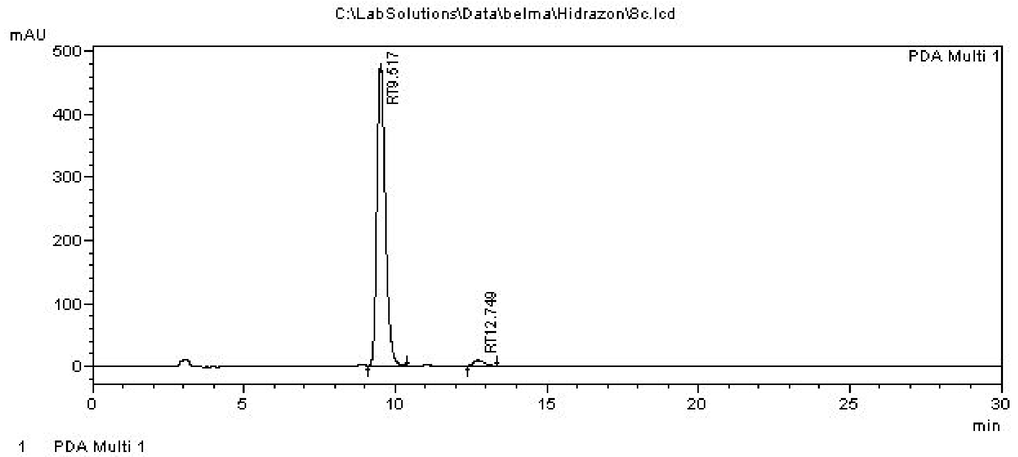

<Results>

PDA Ch1 210nm 4nm

Peak#	Ret. Time	Area	Height	Area %	Height %	Resolution
1	9.517	9105225	478801	97.849	98.288	0.000
2	12.749	200175	8340	2.151	1.712	5.755
Total		9305400	487141	100.000	100.000	

Figure S53: HPLC traces of **8c** after purification showing 97.8 % purity

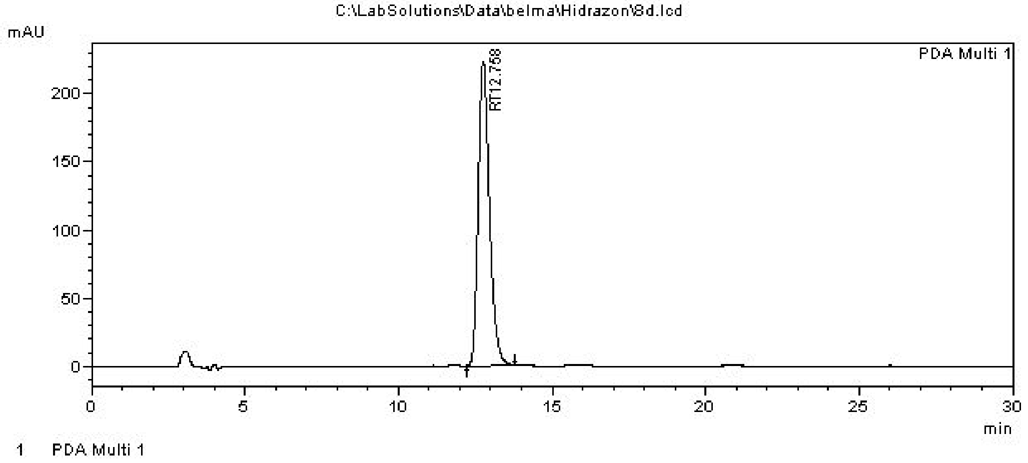

<Results>

PDA Ch1 210nm 4nm

Peak#	Ret. Time	Area	Height	Area %	Height %	Resolution
1	12.758	5647678	223187	100.000	100.000	0.000
Total		5647678	223187	100.000	100.000	

Figure S54: HPLC traces of **8d** after purification showing 100 % purity

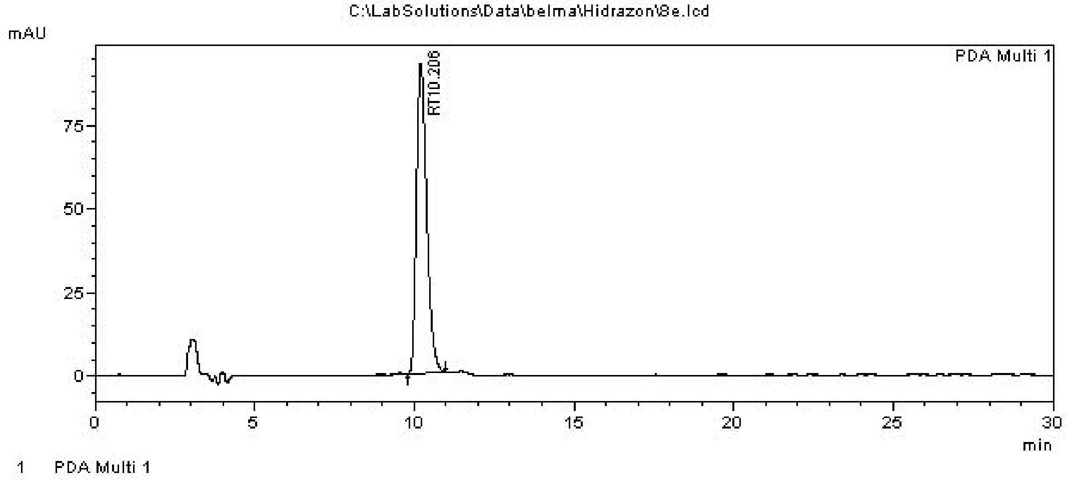

<Results>

PDA Ch1 210nm 4nm

Peak#	Ret. Time	Area	Height	Area %	Height %	Resolution
1	10.206	2050290	92631	100.000	100.000	0.000
Total		2050290	92631	100.000	100.000	

Figure S55: HPLC traces of **8e** after purification showing 100 % purity

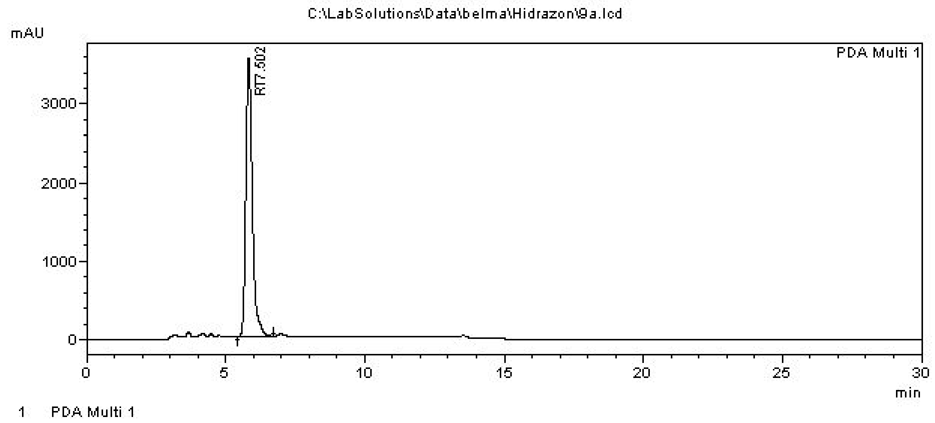

<Results>

PDA Ch1 210nm 4nm

Peak#	Ret. Time	Area	Height	Area %	Height %	Resolution
1	5.648	50106781	3023043	100.000	100.000	0.000
Total		50106781	3023043	100.000	100.000	

Figure S56: HPLC traces of **9a** after purification showing 100 % purity

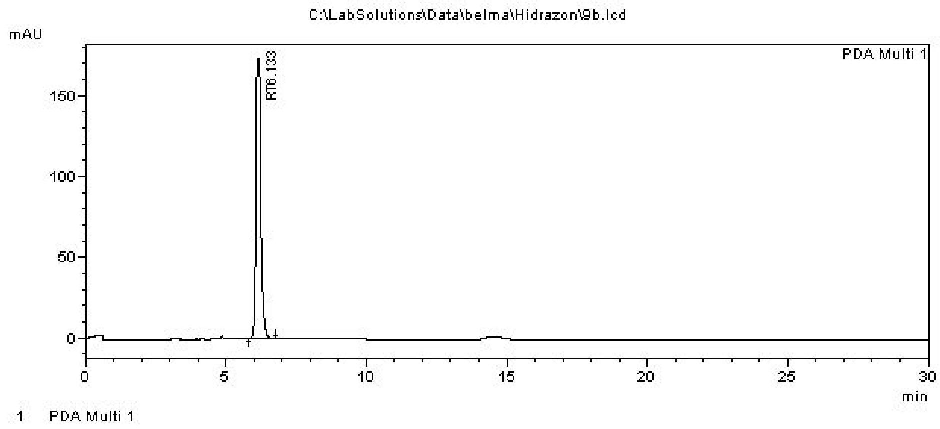

<Results>

PDA Ch1 210nm 4nm

Peak#	Ret. Time	Area	Height	Area %	Height %	Resolution
1	6.133	2071207	157165	100.000	100.000	0.000
Total		2071207	157165	100.000	100.000	

Figure S57: HPLC traces of **9b** after purification showing 100 % purity

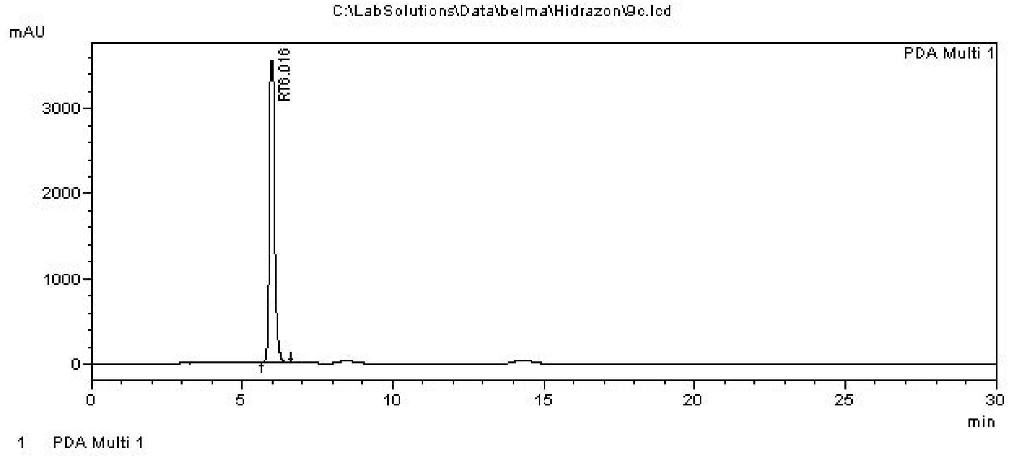

<Results>

PDA Ch1 210nm 4nm

Peak#	Ret. Time	Area	Height	Area %	Height %	Resolution
1	6.016	43514868	3543654	100.000	100.000	0.000
Total		43514868	3543654	100.000	100.000	

Figure S58: HPLC traces of **9c** after purification showing 100 % purity

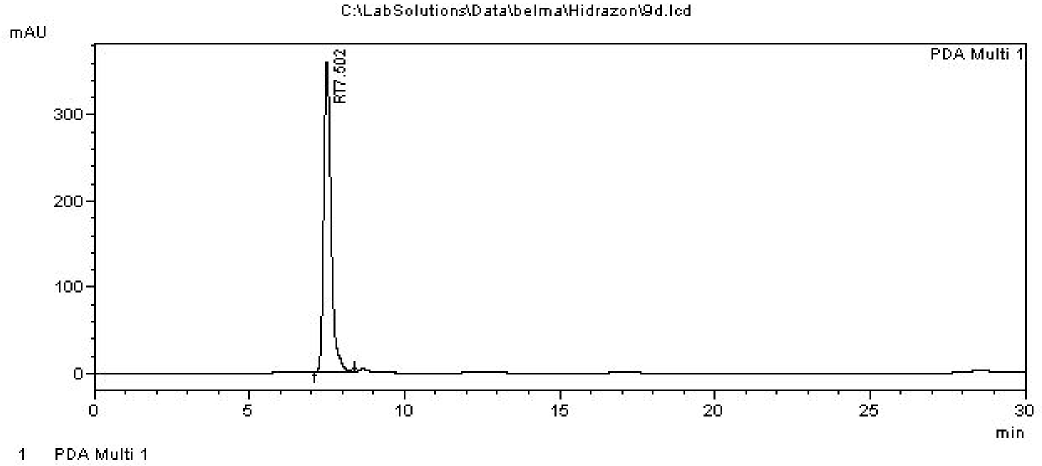

<Results>

PDA Ch1 288nm 4nm

Peak#	Ret. Time	Area	Height	Area %	Height %	Resolution
1	7.502	5828923	359730	100.000	100.000	0.000
Total		5828923	359730	100.000	100.000	

Figure S59: HPLC traces of **9d** after purification showing 100 % purity

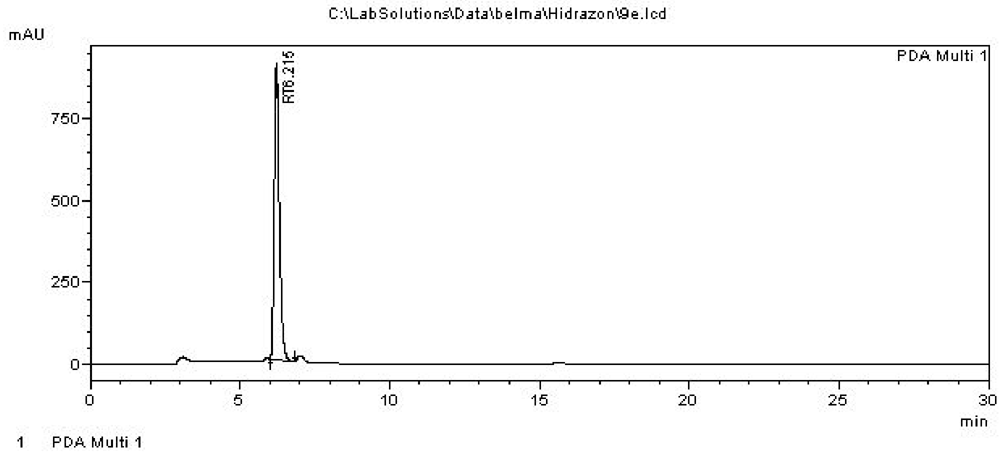

<Results>

PDA Ch1 210nm 4nm

Peak#	Ret. Time	Area	Height	Area %	Height %	Resolution
1	6.215	10902101	906505	100.000	100.000	0.000
Total		10902101	906505	100.000	100.000	

Figure S60: HPLC traces of **9e** after purification showing 100 % purity
